# CD248 acts as a mechanosensory switch in fibroblast subsets to establish distinct pathological niches in renal fibrosis

**DOI:** 10.1038/s41467-026-72187-0

**Published:** 2026-05-06

**Authors:** Chao Xu, Shaojie Liu, Yike Zhou, Fa Yang, Zhengxuan Li, Kai Gan, Yu Li, Keying Zhang, Tong Lu, Hongtao Song, Jun Jiang, Jiayang Qin, Zhihao Hu, Ruochen Qi, Shuaijun Ma, Changhong Shi, Rui Zhang, Dailing Si, Weihong Wen, Donghui Han, Weijun Qin

**Affiliations:** 1 Department of Urology, Xijing Hospital, The Fourth Military Medical University, Xi’an, China; 2 Department of Urology, Daping Hospital, Army Medical University, Chongqing, China; 3 West China School of Medicine, West China Hospital, Chengdu, China; 4 Division of Cancer Biology, Laboratory Animal Center, The Fourth Military Medical University, Xi’an, China; 5 Department of Immunology, State Key Laboratory of Cancer Biology, The Fourth Military Medical University, Xi’an, China; 6 Military Medical Innovation Center, The Fourth Military Medical University, Xi’an, Shaanxi China; 7 School of Life Sciences and Technology, Northwestern Polytechnical University, Xi’an, China

**Keywords:** Target identification, Kidney diseases

## Abstract

Although the heterogeneity and plasticity of fibroblasts are recognized hallmarks of tissue fibrosis, their specific contributions to renal fibrosis progression and the therapeutic validity of targeting key effector subsets remain key unanswered questions. Here, we revealed that diverse, functionally exclusive fibroblast subpopulations actively shape their local microenvironment. Pro-inflammatory fibroblasts (i-Fibs) construct immune-active areas, while pro-fibrotic fibroblasts (ECM-Fibs) generate fibrogenic zones. These two distinct microenvironments form a spatial mosaic, occupying mutually exclusive territories within the kidney tissue. We identified mechanotransduction as a signal governing the switch from i-Fibs to the pathogenic ECM-Fib phenotype. Through unbiased bioinformatics and genetic tools, we pinpointed CD248 as a specific cell-surface protein on this matrix-producing subset. Mechanistically, CD248, via its C-type lectin-like domain, senses the disordered matrix, promoting focal adhesion assembly and YAP nuclear translocation through an IQGAP1/ARF6-GTP-dependent axis, leading to a sustained feedback loop between aberrant matrix and myofibroblasts activation. A monoclonal antibody targeting CD248, IgG78, effectively interrupted this feedback loop, mitigating fibrogenesis both in vitro and in vivo in male mice. Collectively, this study established that fibroblast heterogeneity drives pathological niche specification in the fibrotic kidney and validated CD248 as a promising therapeutic target to counteract tissue fibrosis by disrupting aberrant mechanosignaling.

## Abstract (web summary)

Fibroblast heterogeneity is a hallmark of fibrosis including in the kidney, but their precise roles in driving renal fibrosis are incompletely understood. Here the authors report that a switch to a CD248⁺ extra cellular matrix -producing fibroblast subset contributes to kidney fibrosis, and blocking CD248 with a monoclonal antibody mitigates the phenotype in male mice.

## Introduction

Renal fibrosis is a common pathological alteration in the progression of nearly all forms of chronic kidney disease (CKD), including transplantation-related ischemia-reperfusion injury, ultimately leading to end-stage kidney diseases^1,2^. Fibroblasts play a critical role in the progression of CKD, engaging in complex processes that transition from inflammation to scar tissue formation^3^, despite their central role in renal fibrosis, pharmacological therapies specifically targeting fibroblasts have yet to be approved^4^. The primary reason for this is likely to be our insufficient understanding of the complex heterogeneity of fibroblasts within the renal fibrogenic niche^5–8^. Therefore, understanding the functional subsets of fibroblasts and identifying the effectors of fibrotic progression are vital to develop effective therapies.

A hallmark of CKD progression is the development of a heterogeneous pathological landscape, often showed as a ‘mosaic’ of distinct microenvironments, within which zones of active inflammation are frequently interspersed with areas of fibrogenesis^6^. This spatial heterogeneity at the tissue level directs an underlying functional heterogeneity of cellular components. Fibroblasts, known for their remarkable phenotypic plasticity, are central candidates for orchestrating this complex process^9,10^. Indeed, in contexts such as cancer and autoimmune diseases, fibroblasts are recognized to differentiate into functionally diverse subsets, capable of modulating inflammation, presenting antigens, or driving matrix remodeling^8,11^. However, whether distinct fibroblast states drive the pathological evolution from inflammatory foci to established scar tissue in the kidney, remain poorly understood.

A self-perpetuating cycle between myofibroblasts and their biophysical microenvironment is increasingly recognized as a central feature of fibrotic diseases^12^. This process, often termed “paratensile signaling”^13^, begins as fibroblasts progressively activate and remodel the tissue stroma. This remodeled, mechanically aberrant environment then triggers a feedback mechanism, compelling adjacent fibroblasts to differentiate into myofibroblasts^13,14^. This loop ultimately emerges as the dominant driver of pathology within the fibrogenic niche, leading to impaired tissue repair^11,14,15^. This paradigm establishes the foundation for “Mechano-medicine”, a strategy aimed at interrupting this mechanobiological cycle, rather than targeting classical biochemical pathways. Nevertheless, its therapeutic potential in the context of renal fibrosis remains largely unexplored.

Membrane receptors are among the most favored molecules in precision medicine, and the development of specific monoclonal antibodies (mAbs) is crucial for various therapeutic strategies^16^. Mechanosensitive receptors are important regulators that enable cells to sense aberrant ECM properties from the microenvironment, modulating the activation of mechanical signals such as focal adhesion assembly and cytoskeletal rearrangement. Although receptors like integrin αvβ3, α5β1, and Piezo1 play critical roles in fibroblast activation and phenotypic transition^17,18^, their widespread expression and lack of disease specificity limit clinical application^19^. Therefore, identifying membrane molecules specific to activated fibroblast subpopulations, sensitive to ECM changes, is essential for blocking mechanotransduction-mediated fibroblast activation and preventing renal fibrosis progression.

Herein, we aimed to uncover the heterogeneous subpopulations of fibroblasts during the progression of CKD. Interestingly, distinct fibroblast subsets actively contributed to shape their local environment, creating mutually exclusive and mosaic-like niches. Among diverse subpopulations, we found mechanotransduction-induced fibroblasts subsets responsible for fibrogenesis. With unbiased bioinformatics and genetic tools, we demonstrated that CD248 is not only a specific cell-surface membrane protein for this subset, but also plays a role in establishing the biomechanical feedback loop between myofibroblasts and aberrant ECM, promoting stromal stiffness. Administration of IgG78, a mAb targeting CD248, effectively inhibited mechanotransduction and fibrogenesis both in vitro and in vivo. Collectively, our findings highlight a potential therapeutic strategy for precisely targeting fibroblast mechanotransduction and fibrosis within a complex pathological landscape during renal fibrogenesis.

## Results

### Identification of phenotypic heterogeneity of fibroblasts in the fibrogenic niche after kidney injury

**Fig. 1:**
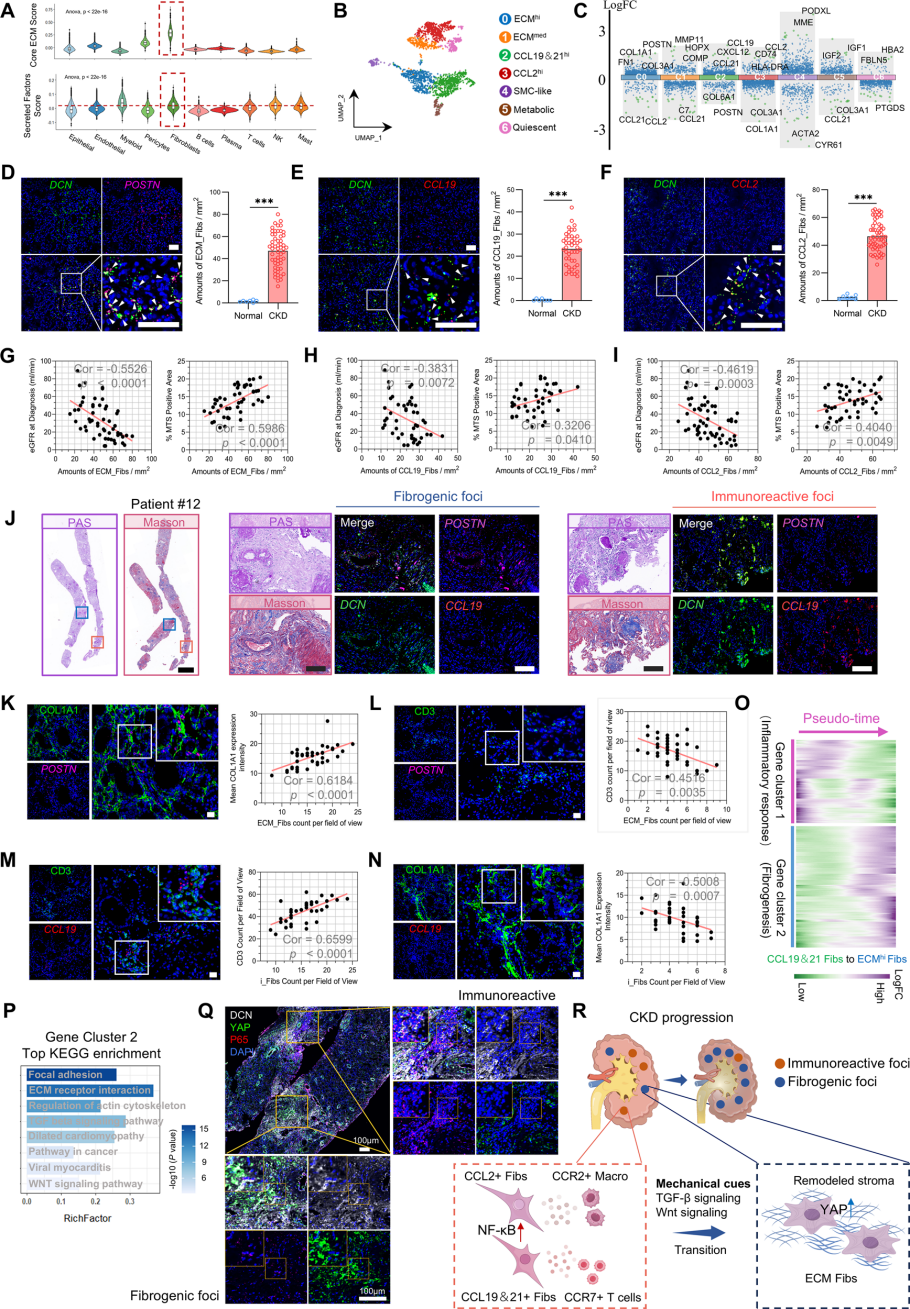
Functionally diverse fibroblast subsets shape the local pathological characteristics of foci during CKD progression. **A** Violin plot showing gene sets score among major cell types. Statistical significance was determined using one-way ANOVA. **B** UMAP plot of fibroblast subpopulations. **C** Differential gene expression analysis showing up- and down-regulated genes across all fibroblasts clusters. Genes with an adjusted *P* < 0.01 are highlighted in green, while an adjusted *P* ≥ 0.01 are indicated in blue. Statistical significance was determined using a two-sided Wilcoxon rank-sum test with Bonferroni correction. **D**–**F** Representative images of RNAscope staining for ECM Fibs (**D**, *n* = 7 normal samples and *n* = 56 CKD samples). CCL19& 21^hi^ Fibs (**E**, *n* = 7 normal samples and *n* = 45 CKD samples.) and CCL2^hi^ Fibs (**F**, *n* = 7 normal samples and *n* = 56 CKD samples.) (left), and quantification analysis (right). Correlation analysis of the abundance of ECM Fibs (**G**), CCL19& 21^hi^ Fibs (**H**) or CCL2^hi^ Fibs (**I**) with either eGFR (left) or collagen deposition area (right) in clinical CKD samples. **J** Analysis of consecutive kidney sections using RNAscope, PAS, and Masson staining. **K**–**N** Representative image of combined RNAscope and immunofluorescence staining (left), and the correlation analysis (right). **O** Heatmap showing altered genes clusters (clusters 1 and clusters 2) during the differentiation trajactory. **P** Top enriched KEGG function of gene cluster 2. Top enriched KEGG function of gene cluster 2. *P values* were calculated using a one-sided Fisher’s exact test. **Q** Representative multi-immunofluorescent staining in fibrotic kidney. **R** Schematic representation of the predominant functional fibroblast subpopulations within immunoreactive and fibrogenic lesions. With disease progression, differentiation of ECM-producing fibroblasts (ECM-Fibs), mediated by YAP and driven by mechanical signals, contributes to the diffuse advancement of fibrogenic lesions. All Scale bar, 100 μm. ***P < 0.001. All data are represented as mean ± s.d. Statistics were calculated using two-tailed, unpaired Student’s *t* test (**D**–**F**), and two-sided Pearson correlation analysis (**G**–**I**, and **K**–**N**). Source data are provided as a Source Data file.

To identify fibroblasts heterogeneity during renal fibrogenesis in an unbiased manner, we re-analyzed single-cell RNA sequencing (ScRNA-seq) data^20^, and identified 10 major clusters of non-proximal tubular cells in kidney fibrotic tissues based on canonical markers (Supplementary Fig. 1A–C). Furthermore, by calculating the score for core ECM score and secreted inflammatory factors using the matrisome gene set^21,22^, we demonstrated that fibroblasts serve as crucial contributors to ECM production and immunomodulation in the microenvironment of the injury kidney (Fig. 1A and Supplementary Fig. 1D). To decipher the fibroblast heterogeneity in the fibrogenic niche, we firstly used canonical markers (*RGS5* and *PDGFRB* for pericytes, *DCN*, *LUM*, and *COL1A2* for fibroblasts) to distinguish fibroblasts from mesenchymal cells. Then, differentially expressed genes and subsequent gene ontology (GO) analysis were conducted to reveal the distinct heterogeneous functions of the fibroblast subclusters (Fig. 1B, C, and Supplementary Fig. 1E, F, and Supplementary Data 1, 2). Considering that fibrosis and inflammation are the major pathological features of the sustained progression of fibrosis^12,19,23^, we mainly focused on the subsets with pro-fibrotic (C0 and C1) and pro-inflammatory phenotypes (C2 and C3).

Interestingly, both of the ECM-associated fibroblasts (ECM-Fibs) and inflammatory fibroblasts (i-Fibs) exhibited significant phenotypic exclusivity: the former highly expresses ECM-related genes with a weak inflammatory phenotype, while the latter lacks ECM expression, but highly expresses pro-inflammatory cytokines (Supplementary Fig. 1G, H). To investigate the role of fibroblasts subsets in fibrosis progression, we constructed and evaluated gene signatures specific to distinct fibroblast subpopulations based on the ScRNA-seq data (Supplementary Fig. 2A, B, and Supplementary Data 3). Using public bulk RNA-seq data from CKD patients, the inferred abundance of diverse subsets was positively correlated with the expression of major ECM components (Supplementary Fig. 2C). These findings indicated that fibroblast-driven inflammation and aberrant matrix repair are crucial drivers of the fibrotic process.

### Fibroblasts establish functionally distinct and mutually exclusive pathological niches in injured kidneys

To characterize the spatial distribution of distinct fibroblast subsets during renal fibrotic progression, we performed RNAscope on serial sections of fibrotic renal tissue. Probes for *DCN*, *POSTN*, *CCL19*, and *CCL2* were used to identify total fibroblasts, ECM-Fibs (*POSTN*), and two major i-Fib subsets: C2 (*CCL19*) and C3 (*CCL2*), respectively. Compared to normal kidney tissue, the abundance of ECM-Fibs, C2, and C3 subsets was markedly increased in fibrotic areas (Fig. 1D, F and Supplementary Fig. 3). Furthermore, correlation analysis revealed that the abundance of both ECM-Fibs and i-Fibs was positively correlated with both collagen deposition and the severity of renal dysfunction, as measured by the estimated glomerular filtration rate (eGFR) (Fig. 1G, I and Supplementary Data 4).

Our results have shown a significant expression of CCL19, CCL21, and CXCL12 in CCL19 & 21^hi^ Fibs compared with the other subsets. CCR7, the shared receptor for CCL19 and CCL21, is critical for T and B cell homing. We found that CCR7^+^ T cell infiltration was increased in CKD tissue compared to normal controls (Supplementary Fig. 4A). These above results suggested that CCL19 & 21^hi^ Fibs contribute to the recruitment of CCR7^+^ T cells in foci of CKD tissues. The spatial heterogeneity of pathological lesions is a hallmark of CKD progression^6^. This led us to hypothesize that such focal heterogeneity is driven by the local predominance of functionally distinct fibroblast subsets. Firstly, we observed a striking spatial mutual exclusivity between immuno-active niches, characterized by T cells infiltration, and fibrogenic niches, marked by COL1A1 deposition (Supplementary Fig. 4B). Combined histological and RNAscope analysis of serial kidney sections revealed a clear spatial segregation between *POSTN*- and *CCL19*-expressing fibroblasts subsets. Specifically, *POSTN* expression was co-localized with areas of fibrogenesis, while *CCL19* expression was found in regions of dense immune cell infiltration (Fig. 1J). To quantify the relationship between fibroblast subsets and niche composition, we performed combined RNAscope and immunofluorescence analysis. Our data revealed that within local pathological niches, stromal *POSTN* expression was tightly coupled with COL1A1 deposition while being inversely correlated with CD3^+^ T cell density (Fig. 1K, L), whereas *CCL19* expression was strongly associated with CD3^+^ T cell infiltration and negatively with COL1A1 deposition (Fig. 1M, N). Collectively, these findings demonstrated that ECM-Fibs and i-Fibs orchestrate the formation of distinct fibrogenic and immuno-active niches, respectively, highlighting the important role of fibroblast heterogeneity in shaping local pathological lesions.

### The transition of i-Fibs to ECM-Fibs is driven by mechanosensitive fibroblast activation in response to aberrant stromal properties

To investigate the plasticity between fibroblast subsets, we performed trajectory inference analysis. This analysis suggested a potential differentiation path from i-Fibs towards ECM-Fibs, characterized by a progressive shift in gene expression: a gradual decrease in inflammatory chemokines was paralleled by an increase in extracellular matrix components along the pseudotime trajectory (Supplementary Fig. 5A–C). To understand the biological processes driving this transition, we examined the dynamic gene expression patterns along this trajectory. We identified two major, functionally distinct gene clusters based on their expression dynamics (Fig. 1O). Functional enrichment analysis revealed that gene cluster 1, which was highly expressed early in the trajectory (i-Fib state), was associated with pro-inflammatory signatures, including “Cytokine-cytokine receptor interaction” (Supplementary Fig. 5D). In contrast, gene cluster 2, with expression peaking late in the trajectory (ECM-Fib state), was enriched for mechanosensitive and pro-fibrotic pathways, such as “ECM-receptor interaction”, “Focal adhesion”, and “Regulation of actin cytoskeleton” (Fig. 1P).

We next investigated the core effector factor governing these fibroblast states, focusing on NF-κB and YAP as the key effectors of inflammation and mechanosignaling, respectively^12,24^. Pathway scoring analysis showed that NF-κB signaling was preferentially active in i-Fibs, whereas YAP signaling was predominantly enriched in ECM^hi^ Fibs (Supplementary Fig 5E). Through multiplex immunofluorescence, we demonstrated a mutual exclusivity between nuclear YAP and NF-κB in the stromal compartments of CKD tissue (Fig. 1Q), similar with the results in Fig. 1J. Finally, atomic force microscopy (AFM) analysis demonstrated that progressive matrix stiffening accompanies fibrotic progression, implicating aberrant mechanosignaling as a pivotal mechanism driving the activation of ECM^hi^ Fibs and subsequent renal fibrosis (Fig. 1R and Supplementary Fig. 5F–N).

### CD248+ myofibroblasts represent the terminal fibroblasts subtype responsible for stromal remodeling in CKD patients

**Fig. 2:**
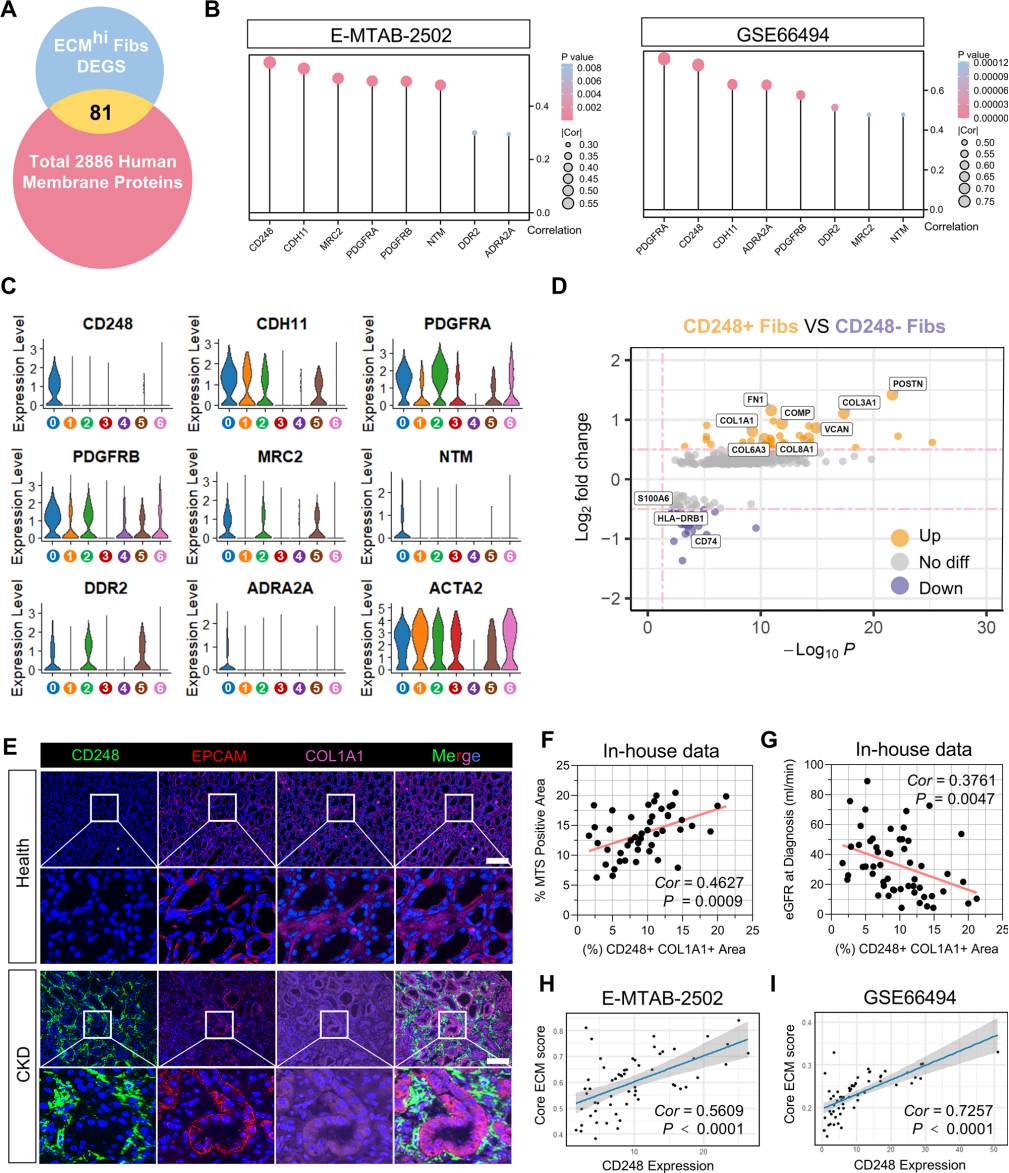
CD248 is specific cell-surface membrane protein of ecm^hi^ Fibs. **A** Overlap analysis was performed to seek specific membrane surface proteins between ecmhi Fibs and total human membrane surface proteins. **B** Correlation analysis of membrane protein gene expression with the ECM^hi^ Fibs signature score. The analysis was performed on publicly available bulk RNA-seq datasets from human kidney biopsies (GSE66469 and E-MTAB-2502). Genes are ranked by their Pearson correlation coefficients. **C** Violin plot showing the expression levels of selected genes among fibroblast subpopulations. **D** Volcano plot showing up- or downregulated genes of CD248^+^ fibroblasts compared with those in CD248^-^ fibroblasts in the ScRNA-seq data. **E** Representative immunofluorescent staining of specified proteins in fibrotic kidney. EPCAM, a marker for tubular epithelial cells, and COL1A1, a marker for renal interstitium area. Scale bar, 100 μm. Correlation analysis of the COL1A1^+^CD248^+^ area with (**F**) the collagen deposition area and **G** eGFR) in our clinical CKD patient cohort (*n* = 56). Pearson correlation coefficients and *P values* are indicated. **H**, **I** Correlation analysis between *CD248* gene expressed level and core ECM score based on public mRNA-seq datasets (E-MTAB-2502 and GSE66494, respectively). The solid lines represent the linear regression fit, and the shaded areas indicate the 95% confidence interval. Statistical significance was determined using a two-sided Wilcoxon rank-sum test with Bonferroni correction (**D**), and two-sided Pearson correlation analysis (**B**, **F**–**I**). Source data are provided as a Source Data file.

As the terminal pro-fibrotic effector population identified by our trajectory analysis, ECM^hi^ Fibs represent a prime target for anti-fibrotic therapy. To identify targetable cell-surface markers on these cells, we intersected their differentially expressed membrane molecules (DEMMs) (logFC > 0.5, FDR < 0.25) with a comprehensive human membrane proteome database^25^ (Fig. 2A and Supplementary data 5). Correlation analysis showed the top eight membrane biomarkers that were highly co-expressed with the ECM^hi^ Fib signature (Fig. 2B and Supplementary Data 6). A violin plot further showed the expression pattern of these biomarkers among fibroblast subsets (Fig. 2C). Herein, we noticed that CD248, which is significantly upregulated in activated fibroblasts of diverse organ fibrosis diseases, was specifically expressed in ECM^hi^ Fibs. The volcano plot visualizing the differential expression analysis between CD248^+^ and CD248^−^ fibroblasts indicated that compared with other fibroblasts, CD248^+^ fibroblasts exhibited the most significant differences in the expression genes encoding ECM molecules, notably associated with stromal activation and fibrosis progression, such as *POSTN*, *COL1A1,* and *FN1* (Fig. 2D, Supplementary Data 7). By contrast, we found that *ACTA2* (encodingα-SMA), a classic myofibroblast marker, is non-specifically expressed across fibroblast subpopulations (Fig. 2C). This finding aligns with recent studies indicating that α-SMA is also present in pericytes and smooth muscle cells, not exclusively in myofibroblasts^26,27^. Additionally, the results of multiplex immunohistochemical (mIHC) staining demonstrated that in renal tissues of CKD patients (Fig. 2E), CD248 was selectively co-localized with COL1A1 in the non-epithelial stroma. Notably, in healthy control tissues, stromal CD248 expression was barely detectable in the interstitium. Furthermore, CD248 expression correlated significantly with renal collagen deposition (Fig. 2F), and negatively with renal function (Fig. 2G). Similarly, using the mRNA-seq data, we also observed a significant positive correlation between the *CD248* expression level and the core ECM score (Fig. 2H, I).

### Selective tracing and ablation of CD248+ fibroblasts in a genetic mouse model reveal their role in facilitating stromal remodeling

**Fig. 3:**
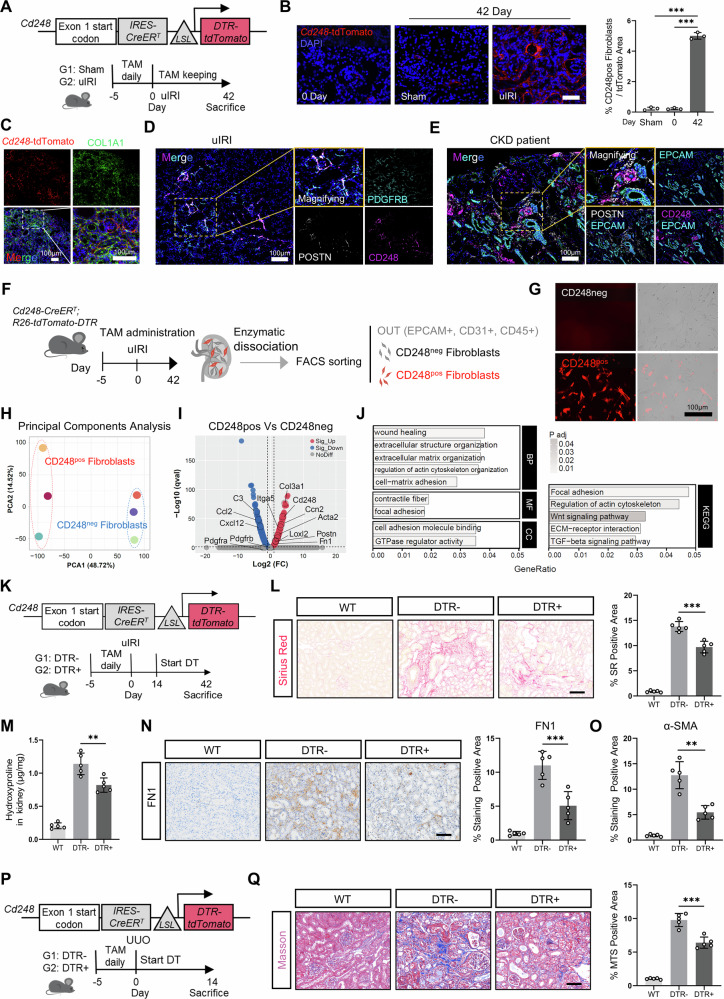
CD248^+^ myofibroblasts serve as an effector subset to promote renal fibrogenesis. **A** Schematic of the genetic (top) and experimental (bottom) approach for the generation of *Cd248*^*CreERT; TdTomato-DTR*^ mice. **B** Representative immunofluorescence images of CD248 and DAPI (*n* = 3). Quantification of CD248^+^ myofibroblasts (tdTomato^+^). Scale bar, 50 μm. **C** Representative immunofluorescence images of specified proteins in mice after 42 days with uIRI injury. Scale bar, 100 μm. **D** Representative images of multi-immunofluorescence of specified proteins in mice after 42 days with uIRI injury. **E** Representative images of multi-immunofluorescence of specified proteins in CKD patient. **F** Schematic of the procedure for sorting CD248^+^ and CD248^-^ fibroblasts using FACS. **G** Representative staining of sorted CD248^+^ and CD248^-^ fibroblasts. **H** PCA plot representative of mRNA-seq analysis of sorted CD248^+^ and CD248^-^ fibroblasts. **I** Volcano plot showing differentially expressed genes of sorted CD248^+^ fibroblasts compared with those in CD248^-^ fibroblasts in the mRNA-seq data. **J** KEGG enrichment analysis of differentially expressed genes between sorted CD248^+^ and CD248^-^ fibroblasts. **K** Schematic illustrating the depletion of CD248+ cells in the uIRI model. Both experimental (*Cd248*^*CreERT; TdTomato-DTR*^) mice and their Cre-negative littermate controls (*Cd248*^*wt/wt;TdTomato-DTR*^) were subjected to uIRI followed by DT administration to ablate DTR-expressing cells. **L** SR staining of kidney tissue after uIRI ± diphtheria toxin (*n* = 5 per group). Scale bar, 50 μm. Quantification of positive area of SR (right). **M** The hydroxyproline content in mouse kidney tissues with uIRI injury (*n* = 5). **N** Representative images of FN immunohistochemical (IHC) staining in uIRI (*n* = 5) mice. **O** Quantification of IHC staining for α-SMA in mouse kidney tissues with uIRI (*n* = 5). **P** Schematic of the experimental approach for the *Cd248*^*CreERT; TdTomato-DTR*^ mice with UUO injury. **Q** Masson staining of kidney tissue after UUO (*n* = 5), and quantification of positive area of Masson staining. **P < 0.01, ***P < 0.001. All data are represented as mean ± s.d. Statistics were calculated using two-tailed, ANOVA with Tukey’s test. Source data are provided as a Source Data file.

To determine the impact of CD248^+^ myofibroblasts on kidney fibrogenesis, a genetic mouse model was generated, and enabled us to trace and selectively deplete CD248-expressing cells following treatment with tamoxifen (TAM) and subsequent administration of diphtheria toxin (DT) (*Cd248*^*CreERT; TdTomato-DTR*^) (Fig. 3A). Utilizing a unilateral kidney ischemia-reperfusion (uIRI) model, we observed that CD248^+^ myofibroblasts were diffusely distributed in the renal interstitium after 42 days of modeling, whereas they were almost undetectable at day 0, or in the sham group (Fig. 3B). The co-localization of TdTomato with fibroblast activation markers COL1A1 and FN further demonstrated that CD248^+^ myofibroblasts represented a subpopulation promoting maladaptive repair (Fig. 3C and Supplementary Fig. 6A). To validate these findings at the protein level, we performed immunofluorescence on fibrotic kidney tissues from both mice and humans, and observed that CD248 exhibited a more restricted expression pattern within sites of active fibrosis (POSTN^+^) compared to the canonical activation marker, PDGFRβ ((Fig. 3D, E). Collectively, this evidence demonstrated that CD248 expression faithfully reflects the activation state of pro-fibrotic fibroblasts during disease progression.

To further identify the function of CD248^+^ fibroblasts, fluorescence-activated cell sorting (FACS) was used to isolate CD248^+^ (TdTomato^+^) primary fibroblasts (CD45^−^CD31^−^EPCAM^−^) from the kidneys of uIRI mice (Fig. 3F, G and Supplementary Fig. 6B). Subsequent bulk mRNA-seq revealed that CD248^+^ fibroblasts were transcriptionally distinct from CD248^-^ cells (Fig. 3H). Specifically, we found ECM-related genes such as *Postn*, *ccn2*, *Acta2* and *Fn1* were upregulated in CD248^+^ fibroblasts, by contrast, inflammatory factors such as *C3*, *Ccl2,* and *Cxcl12* were downregulated, similar with our findings in ScRNA-seq data shown in ECM^hi^ Fibs (Fig. 3I). qRT-PCR validation confirmed that markers of stromal activation were significantly enriched in the CD248^+^ myofibroblast population (Supplementary Fig. 6C). Functional analysis indicated that the transcriptional signature of CD248^+^ myofibroblasts was dominated by pro-fibrotic pathways (Fig. 3J). These findings demonstrated that CD248^+^ fibroblasts represented a subset contributing to excessive ECM deposition in the renal fibrogenic niche.

Given the potential role of CD248^+^ myofibroblasts in renal fibrogenesis, we next assessed the impact of selectively depleting CD248^+^ myofibroblasts on the progression of fibrosis in vivo. Initially, we confirmed the efficiency of CD248 depletion following DT administration (Supplementary Fig. 6D). We evaluated whether the selective depletion of CD248^+^ myofibroblasts could alleviate renal fibrosis in the uIRI model (Fig. 3K). Sirius red staining and hydroxyproline assays revealed a noticeable reduction in stromal collagen following the elimination of CD248^+^ myofibroblasts (Fig. 3L, M). Immunohistochemical (IHC) staining showed a significant decrease in the expression of myofibroblast activation markers, including α-SMA and FN, in the CD248^+^ fibroblasts-depleting group (Fig. 3N, O and Supplementary Fig. 6E). To test the generalizability of these observations, we replicated the depletion experiment in the unilateral ureteral obstruction (UUO) model, another classic model of renal fibrosis (Fig. 3P). Reassuringly, the selective elimination of CD248+ fibroblasts yielded a similar protective phenotype, significantly blocking aberrant matrix accumulation (Fig. 3Q and Supplementary Fig. 6F–H). These results confirmed that CD248^+^ myofibroblasts were the predominant effectors responsible for the fibrotic progression of the kidney, potentially serving as a promising target for fibrosis treatment.

### Specific depletion of CD248 in fibroblasts inhibits myofibroblast activation and reduces fibrotic progression

**Fig. 4:**
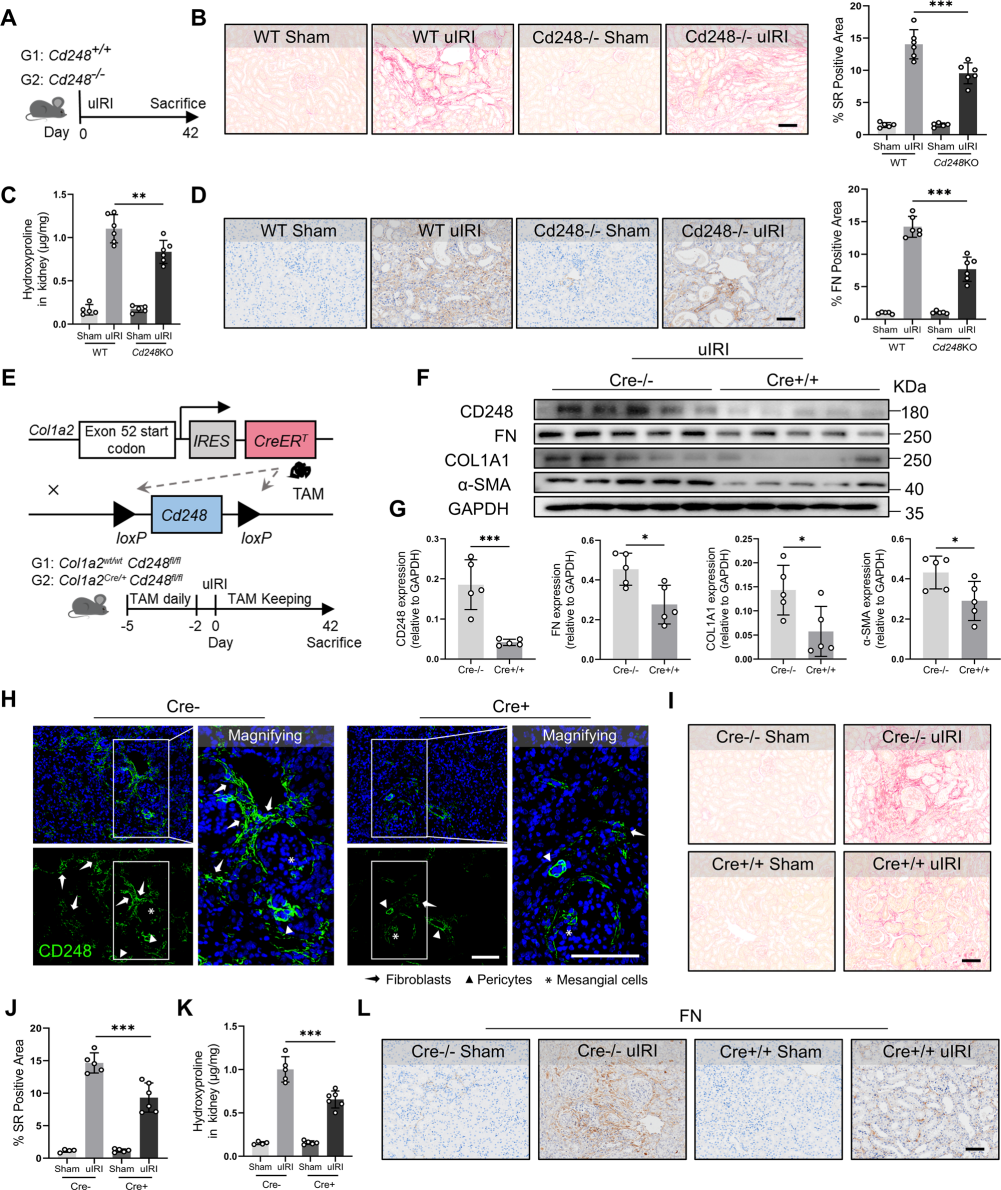
Loss of CD248 suppress myofibroblast activation and renal fibrogenesis. **A** Schematic of experimental process of *Cd248 KO* mice with uIRI. **B** SR staining of kidney tissues after uIRI (*n* = 5 and *n* = 6 biologically independent animals for sham and uIRI groups, respectively) (left), and quantification of positive area. Scale bar, 50 μm. **C** The hydroxyproline content in murine kidney tissues with uIRI (*n* = 5 and *n* = 6 biologically independent animals for sham and uIRI groups, respectively.). **D** Representative images of IHC staining of FN (*n* = 5 and *n* = 6 biologically independent animals for sham and uIRI groups, respectively.) (left), and quantification of positive area (right). Scale bar, 50 μm. **E** Schematic of the genetic (top) and experimental (bottom) approach for the generation of *Col1a2*^*CreERT*^*Cd248*^*fl/fl*^ mice with uIRI injury. Western blot analysis of specified protein expression levels in Cre^−/−^ and Cre^+/+^ murine kidneys 42 days post-uIRI injury (*n* = 5) (**F**), and expression quantification, normalized to GAPDH (**G**). **H** Representative immunofluorescence images of CD248 in murine kidneys 42 days post-uIRI injury. Arrows point to interstitial fibroblasts, arrowheads to pericytes, and asterisks to mesangial cells. **I**, **J** SR staining of kidney tissues after uIRI (**I**), and quantification of positive area. *n* = 3 (Cre^-^ sham), 5 (Cre^-^ uIRI), 4 (Cre^+^ sham), and 6 (Cre^+^ uIRI) biologically independent animals. **J**. Scale bar, 50 μm. **K** The hydroxyproline content in murine kidney tissues with uIRI. *n* = 3 (Cre^-^ sham), 5 (Cre^-^ uIRI), 4 (Cre^+^ sham), and 6 (Cre^+^ uIRI) biologically independent animals. **L** Representative images of IHC staining of FN. *n* = 3 (Cre^-^ sham), 5 (Cre^-^ uIRI), 4 (Cre^+^ sham), and 6 (Cre^+^ uIRI) biologically independent animals. (left), and quantification of positive area (right). Scale bar, 50 μm. *P < 0.05, **P < 0.01, ***P < 0.001. All data are represented as mean ± s.d from *n* ≥ 3 independent experiments. Statistics were calculated using two-tailed, unpaired Student’s *t* test (**G**) or ANOVA with Tukey’s test (**B**–**D**, **J**, **K**). Source data are provided as a Source Data file.

We next examined whether CD248 directly modulates the activation of myofibroblasts to progress renal fibrosis in vivo by establishing *Cd248* knockout *(Cd248*^−/−^*)* mice. The results showed that after CD248 depletion, injured kidneys of uIRI model mice exhibited reduced collagen deposition and a decreased hydroxyproline content (Fig. 4A–C). Furthermore, immunohistochemical staining revealed decreased expression of FN in the stroma (Fig. 4D). As expected, the same phenotype was observed in UUO model mice (Supplementary Fig. 7A–F).

While our studies using both the uIRI and UUO models further strengthened the evidence for the anti-fibrotic effects of global *Cd248* deletion^28^, the key outstanding question was whether this anti-fibrotic effect is primarily driven by its deletion in fibroblasts. To address this directly, we employed the well-established pan-fibroblast *Col1a2-Cre* driver^29^ to specifically delete *Cd248* within the fibroblast lineage. This allowed us to precisely assess the cell-autonomous contribution of fibroblast-expressed CD248 to their phenotypic state and pro-fibrotic function in vivo. In the context of uIRI injury, our findings showed a reduction in the level of CD248 protein in the kidneys of TAM-treated *Col1a2*^*CreERT*^*Cd248*^*fl/fl*^ mice, accompanied by decreased α-SMA, COL1A1, and FN levels (Fig. 4E–G). Immunofluorescence staining showed that TAM-induced CD248 knockout was restricted in fibroblasts, but not pericytes or mesangial cells (Fig. 4H). Moreover, conditional depletion of CD248 resulted in reduced fibrosis development and decreased FN expression in the renal stroma compared with those in the *Col1a2*^*wt/wt*^ control group (Fig. 4I–L). To further confirm our results, UUO model *Col1a2*^*CreERT*^*Cd248*^*fl/fl*^ mice were used to assess the impact of CD248 deficiency in fibroblasts during fibrotic progression (Supplementary Fig 7G). Consistent with our hypothesis, fibroblast-specific deletion of *Cd248* in TAM-administered *Col1a2*^*CreERT*^*Cd248*^*fl/fl*^ mice resulted in a marked reduction in renal fibrosis. This amelioration was quantitatively confirmed by a significant decrease in collagen deposition, as measured by both Sirius red staining and hydroxyproline assays (Supplementary Fig. 7H, I). Together, our data demonstrated that CD248, specifically expressed by pro-fibrotic myofibroblasts, is a crucial driving factor of myofibroblast activation and fibrosis progression.

### CD248 remodels the physical microenvironment of the kidney through mechanotransduction-mediated YAP nuclear translocation

**Fig. 5:**
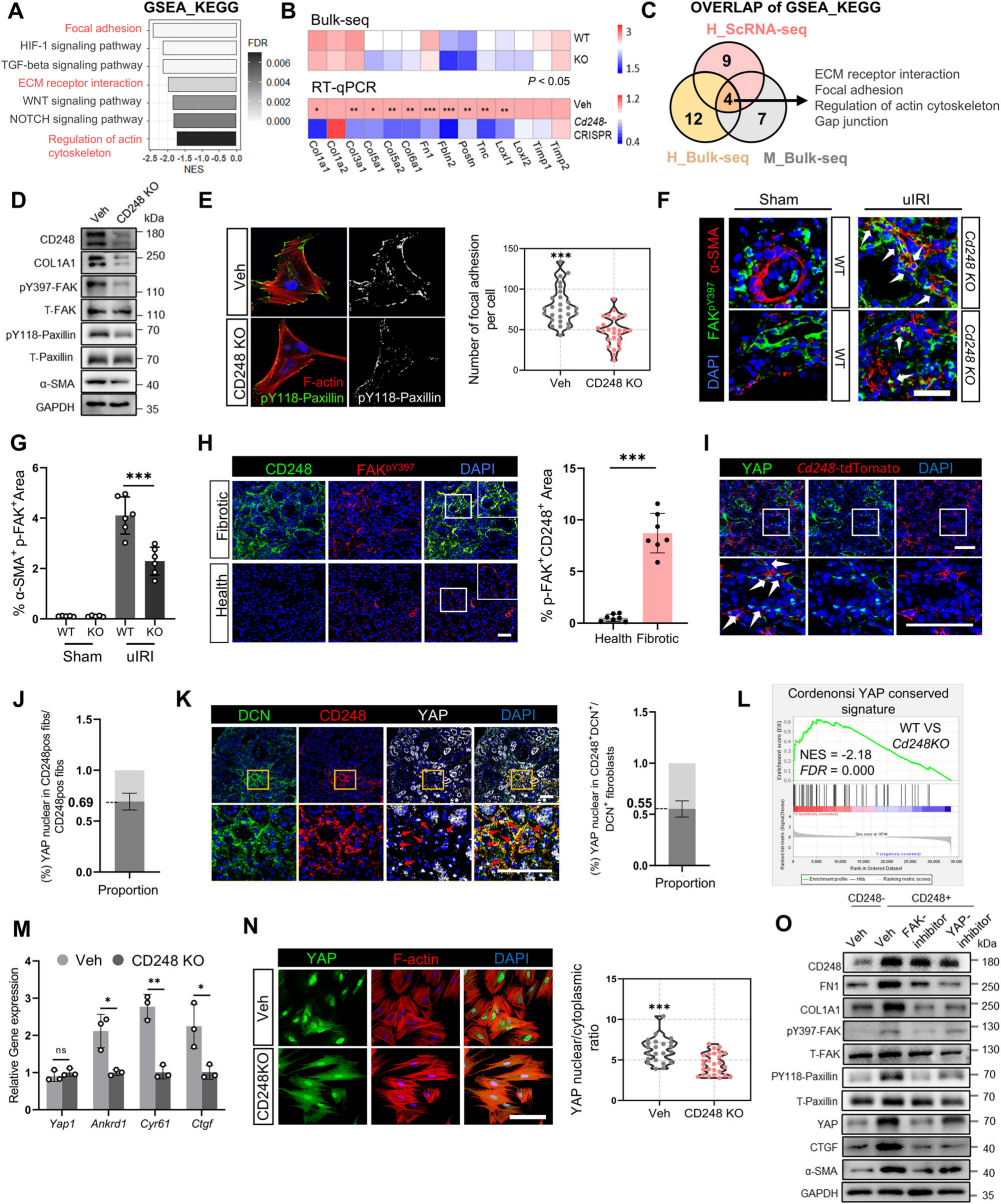
CD248 modulates myofibroblast activation via focal adhesion-YAP mediated mechanotransduction. **A** GSEA pathways analysis in UUO kidneys. **B** Selected genes expression was compared by mRNA-seq (above). Selected genes expression was compared by RT-qPCR (below). *n* = 3 biological replicates each group. **C** Overlap analysis was performed to identify intersecting signaling pathway among ScRNA-seq, human mRNA-seq, and murine mRNA-seq. **D** Western blot analysis of the *Cd248* KO efficiency by specified proteins. **E** Immunofluorescence analysis of pY118-paxillin in fibroblasts. Scale bar, 50 μm (left), and focal adhesion numbers per cell (right). *n* = 27 (Vehicle) and *n* = 26 (CD248KO). **F**, **G** Representative immunofluorescence images of pY397-FAK and α-SMA in *Cd248*^*wt/wt*^ and *Cd248*^−/−^ mice (*n* = 5, and 6 biologically independent animals for sham and uIRI groups, respectively). Scale bar, 100 μm (**F**). Quantification of positive area of pY397-FAK^+^ myofibroblasts (**G**). **H** Representative immunofluorescent staining of pY397-FAK and CD248 in kidneys from the health (*n* = 7) and CKD patients (*n* = 7). Scale bar, 100 μm. Quantification of CD248^+^ pY397-FAK^+^ myofibroblasts. **I** Representative immunofluorescent staining of YAP and *Cd248*^tdTomato^ after uIRI injury. Scale bar, 100 μm. **J** Quantification of the number of *Cd248*^+^ myofibroblasts with YAP nuclear translocation. **K** Representative immunofluorescent staining of YAP and CD248 in human fibrotic kidney. Scale bar, 100 μm. Quantification of the number of CD248^+^ myofibroblasts with YAP nuclear translocation. *n* = 50 independent fields of view. **L** Gene set enrichment analysis of YAP specific signature. *n* = 50 independent fields of view. **M** RT-qPCR for YAP target genes after CD248KO (*n* = 3 technical replicates per group). **N** Immunofluorescence analysis of YAP in fibroblasts. Scale bar, 50 μm (left), and YAP nuclear/cytoplasmic ratio (right). *n* = 22 (Vehicle) and *n* = 26 (*Cd248 KO*) field of views. **O** Western blot indicating protein level of myofibroblast. *P < 0.05, **P < 0.01. All data are represented as mean ± s.d from *n* ≥ 3 independent experiments. Statistics were calculated using two-tailed, unpaired Student’s *t* test (**E**, **H**, **M**, **N**) or ANOVA with Tukey’s test (**G**). Source data are provided as a Source Data file.

To further investigate the molecular events in the kidney fibrogenic niche following CD248 deletion, we performed bulk mRNA-seq on whole kidney tissue of CD248^−/−^ mice with fibrosis. GSEA revealed that CD248 deficiency suppressed classical pro-fibrotic signaling pathways (Fig. 5A). Mirroring our data from bulk renal tissues, CRISPR-mediated CD248 knockout in primary fibroblasts also downregulated canonical pro-fibrotic genes such as *Col1a1, Postn* and *Fn1* (Fig. 5B). We then conducted overlap analysis to identify enriched pathways using bulk mRNA-seq and ScRNA-seq data from patients with CKD, and bulk mRNA-seq data from UUO mice. The top overlap pathways were related to cell response to ECM and the activation of biomechanical signaling (Fig. 5C). Intriguingly, the CD248-enriched pathways aligned with those driving fibroblast differentiation into ECM^hi^ Fibs, suggesting a key role for CD248 in mechanotransduction-mediated ECM^hi^ Fibs activation (we continue to use the term ‘myofibroblasts’ following, which is widely accepted in mouse models) (Figs. 1P, 5C, and Supplementary Data 8).

Focal adhesion assembly serves as critical hubs for transducing mechanical signals from disrupted ECM to downstream cellular mechanical signals. Indeed, we found that the focal adhesion pathway mediates the activation of CD248^+^ myofibroblasts (Supplementary Fig. 8A). CD248-deficient fibroblasts showed the decreased formation of focal adhesions (Fig. 5D, E). In vivo analysis of mice and human fibrotic kidneys also revealed a significant overlap of CD248 and pY397-FAK expression (Fig. 5F–H and Supplementary Fig. 8B, C). To further elucidate the role of CD248 in fibroblast mechanotransduction, we assessed the response of *Cd248*-deficient fibroblasts to substrate stiffness. We found that these cells exhibited an impaired response to rigid substrates, as evidenced by diminished focal adhesion formation and suppressed cytoskeletal remodeling, indicating a key role of mechanosignal in CD248-driven myofibroblasts transition (Supplementary Fig. 8D, E). To solidify the link between CD248 and mechanosignaling in vivo, we performed immunofluorescence on both murine (*Cd248* tracer) and human fibrotic kidneys. This revealed a co-localization of nuclear YAP, the core effector of mechanotransduction, with CD248^+^ myofibroblasts, which strongly suggested that CD248 governs fibroblast activation by modulating YAP activity (Fig. 5I–K). Indeed, genetic depletion of *Cd248* resulted in a significant suppression of YAP signaling (Fig. 5L–N, and Supplementary Fig. 8F). Corroborating this, the pro-fibrotic phenotype of CD248^+^ myofibroblasts, characterized by activated mechanical signaling and excessive ECM production, was reversed by pharmacological inhibition of either FAK or YAP (Fig. 5O).

**Fig. 6:**
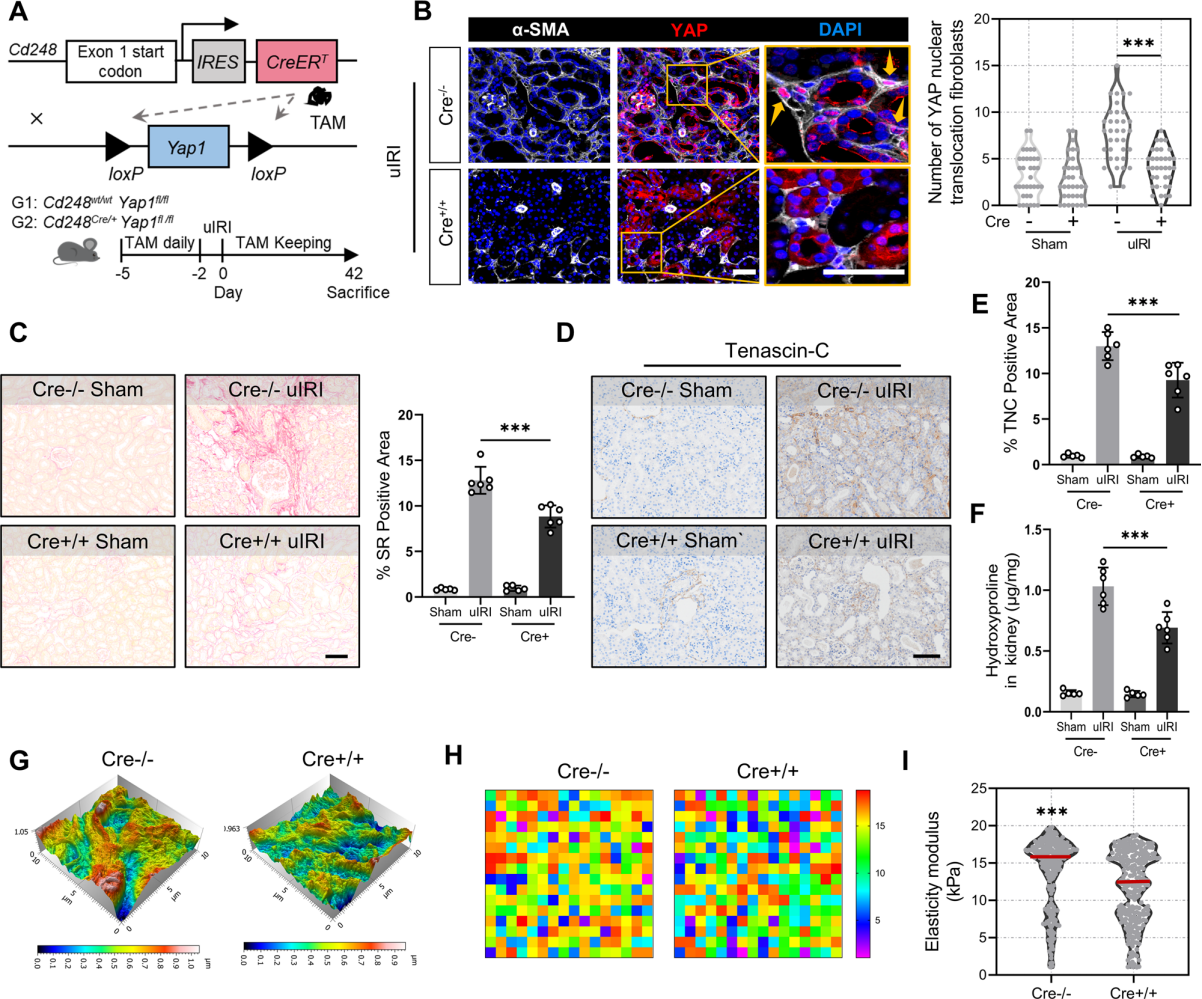
Loss of YAP in CD248^+^ myofibroblasts decreased ECM stiffness and fibrosis. **A** Schematic of the genetic (top) and experimental (bottom) approach for the generation of *Cd248*^*CreERT*^*Yap1*^*fl/fl*^ mice. **B** Immunofluorescence analysis of α-SMA and YAP in kidney tissues of genetic mice after 42 days with uIRI injury (*n* = 5 to 6). Scale bar, 50 μm. Quantification of numbers of YAP nuclear translocation in α-SMA^+^ myofibroblasts under field of views (*n* = 35 field of views per group). **C** SR staining of kidney tissues after uIRI, and quantification of positive area of SR (*n* = 5 and *n* = 6 biologically independent animals for sham and uIRI groups, respectively). Scale bar, 50 μm. Representative images of IHC staining of TNC (*n* = 5 and *n* = 6 biologically independent animals for sham and uIRI groups, respectively) (**D**), and quantification of positive area of TNC (**E**). Scale bar, 50 μm. **F** The hydroxyproline content in murine kidney tissues with uIRI (*n* = 5 and *n* = 6 biologically independent animals for sham and uIRI groups, respectively). **G** Atomic force (AFM) scanning three-dimensional reconstruction map of stromal matrix for kidney tissues of uIRI mice. **H**, **I** Representative images of stiffness map of the renal matrix measured by AFM (**H**). Quantification of stromal matrix stiffness (**I**). ***P < 0.001. All data are represented as mean ± s.d from *n* ≥ 3 independent experiments. Statistics were calculated using two-tailed, unpaired Student’s *t* test (**I**) or ANOVA with Tukey’s test (**B**, **C**, **E**, F). Source data are provided as a Source Data file.

To determine the regulatory role of YAP in CD248^+^ myofibroblasts, we generated transgenic mice with conditional knockout of *Yap1* specifically in CD248^+^ myofibroblasts, *Cd248*^*CreERT*^*Yap1*^*fl/fl*^ ^30^ (Fig. 6A). Our results firstly verified a significant reduction of the YAP expression within myofibroblasts of the TAM-treated *Cd248*^*CreERT*^*Yap1*^*fl/fl*^ group (Fig. 6B and Supplementary Fig. 9A, B). Selective YAP depletion in CD248^+^ myofibroblasts effectively reduced stromal collagen deposition within uIRI and UUO models (Fig. 6C, F and Supplementary Fig. 9C, E). IHC staining of tenascin-C (TNC), an indicator of active mechanical cues in tissues fibrosis^31,32^, which is regulated by CD248, showed a reduced expression in *Cd248*^*CreE*RT/+^ mice (Fig. 6D, E). Utilizing AFM to reconstruct the 3D morphology of the renal interstitium, we observed the thickness of the matrix deposited in *Cd248*^*CreE*RT/+^ group (Fig. 6G). Upon further calculation of the elastic modulus of fresh renal tissue using AFM, we found that the stiffness of the renal stroma in *Cd248*^*CreE*RT/+^ mice was significantly lower (Fig. 6H, I). We further investigated whether a similar phenotype could be observed in fibroblasts with CD248 knockout. As expected, our results demonstrated thinner matrix deposition and reduced collagen fiber crosslinking in the *Col1a2*^*CreERT*^*Cd248*^*fl/fl*^ mice (Supplementary Fig. 9F, H). Taken together, our results indicated that CD248 plays a crucial role in mediating mechanical signaling-dependent YAP activation, as well as in the remodeling of the physical microenvironment within the fibrotic kidney.

### CD248 mediates mechanotransduction by recruiting IQGAP1-ARF6·GTP via interaction with aberrant ECM

**Fig. 7:**
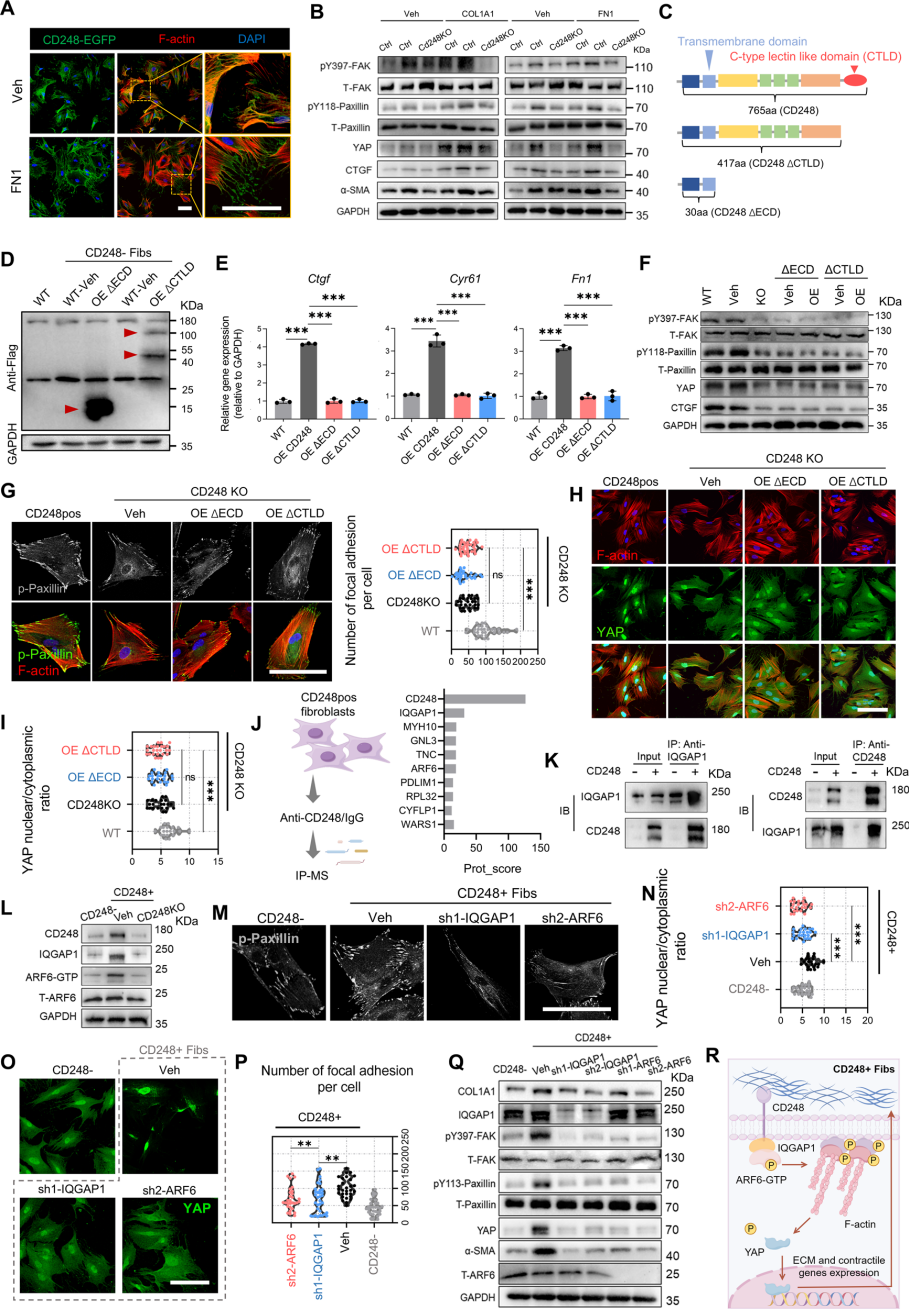
IQGAP1-ARF6·GTP activation via CD248 CTLD in response to aberrant ECM deposition sustains mechanical feedback loop. **A** Immunofluorescence analysis of CD248-EGFP in myofibroblasts. **B** Western blot indicating protein level of fibroblasts cultured on FN- or COL1A1-coated dishes. **C** Exogenous ΔECD or ΔCLTD CD248 fragments. **D** Western blot analysis showing expression of exogenous CD248 fragments. **E** qRT-PCR showing mRNA levels in primary fibroblasts. *n* = 3 technical duplications. **F** Western blot showing the expression level of specified proteins of fibroblasts. **G** Immunofluorescence analysis of pY118-paxillin, and focal adhesion numbers per cell. Wild type (*n* = 31), CD248 KO (*n* = 29), CD248 KO with ΔECD (*n* = 31), CD248 KO with ΔCLTD (*n* = 34). **H**, **I** Immunofluorescence analysis of YAP nuclear/cytoplasmic ratio. Wild type (*n* = 20), CD248 KO (*n* = 22), CD248 KO (*n* = 22), CD248 KO with ΔECD (*n* = 22), CD248 KO with ΔCLTD (*n* = 22). **J** The protein scores of the top proteins identified by MS are shown from CD248^+^ fibroblasts. **K** Co-IP analysis of the interaction between CD248 and IQGAP1 in CD248^+^ versus CD248^-^ fibroblast lysates. **L** The specified proteins were analyzed by Western blotting. **M**, **N** Immunofluorescence analysis of pY118-paxillin. CD248^-^ fibroblasts (*n* = 30), CD248^+^ with vehicle (*n* = 29), sh-IQGAP1 (*n* = 33), sh-ARF6 (*n* = 30). **O**, **P** Immunofluorescence analysis of YAP nuclear translocation, CD248^-^ fibroblasts (*n* = 30), CD248^+^ with vehicle (*n* = 29), sh-IQGAP1 (*n* = 33), sh-ARF6 (*n* = 30). **Q** The specified proteins were analyzed by Western blotting. **R** Schematic illustrating the proposed positive feedback loop driving fibrogenesis. Upregulation of CD248 on fibroblasts promotes IQGAP1/ARF6-YAP mechanosignaling, leading to ECM deposition and fibroblast activation. The resulting matrix stiffening, in turn, further enhances CD248-mediated signaling, creating a self-perpetuating fibrotic loop. **P < 0.01, ***P < 0.001. Scale bar, 50 μm. All data are represented as mean ± s.d from *n* ≥ 3 independent experiments. Statistical significance was determined using one-way ANOVA followed by two-sided Tukey’s multiple comparisons test (**E**, **G**, **I**, **N**, **P**). Source data are provided as a Source Data file.

Recent studies have shown that dysregulated ECM properties are important mechanical cues for myofibroblast activation^13^. We next hypothesized that CD248 acts as a mechanosensor to recognize changes in the ECM. To test this hypothesis, we constructed an exogenous CD248-EGFP fusion protein in primary renal fibroblasts and observed that CD248 tended to localize on the membrane protrusions of pseudopodia in response to fibronectin (Fig. 7A). Furthermore, we found that in ECM-containing media, CD248^+^ myofibroblasts showed significant activation of mechanical signaling and ECM expression, which were inhibited by CD248 knockout (Fig. 7B). To dissect how CD248 bridges aberrant ECM to fibroblast activation, we generated two deletion mutants: one lacking the extracellular domain (CD248-ΔECD) and another specifically missing the C-type lectin-like domain (CD248-ΔCTLD), which is critical for its interaction with the ECM^33^. (Fig. 7C). Following confirmation of successful construct expression by Western blot (Fig. 7D), RT-qPCR was conducted to find that the mRNA levels of *Ctgf*, *Cyr61*, and *Fn1* were specifically elevated in the CD248 overexpressing group, but not in the other groups (Fig. 7E). Moreover, overexpression of exogenous CD248 ΔECD and CD248 ΔCTLD fails to restore the response of fibroblasts to ECM, including mechanical activation, focal adhesion formation and YAP nuclear translocation (Fig. 7F–I). This finding underscores the indispensable role of the CTLD in mediating the mechanosensory feedback loop that drives myofibroblast activation.

We next query to the molecular events involved in the mechanotransduction activation following the interaction of CD248 with aberrant ECM. Immunoprecipitation mass spectrometry (IP-MS) was conducted on CD248-positive myofibroblasts. We then identified a significant enrichment of IQGAP1, a scaffold protein associated with cell adhesion and cytoskeletal rearrangement^34^ (Fig. 7J and Supplementary data 9). The other top molecules identified were cytoskeleton proteins. Subsequent co-immunoprecipitation (Co-IP) experiments confirmed the interaction between CD248 and IQGAP1 (Fig. 7K). Previous studies have demonstrated that the GTPase-activating domain of IQGAP1 can activate ARF6·GTP^35^, a small GTPase involved in focal adhesion assembly and mechanotransduction^36^. We hypothesize that CD248 may mediate downstream mechanical signaling by recruiting ARF6·GTP through IQGAP1. Western blot analysis confirmed increased levels of IQGAP1 and ARF6·GTP in CD248^+^ myofibroblasts (Fig. 7L), which were reduced when CD248 was knocked out. Immunofluorescence staining showed the decreased focal adhesion assembly and YAP nuclear translocation of fibroblasts in response to ECM (Fig. 7M–P). More importantly, silencing IQGAP1 and ARF6 reduced mechanical cues, and suppressed the activation of myofibroblasts (Fig. 7Q). In summary, our results indicated that CD248 converts disordered ECM deposition from the microenvironment into mechanotransduction to activate fibroblasts through the IQGAP1-ARF6·GTP pathway (Fig. 7R).

### Targeted CD248 monoclonal antibody IgG78 effectively suppresses myofibroblast activation and kidney fibrogenesis in vitro and in vivo

**Fig. 8:**
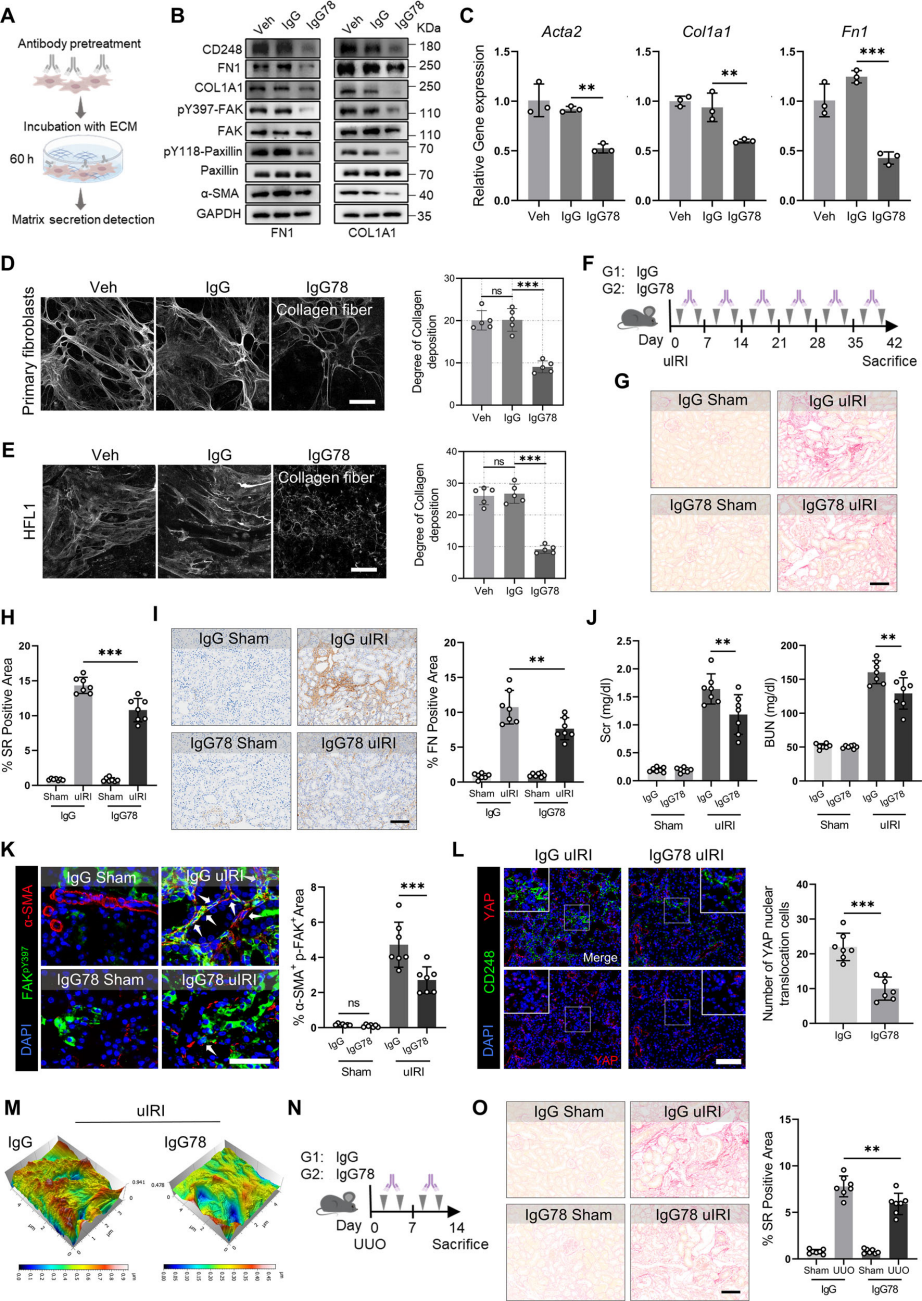
Monoclonal antibody IgG78 administration effectively attenuates ECM deposition in vitro and in vivo. **A** Schematic of the experimental approach for IgG78 blocking myofibroblast activation in vitro. **B** Western blot showing the activation levels of focal adhesion-YAP signaling in myofibroblasts treated with IgG78, cultured on FN- or COL1A1-coated dishes. **C** RT-qPCR showing mRNA level in myofibroblasts treated with IgG78, cultured on FN coated dishes. *n* = 3 technical duplications. Second harmonic generation (SHG) imaging showing the collagen deposition derived from primary fibroblasts of mice (**D**, *n* = 5 technical duplications), or HFL1 (**E**, *n* = 5 technical duplications), treated with IgG or IgG78. Scale bar, 50 μm. **F** Schematic of the experimental approach for uIRI mice with IgG78 treatment. **G**, **H** Representative images of SR staining of uIRI kidneys, and quantification of positive area (*n* = 7 mice). Scale bar, 50 μm. **I** Representative images of FN staining of uIRI kidneys, and quantification of positive area (*n* = 7 mice). Scale bar, 50 μm. **J** Kidney injury with uIRI suggested by serum creatinine and urea nitrogen level (*n* = 7 mice). **K** Representative immunofluorescence images of pY397-FAK and α-SMA in mice with uIRI injury. Quantification of positive area of α-SMA^+^pY397-FAK^+^ myofibroblasts (*n* = 7 mice). Scale bar, 100 μm. **L** Representative immunofluorescence images of CD248 and YAP in mice with uIRI injury. Quantification of CD248^+^ myofibroblasts with YAP nuclear translocation (*n* = 7 mice). Scale bar, 100 μm. **M** AFM scanning reconstruction map of stromal matrix for kidney of uIRI mice. **N** Schematic of the experimental approach for UUO mice with IgG78 treatment. **O** SR staining of kidney tissues after UUO, and quantification of positive area (*n* = 7 mice). Scale bar, 50 μm. **P < 0.01, ***P < 0.001 using one-way ANOVA followed by two-sided Tukey’s multiple comparisons test (**C**–**E**, **H**–**K**, **O**), and two-tailed, unpaired Student’s *t* test (**L**). All data are represented as mean ± s.d from *n* ≥ 3 independent experiments. Source data are provided as a Source Data file.

Given that our results demonstrated the pivotal role of CD248 in linking the ECM and myofibroblast activation, we hypothesized that restricting ECM accessibility to CD248 could potentially disrupt the mechanical feedback loop involved in fibrotic progression. Previously, we generated a fully human antibody, IgG78, based on a CD248-specific single-chain variable fragment (scFv78), and confirmed its excellent bioactivity both in vitro and in vivo^37,38^. To validate that IgG78 could effectively block the ECM-CD248 interaction and inhibit the fibrotic feedback loop, primary fibroblasts were pre-treated with IgG78 and incubated in ECM-enriched medium. Mechanical signaling activation and ECM expression were subsequently assessed (Fig. 8A). Activation of the FAK-Paxillin-YAP signaling axis and production of ECM proteins, including COL1A1, α-SMA, FN, and CTGF, was decreased in the IgG78 pre-treated fibroblasts (Fig. 8B). RT-qPCR assays demonstrated that IgG78 treatment suppressed ECM production at the mRNA level (Fig. 8C). To directly assess the anti-fibrotic potential of IgG78, we leveraged its cross-reactivity to test effects on both murine and human fibroblasts. IgG78 treatment significantly suppressed collagen production in primary murine fibroblasts and, importantly, in the human fibroblast cell line HFL1, highlighting its therapeutic promise. We found that CD248 inhibition by IgG78 significantly reduced collagen deposition (Fig. 8D, E). These results demonstrated that administration of IgG78 effectively suppressed ECM production by myofibroblasts, suggesting its potential in mechanomedicine in vivo.

**Fig. 9:**
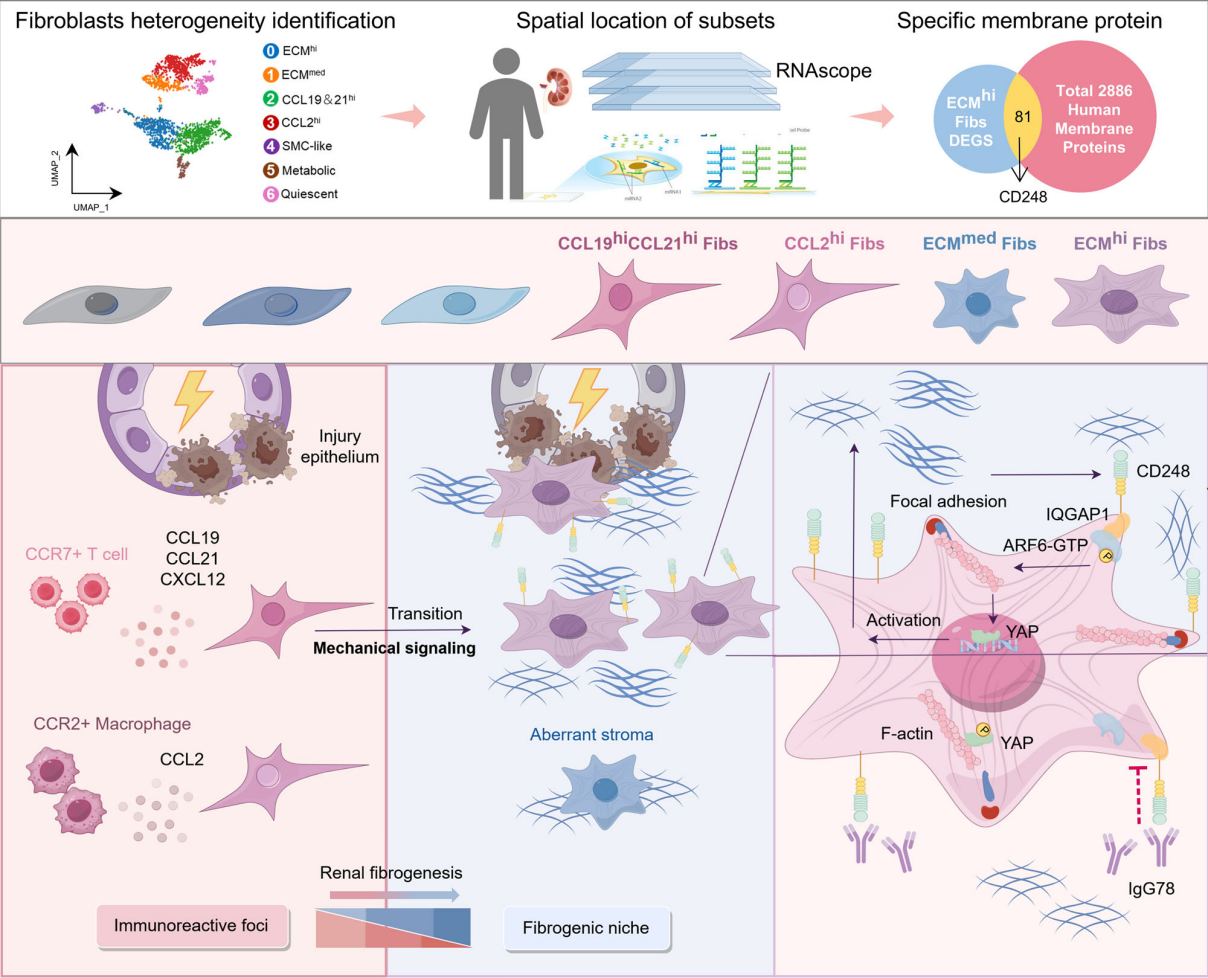
Schematic illustration of the CD248-driven mechanotransduction loop in renal fibrosis. We reveal a distinct, spatially-resolved fibroblast heterogeneity in the fibrotic kidney, identifying CD248 as a marker of the ECM-producing myofibroblast niche. During fibrosis progression, the stromal microenvironment transitions from early inflammatory to mature fibrotic phenotypes. In the stiffened matrix, CD248 acts as a key mechanosensor by recruiting IQGAP1 and activating ARF6-GTP, which promotes focal adhesion assembly and nuclear YAP translocation. Targeting this CD248-dependent feedback loop with the specific antibody IgG78 effectively disrupts mechanotransduction and ameliorates renal fibrosis.

We subsequently aimed to investigate the therapeutic potential of IgG78 and assessed its effects on preclinical models of kidney fibrosis. Following induction of uIRI injury in mice (Fig. 8F), our results demonstrated that IgG78 treatment could reduce collagen deposition and FN expression in the renal interstitium (Fig. 8G–I and Supplementary Fig. 10A), and concurrently lowered serum levels of Scr and BUN, key indicators of renal function (Fig. 8J). We next explored the effects of IgG78 on mechanotransduction-mediated fibroblast activation in vivo. Indeed, immunofluorescence staining revealed that treatment targeting CD248 suppressed stromal activation, as evidenced by decreased levels of α-SMA and FAK-pY397 (Fig. 8K and Supplementary Fig. 10B). Moreover, IgG78 administration also led to reduced CD248 expression and nuclear translocation of YAP within CD248^+^ myofibroblasts (Fig. 8L and Supplementary Fig. 10C). Morphological reconstruction by AFM revealed a marked reduction in collagen fiber cross-linking and thickness after IgG78 treatment (Fig. 8M). We further confirmed the efficacy of IgG78 in alleviating ECM production using the UUO model (Fig. 8N, O, and Supplementary Fig. 10D, M). These findings highlighted the notion that therapeutic strategies targeting CD248 could ameliorate renal fibrosis by blocking the mechanotransduction of myofibroblasts. Finally, to assess the safety profile of IgG78, we evaluated its potential hepatotoxicity and systemic toxicity. Given that therapeutic antibodies are primarily metabolized by the liver, we measured serum levels of alanine aminotransferase (ALT) and aspartate aminotransferase (AST) in mice from both fibrosis models. No significant differences were observed between IgG78-treated and control groups (Supplementary Fig. 11A, B). Furthermore, histological examination of major organs at the end of the treatment cycle revealed no evident tissue damage or pathological changes (Supplementary Fig. 11C, D). Collectively, these data demonstrated that IgG78 exhibits a promising biosafety in preclinical settings (Fig. 9).

## Discussion

Efforts to inhibit fibroblast differentiation by targeting activation signals have been explored for many years. Disruption of local biomechanical properties caused by stromal remodeling serves as a critical signal for myofibroblast activation. Yet, selectively targeting the culpable fibrogenic subset while sparing resident, homeostatic subsets has proven elusive. In this study, we identified a key fibroblast subpopulation involved in stromal remodeling that is marked by the membrane protein CD248. Our findings suggested that CD248 serves as a sensor of abnormal ECM properties, activating mechanotransduction to sustain fibroblast activation and promote further remodeling. Notably, we observed an anti-fibrotic effect with IgG78, a monoclonal antibody targeting CD248, suggesting a promising strategy for tissues fibrogenesis.

A recent high-resolution spatial atlas of CKD has revealed distinct microenvironment subtypes of renal stromal areas, often presenting as “mosaic” distribution dominated by fibrogenic microenvironment (FME) and immune microenvironment (IME). The former is characterized by matrix activation markers like POSTN, while the latter is enriched with lymphocytes, as well as chemokines such as CCL19, CCL21, CXCL12 and their receptors^10^. In this study, we provided a critical cellular dimension to the establishment of this spatial map. Through ScRNA-seq analysis and multiplex RNAscope, we identified a previously uncharacterized fibroblast subset, termed CCL19&21^hi^-Fibs. The strong spatial correlation of these cells with CCR7+ lymphocytes lead us to hypothesize that they are the key architects of the IME, a defining feature of which is the presence of these immune cells. Besides, our data revealed that ECM-Fibs act as the architects of the FME by remodeling stromal biomechanical properties. Interestingly, we found that the distinct fibroblast populations were spatially segregated, occupying mutually exclusive territories that correspond to either the IME or FME. Collectively, our data established fibroblast heterogeneity as a non-negligible mechanism driving niche pathological heterogeneity. This positions fibroblasts not as passive cells within the kidney, but as active architects of their pathological microenvironment.

The progression of renal fibrosis is characterized by a pathological transition from inflammation to diffuse scarring. Based on the crucial role of fibroblasts in shaping the local foci, we further found a corresponding phenotypic switch in fibroblasts from i-Fibs to ECM-Fibs. Notably, this differentiation was driven not only by canonical pathways like TGF-β and Wnt but, more strikingly, by mechanotransduction signals. This revelation, however, presents a strategic dilemma: instead of attempting to inhibit a complex web of upstream differentiation signals, a more direct and potent strategy might be to specifically target the resultant pro-fibrotic cell population itself. Therefore, we first defined ECM^hi^ Fibs as the primary contributors to fibrogenesis, based on their high expression of stromal activation genes^20^, such as *POSTN*, *FN1*, and *COL1A1*, rather than the less specific α-SMA marker. Moreover, we identified CD248 as a specific cell-surface membrane protein for ECM^hi^ Fibs. CD248 is expressed not only in fibroblasts but also in stromal cells like pericytes and mesangial cells, yet its exact localization during fibrotic progression is unclear. Here, a *Cd248*^*CreERT; tdTomato-DTR*^ mouse model was used to map CD248 expression in COL1A1 and FN-expressing myofibroblasts during fibrogenesis. Targeted ablation of CD248^+^ myofibroblasts reduces ECM deposition and fibrosis progression, with *Cd248 KO* and *Col1a2*^*CreERT*^*Cd248*^*fl/fl*^ models confirming the role of CD248 in promoting myofibroblast activation, thereby promoting renal fibrogenesis.

The phospho-FAK/Paxillin signaling pathway, critical in focal adhesion-mediated mechanotransduction^39^, promotes YAP nuclear translocation^40^, thereby activating myofibroblasts and increasing stromal stiffness^15^. Our findings showed that CD248^+^ myofibroblasts exhibit elevated activation of the FAK/Paxillin-YAP pathway and higher ECM production than other fibroblasts, while CD248 knockout disrupts the myofibroblastic phenotype. In vivo, YAP deficiency in CD248^+^ myofibroblasts reduced stromal hyperplasia and ECM stiffness in renal fibrosis models. Although CD248 mediates cellular adhesion to the ECM, its role in cooperating with abnormal ECM to trigger downstream mechanotransduction remains unclear. Our data indicated that the CD248-driven FAK/Paxillin-YAP pathway is sensitive to ECM binding, suggesting that CD248 acts as a mechanosensory receptor for ECM changes in the fibrogenic niche. Notably, expression of truncated CD248 variants, either ΔCTLD, which interacts with the ECM^33^, or ΔECD, failed to rescue CD248-mediated mechanotransduction activation, highlighting the functional importance of full-length CD248 in this pathway.

During fibrotic progression, CD248 expressed by fibroblasts has been shown to act as a co-receptor with cytokine receptors such as TGFβR2 or PDGFRα^41,42^. Our findings indicated that CD248 could sense abnormal ECM deposition, sustaining a biomechanical signaling feedback loop. However, the specific molecular events by which CD248 mediates mechanotransduction activation remain unclear. Members of the Ras superfamily of small GTPases are critical regulators of cytoskeletal dynamics and cell migration, with ARF6 playing a significant role in the activation of mechanotransduction^36^. Nevertheless, direct evidence clarifying ARF6 as a mediator of fibroblast activation during fibrosis progression is still lacking. Our data revealed that CD248 recruits the active GTP-bound form of ARF6 via the scaffold protein IQGAP1, thereby promoting downstream FAK/Paxillin-YAP signaling, myofibroblast activation, and stromal remodeling. However, we acknowledge that while our data support this biochemical interaction, establishing a definitive causal hierarchy between CD248 and the IQGAP1-ARF6 axis would benefit from future validation using genetic epistasis models, and that another limitation in our proposed model is that there is currently no direct evidence demonstrating that matrix stiffness directly regulates CD248 expression levels.

Despite their potential, effective mechanomedicine based on monoclonal antibodies (mAbs) has not yet been approved for anti-fibrotic treatment. Current approaches mainly target TGFβ or Rho pathways; however, their widespread expression and essential roles in tissue homeostasis limit their clinical applications^19,43^. Besides, there is still a paucity of assessment on the inhibitory effects of CD248-targeting mAbs on fibrosis^44^. In this study, we demonstrated that IgG78 represents a promising candidate for therapeutic interventions targeting ECM stiffening and renal fibrosis. Although biological systems possess redundancy, the profound anti-fibrotic effects observed upon either genetic ablation or antibody-mediated blockade of CD248 suggest its role is both dominant and non-redundant in the context of the fibrotic kidney. Mechanistically, we speculate that IgG78 functions by physically blocking the interaction between CD248 and the disordered ECM, potentially targeting the CTLD domain. However, the precise structural mechanism, whether it primarily involves inhibition of ligand binding or receptor clustering, remains to be elucidated in future studies. Additionally, considering that the expression pattern of CD248 in other organs fibrosis, such as the liver, lungs, and skin might be consistent with that in renal fibrosis^41,45^, we hypothesize that targeting CD248 could potentially inhibit fibrosis in these tissues as well, despite the lack of direct validation.

## Methods

### Patients and specimens

This study was approved by the ethics committee of Xijing Hospital, Air Force Medical University (Xi’an, China), and all patient samples were collected with informed consent. The study cohort consisted of 56 patients with CKD who underwent percutaneous renal biopsy at our institution. This cohort included both male and female patients, with ages ranging from 24 to 79 years, and was representative of common CKD etiologies, including Diabetic Nephropathy, Lupus Nephritis, Hypertensive Nephropathy, and Chronic Glomerulonephritis. For control tissues, we utilized paraffin-embedded sections from 7 archived, pathologically normal nephrectomy samples from our hospital’s tissue bank. Comprehensive clinical and demographic data, including disease etiology, eGFR, and serum creatinine levels, were collected from electronic medical records. A detailed summary of patient characteristics is provided in Supplementary data 4.

### Animal models

All animal experiments in this study were approved by the Laboratory Animal Welfare and Ethics Committee of Air Force Military Medical University (Approval No. 20241143) and were conducted in accordance with the institutional Guidelines for the Care and Use of Laboratory Animals. The mice were housed in a controlled environment with a 12-h light/12-h dark cycle and provided with ad libitum access to food and water. For genetic mice lines, all mice were of the C57BL/6 J background. *Cd248*^*CreERT*^ mice, *Rosa26*^*LSL-TdTomato-DTR*^ mice, *Co1la2*^*CreERT*^ mice and *Cd248*^*fl/fl*^ mice were generated, and purchased from Shanghai Model Organisms Center, Inc. *Cd248*^−/−^ mice^37^, *Cd248*^*CreERT*^ mice, and *Yap1*^*fl/fl*^ mice^30^ were also obtained as described previously. For renal fibrosis induction, male and aged 8- to 10-weeks and weighing 20–30 g, were utilized. Mice were anesthetized via intraperitoneal injection of ketamine (100 mg/kg) and xylazine (10 mg/kg), while maintaining a body temperature of 37.8 °C on a warming pad. For the UUO model, from an incision below the left costovertebral angle, the left ureter was meticulously dissected from the adjacent tissues, and subsequently subjected to double ligation. For the uIRI model, using the same incision, the left renal pedicle was clamped for a duration of 40 min. To prevent dehydration during the surgical procedure, the mice were administered intraperitoneal injections of sterile PBS.

### Cell culture

Primary renal fibroblasts were isolated from C57BL/6 J mice. In general, kidneys were minced and digested for 40 min at 37 °C in dissociation buffer containing 1 mg/ml collagenase I (#SCR103, Sigma-Aldrich) and IV (#C4-28-100MG, Sigma-Aldrich) in serum-free high glucose DMEM. After diluted with serum-free medium and centrifuged at 200 *g* for 10 min, the pellets containing mixture of cells were resuspended and plated on a 10-cm dish. After passaged 2 generations, a high purity of fibroblasts was gained. Fibroblasts were cultured in 10% FBS DMEM treated with TGF-β (5 ng/ml), and culture medium was changed once a day for 7 days. The 3 to 5 passages of primary fibroblasts were used in our experiments. In addition, the human fetal lung fibroblast cell line HFL1 was purchased from the ATCC. HFL1 cells were maintained in DMEM/F12 supplemented with 10% fetal bovine serum and 1% penicillin–streptomycin at 37 °C in a humidified incubator with 5% CO₂. Cells were routinely tested for mycoplasma contamination and were negative.

### Lentiviral transduction and generation of stable CRISPR–Cas9 knockout cell lines

To achieve the overexpression of CD248 constructs, we synthesized four vectors: EGFP–CD248, ΔECD (amino acids 715–766), and ΔCTLD (amino acids 157–766). These vectors were then cloned into the Ubi-MCS-SV40 lentiviral vector. For the CRISPR–Cas9-based deletion of *Cd248*, we designed sgRNAs targeting the coding regions of murine Cd248 and cloned them into the lentiCRISPRv2 lentiviral vector (Gecko, Addgene). The sgRNAs were carefully designed using algorithms from crispor.tefor.net to minimize off-target effects, and the oligonucleotide sequence of the sgRNA used was TCCAGGAATGTGCGGCGCCG. Lentiviruses were generated in HEK293T cells through co-transfection with pCMV-VSV-G and psPAX2 vectors, employing Lipofectamine 2000 as per the manufacturer’s guidelines. After 12 h, the culture medium was replaced with DMEM containing high glucose and enriched with 10% FBS. Viral particles were harvested 24 h later. The collected viral supernatants underwent centrifugation and sterile filtration before being mixed at a 1:1 ratio with the primary fibroblast culture medium. This medium was further enhanced with 6 μg/ml of polybrene (Sigma-Aldrich) for all cell cultures. The fibroblasts were then exposed to the virus-containing medium. Following a 24-h incubation, fresh culture medium fortified with 2 μg/ml of puromycin was introduced to the cells. The cells underwent selection for a duration of 5 to 7 days. The effectiveness of the transduction process was subsequently confirmed through western blotting.

### RNAscope

For RNAscope, the sections were subjected to staining using the corresponding reagents as outlined in the protocol of the RNAscope Multiplex Fluorescent Reagent Kit v2 Assay (#323120, ACD, USA). Following the processes of deparaffinization and rehydration, antigen retrieval was performed using 1X target retrieval reagent in a steamer, followed by rinsing the slides for 15 s and transferring them to 100% ethanol for a duration of 3 min, after which they were allowed to dry at room temperature. The slides were then covered with RNAscope protease plus and incubated in a pre-warmed oven. Subsequently, the sections underwent incubation with a combination of probes, followed by reaction with AMP1, AMP2, and AMP3 reagents. Subsequently, they were counterstained with HRP-C1, HRP-C2, and HRP-C3, respectively. Lastly, each slide was counterstained with DAPI and mounted using Prolong Gold Antifade Mountant (#P36930, ThermoFisher, USA). Images were taken by an Olympus VS200 system. The ratio of ecm-Fibs, CCL2^hi^ Fibs, or CCL19 & 21^hi^ Fibs to the total fibroblasts was determined to conduct subsequent correlation analysis.

For combined RNAscope and immunofluorescence staining, the single-plex RNAscope assay was completed first. Immediately thereafter, sections were subjected to the immunofluorescence protocol. Briefly, slides were blocked for 1 h at room temperature, followed by 2–3 h incubation with the primary antibody at rom temperature. After washing, sections were incubated with the appropriate Alexa Fluor488-conjugated secondary antibody for 1 h before being counterstained with DAPI and mounted for imaging.

### Atomic force microscopy measurement

The stiffness of kidney tissues was assessed through the utilization of Atomic Force Microscopy (AFM). In this study, fresh kidney tissues obtained from mouse models were collected and subsequently embedded in Optimal Cutting Temperature (OCT) compound (#4583, SAKURA, Japan). Micromorphology imaging of the specimens was conducted using AFM (Keysight 5500, Keysight Technologies, Santa Rosa, USA) in tapping mode. A silicon probe (PPP-NCLR-20, Nanosensors) with a force constant of 42 N/m and a resonance frequency of 161 kHz was consistently employed throughout the experimental procedure. The mechanical properties were assessed through AFM-based nanoindentation. Initially, the specimens were submerged in PBS at ambient temperature. For the nanoindentation process, a Silicon Oxide tip with a resonance frequency of 13 kHz and a force constant of 0.2 N/m (SD-Sphere-CONT- M-10, Nanosensors) was employed in the tapping mode. The elastic modulus of each location was determined at an indentation depth of 300 nm. This procedure was replicated six times for each tissue specimen, and the resulting values were averaged.

### Preparation of PDMS substrates with varying stiffness

Polydimethylsiloxane (PDMS) cell culture substrates with varying stiffness (soft and stiff) were fabricated. PDMS mixtures were prepared by thoroughly combining PDMS prepolymer A and crosslinker B (Dow corning, USA). For soft substrates, prepolymer A and crosslinker B were mixed at a ratio of 99:1 (v/v), whereas for stiff substrates, a ratio of 19:1 was used. Following mixing, the PDMS mixtures were then poured into appropriate culture and subsequently transferred to a drying oven and incubated at 65 °C overnight to ensure complete polymerization.

Prior to cell culture, the cured PDMS surfaces were functionalized with dopamine. A 0.2% dopamine solution was added to cover the PDMS surfaces, and the substrates were incubated in a cell culture incubator for 2 h. Subsequently, the dopamine solution was aspirated, and the PDMS surfaces were rinsed three times with sterile PBS, with each rinse lasting 10 min.

### Single-cell RNA-seq data processing

The FASTQ files, obtained from publicly available source, that were produced by 10× Genomics, was aligned and quantified against the GRCh38 human reference genome. This alignment and quantification process was carried out using the Cell Ranger software (Version 6.1.2) with default settings. The resulting output from the cellranger and count matrix were read using the “Read10X” function from the Seurat package (Version 4.3.0). The count matrix was subsequently converted to “dgCMatrix” format. The “RenameCells” method was used to ensure that all cell labels were unique. Furthermore, quality control was applied to the cells based on several criteria. Briefly, cells with <200 detected genes as well as those with > 20% mitochondrial content was removed. Furthermore, Cells having over 6000 detected genes were eliminated to further exclude the possible doublets. After applying a filtering process, a total of 51,713 cells were deemed qualified for subsequent analyses. To ensure uniform gene expression across cells, the “LogNormalize” function was utilized with a scale factor of 10,000. The top 2000 genes exhibiting variability in expression were selected for further analysis using the “FindVariableFeatures” method. To eliminate unwanted sources of variation, the “ScaleData” function was employed with the “vars.to.regress” option, considering UMI and percent mitochondrial content. The dimensionality of the dataset was reduced by incorporating highly variable features into Principal Component Analysis (PCA), resulting in the identification of the first 30 principal components for subsequent analysis. Clustering analysis was performed by considering the edge weights between cells, and a shared nearest-neighbor graph was generated using the Louvain algorithm, which was implemented through the “FindNeighbors” and “FindClusters” functions. The UMAP method was employed to visualize the identified clusters. In order to conduct subclustering analysis, a comparable procedure was implemented, encompassing normalization, selection of variably expressed features, reduction of dimensions, and identification of clusters. To annotate the cell clusters, differentially expressed markers of the resultant clusters were determined using the “FindAllMarkers” function, employing the default nonparametric Wilcoxon rank sum test with Bonferroni correction.

### Trajectory analysis

The Single-Cell Trajectories analysis with Monocle (Version 2.22.0) algorithm was employed, utilizing DDR-Tree and default parameters. Positive marker genes for each cluster were utilized in the Monocle analysis. Branch expression analysis modeling was then applied to determine gene analysis for branch fate based on pseudotime analysis. The “ReduceDimension” function was utilized to reduce the space to two dimensions, while the orderCells function ordered the cells according to gene expression. Pseudotime-dependent genes were determined through the utilization of the “differentialGeneTest” tool, employing the “fullModelFormulaStr” option “~sm.ns (Pseudotime)”. Subsequently, smooth expression curves were generated using the “plot_pseudotime_heatmap” function. The Ggridges package was employed to examine the distribution of cells across various groups along the pseudotime axis.

### Transcription factor analysis

Cells that were confirmed to be in different states using Monocle were subsequently included in the SCENIC package (Version 1.2.4) for single-cell regulatory network inference and clustering. These cells were then sorted based on their clusters and states. To eliminate noise, genes with low expression levels or low positive rates were filtered out using the “gene-Filtering” method with default settings. Furthermore, only the genes that aligned with the Rcis target databases were retained for further analysis. Following the reconstruction of the gene regulatory network, GENIE3 successfully identified the association between transcription factors and their potential targets. The assessment of regulon enrichment in individual cells was conducted by employing the “aucell” positional argument.

### Enrichment analysis

The differential genes were subsequently analyzed for enrichment using the clusterProfiler package (Version 3.0.4) with default settings, through Gene Ontology (GO) and Kyoto Encyclopedia of Genes and Genomes (KEGG) analysis. For the Gene Set Enrichment Analysis (GSEA) of clusters c2/c5, the Hallmark gene sets from the MSigDB database (https://www.gsea-msigdb.org/gsea/msigdb) were utilized.

### Correlation to public datasets

A merged cohort was created by downloading and normalizing a microarray-based study of CKDs from the GEO or ArrayExpress database. Initially, the “Combat” function of the sva package was utilized to eliminate batch effects between cohorts sharing the same platform. Furthermore, batch effects between different platforms were also addressed. To validate the effectiveness of Combat, a 3D PCA analysis was conducted.

For correlation analysis, fibroblast subclusters and matrisome-related genes were used to define gene sets by ssGSEA.

### Generation of bulk RNA-sequencing

Total RNA was extracted using TRIzol reagent (#15596018, ThermoFisher, USA) following the manufacturer’s procedure. The total RNA quantity and purity were analysis of Bioanalyzer 2100 and RNA 6000 Nano LabChip Kit (#5067-1511, Agilent, USA), high-quality RNA samples with RIN number > 7.0 were used to construct sequencing library. Then the cleaved RNA fragments were reverse-transcribed to create the cDNA by SuperScript™ II Reverse Transcriptase (Invitrogen, cat. 1896649, USA), which were next used to synthesise U-labeled second-stranded DNAs with E. coli DNA polymerase I (NEB, cat.m0209, USA), RNase H (NEB, cat.m0297, USA) and dUTP Solution (#R0133, ThermoFisher, USA). At last, the 2 × 150 bp paired-end sequencing (PE150) was performed on an Illumina Novaseq™ 6000 following the vendor’s recommended protocol. to get high quality clean reads, reads were further filtered by Cutadapt (https://cutadapt.readthedocs.io/en/stable/, version: cutadapt-1.9). Then sequence quality was verified using FastQC (http://www.bioinformatics.babraham.ac.uk/projects/fastqc/, 0.11.9). including the Q20, Q30 and GC-content of the clean data. After that, a total of G bp of cleaned, paired-end reads were produced.

### Purification of monoclonal antibody IgG78

The construction, purification, and in vivo and in vitro activity characterization of IgG78 were performed as previously reported^44^. Generally, the coding sequences for the variable regions of both the heavy and light chains of scFv78 were analyzed using the IgBlast database to identify their corresponding constant region sequences. These variable and constant region sequences were then combined, optimized, synthesized, and cloned into the bicistronic eukaryotic expression vector Lh1, resulting in the construct named Lh1-IgG78. Following this, Lh1-IgG78 was transiently transfected into HEK293F cells. IgG78 was subsequently purified using Protein A affinity chromatography. Subsequently, The purity and integrity of IgG78 were assessed by mass spectrometry and SDS-PAGE, while its binding affinity was evaluated using cellular ELISA.

### In vivo treatments

To induce Cre expression via tamoxifen (TAM), mice were administered 2 mg of TAM (Sigma, #T5648) diluted in sunflower seed oil (Sigma, #88921) intraperitoneally for five consecutive days. For the ablation of CD248-positive fibroblasts during renal fibrosis, mice received intraperitoneal injections of 25 ng/g diphtheria toxin (DT) (Enzo Life Sciences, BML-G135) twice weekly. For anti-CD248 studies, mice were treated with isotype antibodies or anti-CD248 (IgG78) antibodies (in-house). The first dose was given at 10 mg/kg, followed by 5 mg/kg thereafter administered intravenous injection twice per week.

### Histology and immunohistochemistry of tissues

The kidney tissues were fixed in a 10% formalin and subsequently embedded in paraffin. Following deparaffinization and rehydration, the sections were subjected to antigen retrieval using a citrate buffer. Subsequently, the sections were blocked with goat serum for 40 min. The primary antibody was then applied to the sections and incubated overnight at 4 °C in a humidified box. Following this, the sections were incubated with corresponding HRP-labeled secondary antibodies (#ab6721, Abcam) for a duration of 30 min at room temperature. Finally, visualization was achieved using a 3,3’-diaminobenzidine system (#DAB-0031, MXB, China). The sections were counterstained using hematoxylin and subsequently dehydrated in increasing concentrations of ethanol, followed by clearance with xylene and permanent coverslipping for the purpose of observation. The following antibodies were used: anti-CD248 (# ab204914, Abcam); anti-ZEB1 (#303480, Abcam); anti-NF-κB1 (#AF3219, Affinity Bioscience); anti-Tenascin C (#ab108930, Abcam); anti-α-SMA (#14395-1-AP, Proteintech); anti-Fibronectin (#15613-1-AP, Proteintech); Images were taken by an Olympus VS200 system.dd.

For Periodic Acid-Schiff stain (PAS), Masson’s Triton staining, Sirius Red staining, and HE staining, after deparaffinization and rehydration, sections were stained with corresponding reagents according to the manufacturer’s protocol (Servicebio, China).

For quantitative analysis, using the threshold function, the image was processed so that positive staining was represented by strong positive brown pixel for CD248 staining and blue pixel for Masson’s Triton staining, or red pixel for Sirius Red staining (collagen). Then, the pixels were measured as a percentage of the area of total image analyzed through Image J software.

### Immunofluorescence

For multiplexed IHC, after deparaffinization and rehydration, sections were stained with corresponding reagents according to the manufacturer’s protocol (AKOYA Bioscience). Briefly, antigen retrieval was done in retrieval buffer at 85 °C for 20 min, followed by incubation in 3% hydrogen peroxide for 15 min, and then blocking buffer was used for 10 min at room temperature. After incubation of 1 h at room temperature, the primary antibody was detected using a secondary antibody conjugated to horseradish peroxidase followed by chromogenic revelation using tyramide signal amplification (TSA) fluorescence system. Then the same slides were leached and restained as previously described. The following antibodies were used: anti-CD248 (#ab204914, Abcam); anti-COL1A1 (#E8F4L, CST); anti-αSMA (#14395-1-AP, Proteintech); anti-^pY118^Paxillin (#E9U9F, CST); anti-Fibronectin (#15613-1-AP, Proteintech); anti-YAP (#13584-1-AP, Proteintech); anti-p65 (#ab32536, Abcam); anti-DCN (#ab277636, Abcam); anti-POSTN (#ab215199, Abcam); anti-PDGFRB (#ab69506, Abcam); anti-CD3 (#ab16669, Abcam); anti-CCR7 (#ab32524, Abcam); anti-CD68 (#25747-1-AP, Proteintech); anti-CCR2 (#ab273050, Abcam); F-actin (#p1951, Sigma-Aldrich).

### Tissue processing

fibrotic kidneys were harvested and minced with scissors, then enzymatically digested in CO_2_-independent incubator shaker (#ISF1-XC, Kuhner, Germany) with 1 mg/ml Collagenase I (#SCR103, Sigma-Aldrich) and IV mixture (#C4-28-100MG, Sigma-Aldrich) for 1 h at 37 °C under 80 rpm agitation. After diluted with serum-free medium and centrifuged at 200 *g* for 10 min, the cell pellets were resuspended in ACK lysis buffer (#NC9067514, ThermoFisher) to remove blood cells. Prior to staining, the cell suspended in FACS buffer comprised of PBS with 5% BSA (#SRE0096, Sigma-Aldrich) was filtered through a 40 μm mesh (#BS-40-XBS, Biosharp).

### Cell population analysis by flow cytometry

Cells were incubated with the Zombie-Aqua Fixable Viability Kit (#423101, BioLegend) for 20 min at room temperature. Subsequently, the cells were washed with PBS containing 1% FBS. Following this, the cells were incubated with Anti-EpCAM (#118214, BioLegend), anti-CD45 (#157212, BioLegend), and anti-CD31 (#160204, BioLegend). After being washed twice with wash buffer, flow cytometry analysis was performed.

### Flow cytometry sorting

After incubation with antibodies, live cells were processed to remove epithelial, immune, and endothelial cells using EpCAM, CD45, and CD31 markers, respectively. Subsequently, triple-negative cells were sorted, and TdTomato was employed to categorize fibroblasts into CD248-negative and CD248-positive populations. For the collection of sorted cells, 2 mL of TRIzol (#T9424, Sigma-Aldrich) or complete DMEM was added to 15-mL collection tubes. Flow cytometry sorting was performed using the BD FACSCelesta system.

### qRT-PCR

Total RNA was isolated with TRIzol. Reverse transcription (RT) was conducted using PrimeScript^TM^ RT Master Mix (#RR036A, TaKaRa). Then qPCR was performed using an SYRB GreenⅡ kit (#DRR041A; TaKaRa). The sequences of the primer pairs for different genes are described in the Supplementary Data 8. The mRNA levels of genes were calculated after normalizing with *Gapdh*.

### Western blot (WB)

Total protein samples were prepared from tissues or cell pellets and concentration was determined by the BCA assay (#23227, ThermoFisher, USA). Equal amounts of protein samples were loaded on 10% sodium dodecyl sulfate-polyacrylamide gel (SDS-PAGE) and then transferred onto a polyvinylidene fluoride (PVDF) membrane (ThermoFisher). The membrane was blocked with 5% skim milk, and incubated with primary antibodies overnight at 4 °C: anti-CD248 (#sc-377221, Santa Cruz Biotechnology); anti-COL1A1 (#E8F4L, CST); anti-Fibronectin (#15613-1-AP, Proteintech, China); anti-α-SMA (#14395-1-AP, Proteintech); anti-CTGF (#25474-1-AP, Proteintech); anti-FAK (phospho Y397) (#ab81298, Abcam); anti-FAK (#3285, CST); anti-Paxillin (phospho Y113) (#ab32084, CST); anti-Paxillin (#50195, CST); anti-GAPDH (#10494-1-AP, Proteintech); anti-YAP1 (#13584-1-AP, Proteintech); anti-FLAG (#20543-1-AP, Proteintech); Membranes were then incubated with horseradish peroxidase (HRP)-conjugated secondary antibodies for 1 h at room temperature. Uncropped scans of all blots are provided in the Source Data file.

### Hydroxyproline content assay

The hydroxyproline content in kidney tissues was assessed utilizing a commercially available kit in accordance with the guidelines provided by the manufacturer (#K226, BioVision).

### Second harmonic generation

Coating slides with 0.2% gelatin solution at 37 °C for 1 h, then adding 2 mL of 1% glutaraldehyde solution and incubating at room temperature for 30 min, followed by incubation with 1 M isobutanol at room temperature for 30 min. Wash with DPBS^+^ (DPBS containing Ca^2+^ and Mg^2+^) solution until isobutanol is removed. Primary fibroblasts were seeded into 24-well plates, and after attachment, the medium was aspirated and replaced with complete medium containing 100 μg/mL ascorbic acid (initially, the concentration of ascorbic acid was adjusted to 500 μg/mL). The medium was changed every 24 h for 8 days. Cells were then treated with extraction reagent (0.5% (v/v) Triton X-100, 20 mM NH_4_OH) and incubated at 37 °C for 3–5 min. After washing with DPBS^+^ solution, the next step was to perform SHG imaging. All samples were imaged using an FVMPE-RS multiphoton confocal microscope with a 20× objective throughout the experiments. The excitation wavelength was set to 920 nm, and a 120 ± 5 nm narrow bandpass emission filter controlled by a slit was used to detect the SHG signal of collagen. The SHG signal is generated when two photons of incident light interact with the non-centrosymmetric structure of collagen fibers, resulting in photons with half the wavelength of the incident photons.

### Examination of kidney function and liver function

Mice BUN (#E-BC-K183-M, Elabscience), serum Cr (#E-BC-K188-M, Elabscience), AST (#E-BC-K236-M, Elabscience) and ALT (E-BC-K235-M, Elabscience) was detected by reagent kits in accordance with the guidelines provided by the manufacturer.

### Statistics and reproducibility

For all representative images, experiments were repeated at least three times independently with similar results unless otherwise stated. The data were expressed as means ± s.d. Multigroup comparison was conducted using one-way analysis of variance (ANOVA) followed by the Tukey multiple comparison test for subgroup comparison. Two-group comparison was analyzed using two-tailed Student’s *t* test (α = 95%). The Pearson correlation coefficient was determined using Prism 8 (GraphPad). A significance level of *P* < 0.05 was used to determine statistical significance.

### Reporting summary

Further information on research design is available in the Nature Portfolio Reporting Summary linked to this article.

## Supplementary information

Supplementary Figs.

Description of Additional Supplementary Files

Supplementary Data

Reporting Summary

Transparent Peer Review file

## Data Availability

The ScRNA-seq data of CKD patients analyzed in this study are available on Zenodo at https://zenodo.org/record/4059315. The human kidney transplant bulk RNA-seq dataset is available from ArrayExpress under accession code E-MTAB-2502^46^. The human kidney biopsy bulk RNA-seq dataset is available from the Gene Expression Omnibus (GEO) under accession code GSE66494^47^. The mouse renal fibroblast bulk RNA-seq data generated in this study have been deposited in GEO under accession code GSE241634. Source data are provided with this paper in the accompanying Source Data file.

## Code availability

Custom scripts used for single-cell and bulk RNA-seq analyses are available at GitHub: https://github.com/RangerXc/CKD.
