# Supplementary material for: CD248 acts as a mechanosensory switch in fibroblast subsets to establish distinct pathological niches in renal fibrosis
Source: Nat Commun. 2026 May 6;17:7361. doi: 10.1038/s41467-026-72187-0 (PMC13402351; doi:10.1038/s41467-026-72187-0)
Supplement: Supplementary file 1 — Supplementary Figs. [file 41467_2026_72187_MOESM1_ESM.pdf]

Supplementary figure

Figure S1

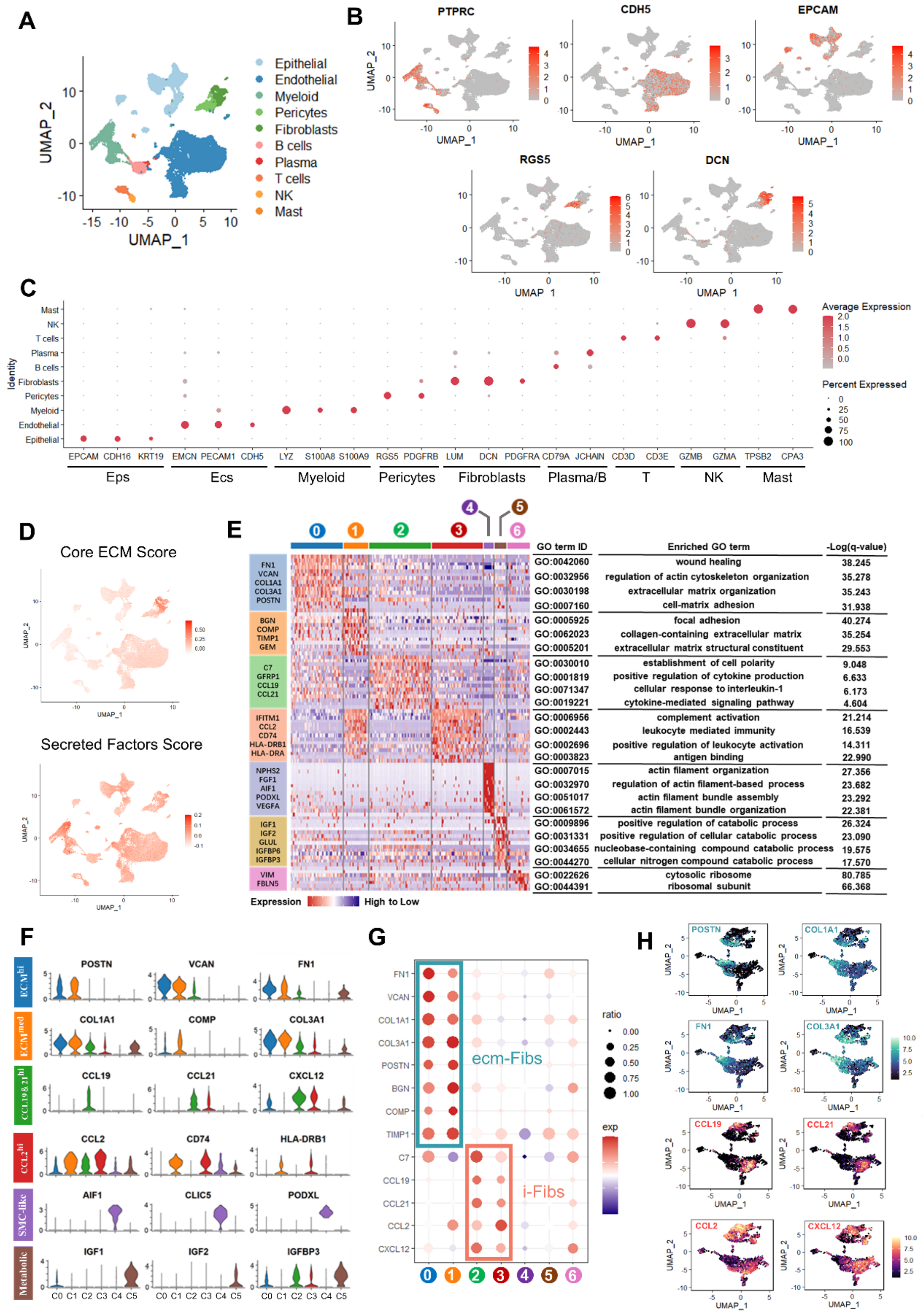

Figure S1. ScRNA-seq analysis identified fibroblast heterogeneity in the fibrogenic niche of kidney. (**A**) UMAP plot of major cell types of kidneys with CKD. (**B**) UMAP plot showing specific markers for annotating major cell types of kidneys. (**C**) Bubble plot showing specific markers for annotating major cell types of kidneys. (**D**) UMAP plot indicating gene sets score among major cell types. (**E**) Left, heat map showing the relative average expression of the most significantly enriched genes within each cluster, as determined by the log fold-change of cells within a cluster compared with that of all other cells in the dataset. Representative genes are specifically highlighted for each cluster. Right, significantly enriched GO terms for each cluster are shown. (**F**) Selected markers distinctly expressed among the identified fibroblast clusters. (**G**) Bubble plot showing selected zpro-inflammatory and pro-fibrotic genes. (**H**) UMAP showing selected pro-fibrotic (above) and pro-inflammatory (below) genes.

Figure S2

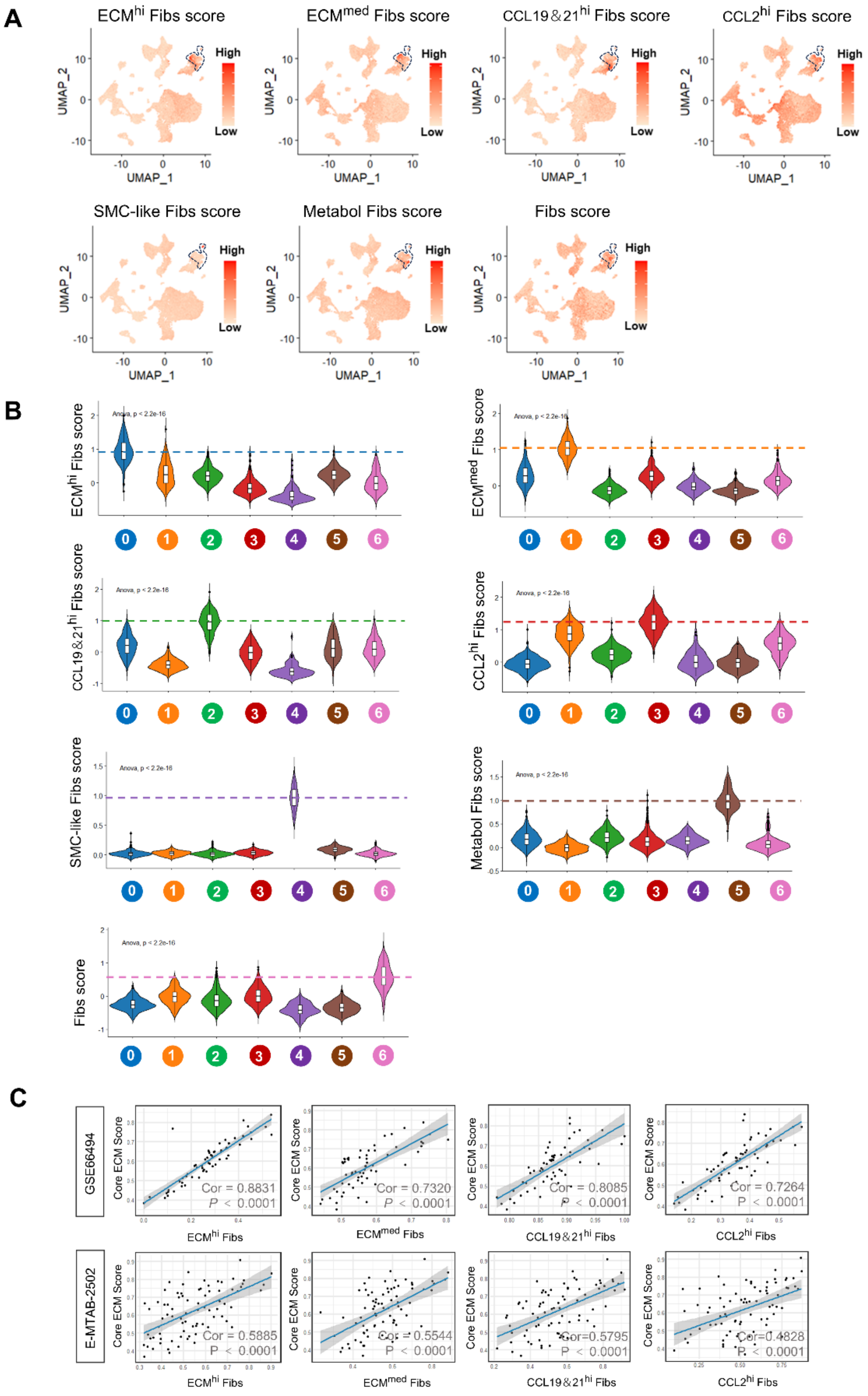

Figure S2. Single-cell sequencing analysis reveals two distinct fibroblast subpopulations with pro-inflammatory and pro-fibrotic functions. (A) UMAP plot

showing specific gene signatures of diverse fibroblast subpopulation. (**B**) Violin plot showing specific gene signatures of diverse fibroblast subpopulation. (**C**) Correlation analysis showing the relationship between ecm- ,or i-Fibs and core ECM score of matrisome based on public mRNA-seq datasets (E-MTAB-2502 and GSE66494, respectively). All data are represented as mean ± s.d. Statistics were calculated using one-way ANOVA.

Figure S3

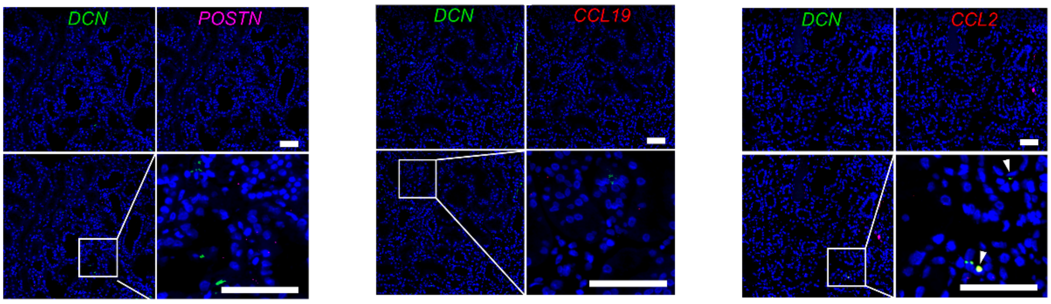

Figure S3. RNAscope staining showing the distribution of diverse fibroblasts subsets in normal human kidney.

Figure S4

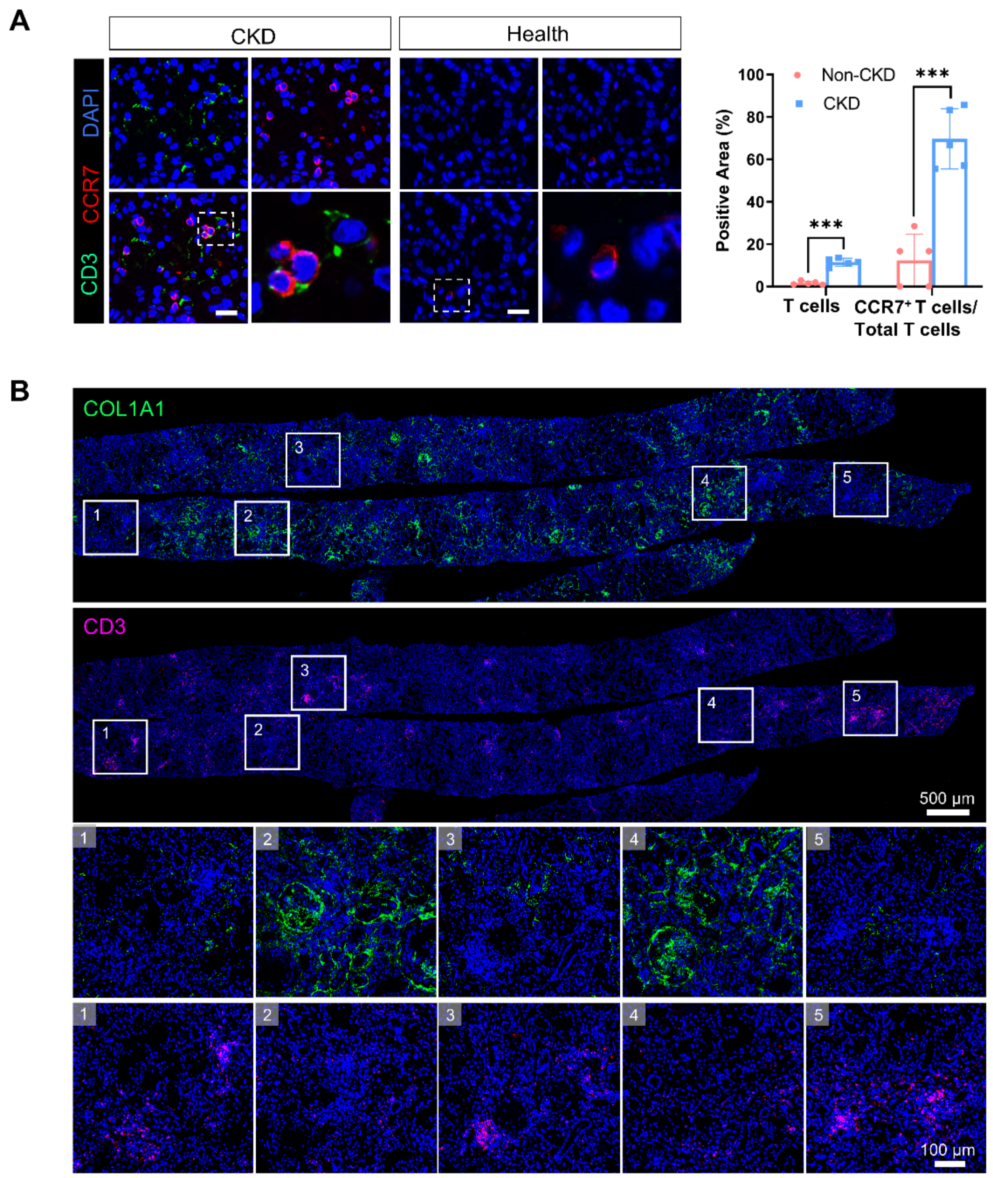

Figure S4. Spatial segregation of focal immunoreactive and fibrogenic lesions within injured kidney. (**A**) Representative immunofluorescent staining for CD3 and CCR7, and quantification of the abundance of T cells and $CCR7^+$ T cells in normal and CKD samples. (**B**) Representative immunofluorescent staining for COL1A1 and CD3. ***$P < 0.001$. Scale bar, 100 μm. All data are represented as mean ± s.d from $n \geq 3$ independent experiments. Statistics were calculated using two-tailed, unpaired Student's *t* test. Source data are provided as a Source Data file.

Figure S5

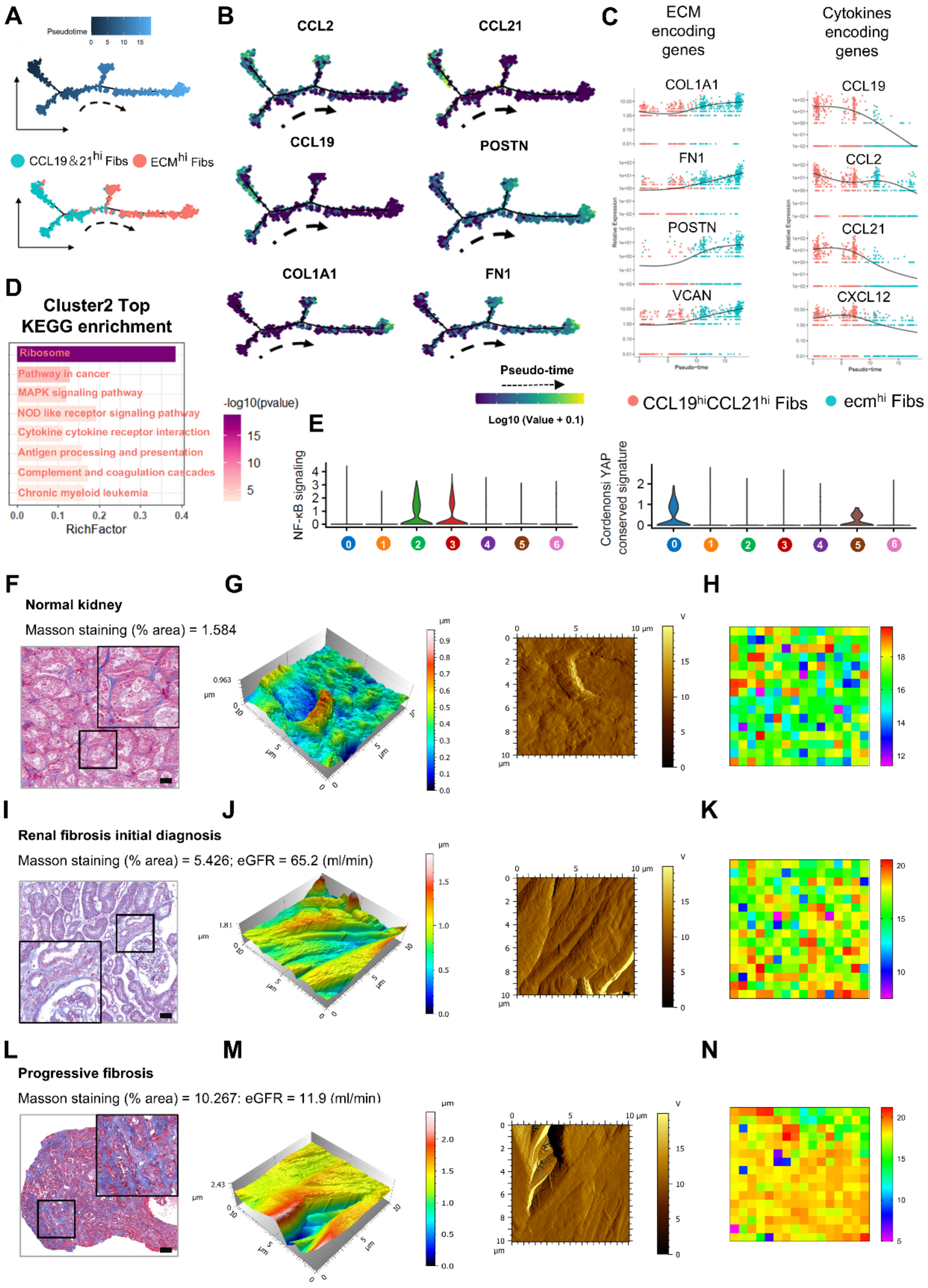

Figure S5. i-Fib to ECM-Fib differentiation is linked to stromal stiffening. (A) Differentiation trajectory between ecm^{hi} Fibs and CCL19&21^{hi} Fibs predicted using monocle 2. (**B**) Altered gene expression analysis in the evolutionary trajectory from

CCL19&21^{hi} Fibs to ecm^{hi} Fibs using monocle 2. (**C**) Changing trend of selected ECM and cytokine genes in the process of differentiation of CCL19&21^{hi} Fibs into ecm^{hi} Fibs. (**D**) Top enriched KEGG functions of gene cluster 1. (**E**) Violin plot showing gene sets score among all fibroblast subclusters. (**F to N**) Masson staining (F, I and L), AFM scanning three-dimensional reconstruction map of stromal matrix (G, J and M) and stiffness map of the renal matrix (H, K and N). Scale bar, 100 μm. Source data are provided as a Source Data file.

Figure S6

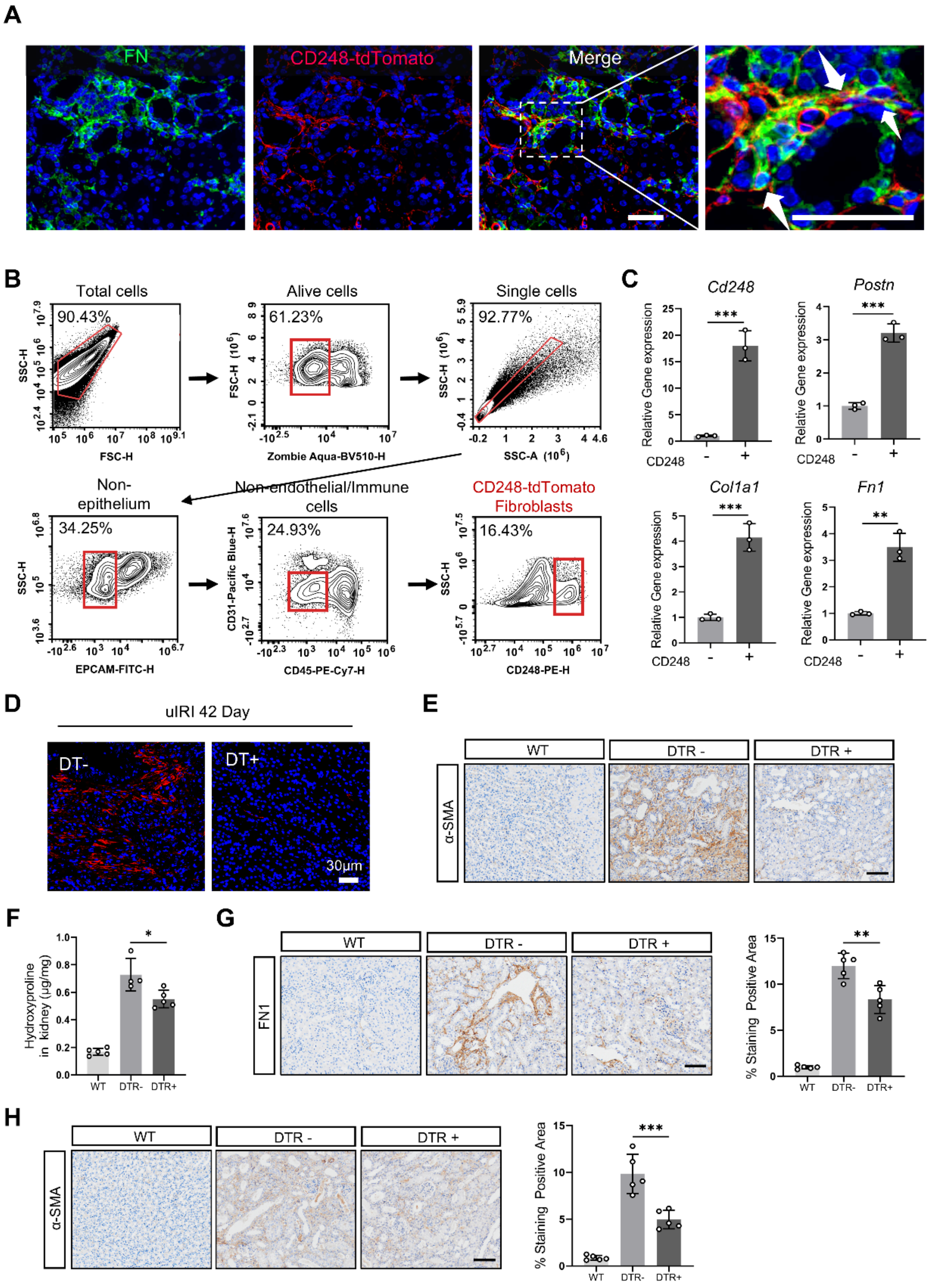

Figure S6. Loss of CD248⁺ myofibroblasts ameliorates fibrotic progression of kidney, related to figure 3. (A) Representative immunofluorescence images of specified proteins in mice after 42 days with uIRI injury (n = 3). Scale bar, 50 μm.

Arrow indicates $CD248^{+}$ FN^{+} myofibroblasts. (**B**) Gating strategy to sort CD248- fibroblasts and $CD248^{+}$ myofibroblasts by FACS. (**C**) mRNA expression of *Cd248*, *Postn*, *Col1a1*, and *Fn1*, normalized to *Gapdh* (n = 3). (**D**) Representative immunofluorescence images of $CD248^{+}$ myofibroblasts in $Cd248^{CreERT;\ tdTomato\text{-}DTR}$ uIRI mice with or without diphtheria toxin administration (n = 3). Scale bar, 30 µm. (**E**) Representative images of α-SMA staining in uIRI kidney (n = 5), following DT administration in $Cd248^{CreERT;\ TdTomato\text{-}DTR}$ and $Cd248^{wt/wt;\ TdTomato\text{-}DTR}$ mice. Scale bar, 50 µm. (**F**) The hydroxyproline content in mouse kidney tissues with UUO injury (n = 4 to 5 per group). (**G and H**) Representative images of FN (G) and α-SMA (H) staining in UUO kidney (n = 5), following DT administration in $Cd248^{CreERT;\ TdTomato\text{-}DTR}$ and $Cd248^{wt/wt;\ TdTomato\text{-}DTR}$ mice. Scale bar, 50 µm. Quantification of IHC staining. *$P < 0.05$, **$P < 0.01$, ***$P < 0.001$. All data are represented as mean ± s.d from $n \geq 3$ independent experiments. Statistics were calculated using two-tailed, unpaired Student's t test (C) or ANOVA with Tukey's test (F, G and H). Source data are provided as a Source Data file.

Figure S7

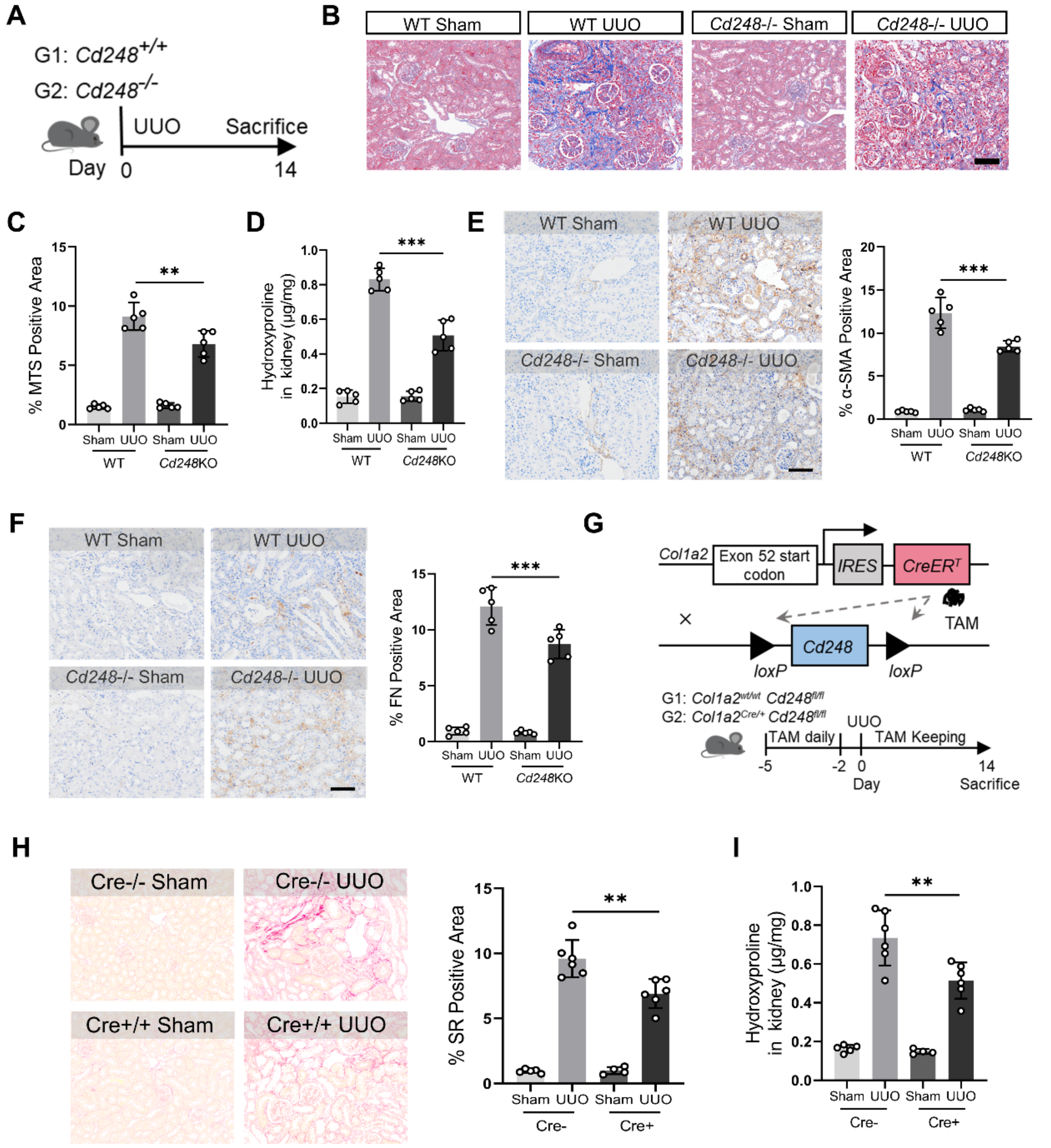

Figure S7. Specific depletion of CD248 in fibroblasts inhibited myofibroblast activation and renal fibrosis. (**A**) Schematic of experimental process of *Cd248 KO* mice with UUO. (**B** and **C**) Masson staining of kidney tissues after UUO (n = 5) (B). Scale bar, 50 μm and quantification of positive area of Masson staining (C). (**D**) The hydroxyproline content in kidney tissues of UUO mice (n = 5). (**E**) Representative images of IHC staining of α-SMA (n = 5). Scale bar, 50 μm and quantification of

positive area of α-SMA. (**F**) Representative images of IHC staining of FN (*n* = 5). Scale bar, 50 μm and quantification of positive area of FN. (**G**) Schematic of the genetic (top) and experimental (bottom) approach for the generation of $Col1a2^{CreERT;}Cd248^{fl/fl}$ mice with UUO. (**H**) SR staining of kidney tissues after UUO (*n* = 4 to 6), Scale bar, 50 μm. Quantification of positive area of SR staining. (**I**) The hydroxyproline content in murine kidney tissues with UUO (*n* = 4 to 6). $^{**}P < 0.01$, $^{***}P < 0.001$. All data are represented as mean ± s.d from $n \geq 3$ independent experiments. Statistics were calculated using one-way ANOVA with Tukey's test. Source data are provided as a Source Data file.

Figure S8

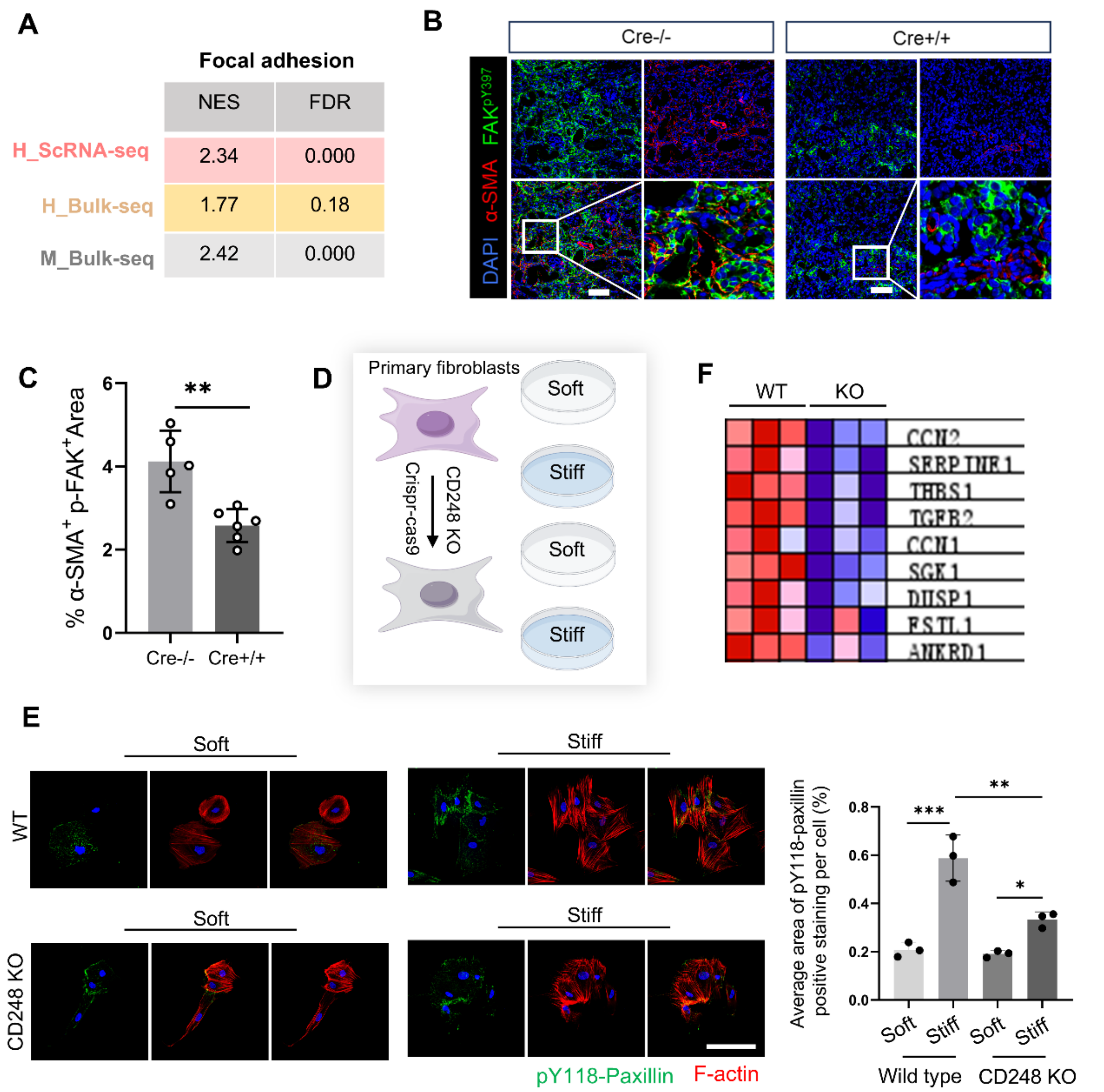

Figure S8. CD248 modulates myofibroblast activation via focal adhesion-YAP mediated mechanotransduction, related to figure 5. (**A**) Gene set enrichment analysis of focal adhesion pathway. (**B**) Representative immunofluorescence images of pY397-FAK and α-SMA in *Col1a2*$^{wt/wt;}$*Cd248*$^{fl/fl}$ (*n* =5) and *Col1a2*$^{CreERT;}$*Cd248*$^{fl/fl}$ (*n* =6) mice with uIRI injury. Scale bar, 50 µm. (**C**) Quantification of positive area of pY397-FAK^{+} myofibroblasts. (**D**) Schematic of WT and CD248 KO primary fibroblast responses to soft versus stiff substrates. (**E**) Immunofluorescence analysis of pY118-

paxillin and F-actin among primary renal fibroblasts with or without CD248KO in soft or stiff substrates. Scale bar, 100 μm. The right panel shows the quantification of the average area of pY118-paxillin positive staining per cell (%). (**F**) Heatmap of the gene set enrichment pathway analysis of top genes of Cordenonsi YAP conserved signature. $**P < 0.01$, All data are represented as mean ± s.d from $n \geq 3$ independent experiments. Statistics were calculated using two-tailed, unpaired Student's *t* test (C). Source data are provided as a Source Data file.

Figure S9

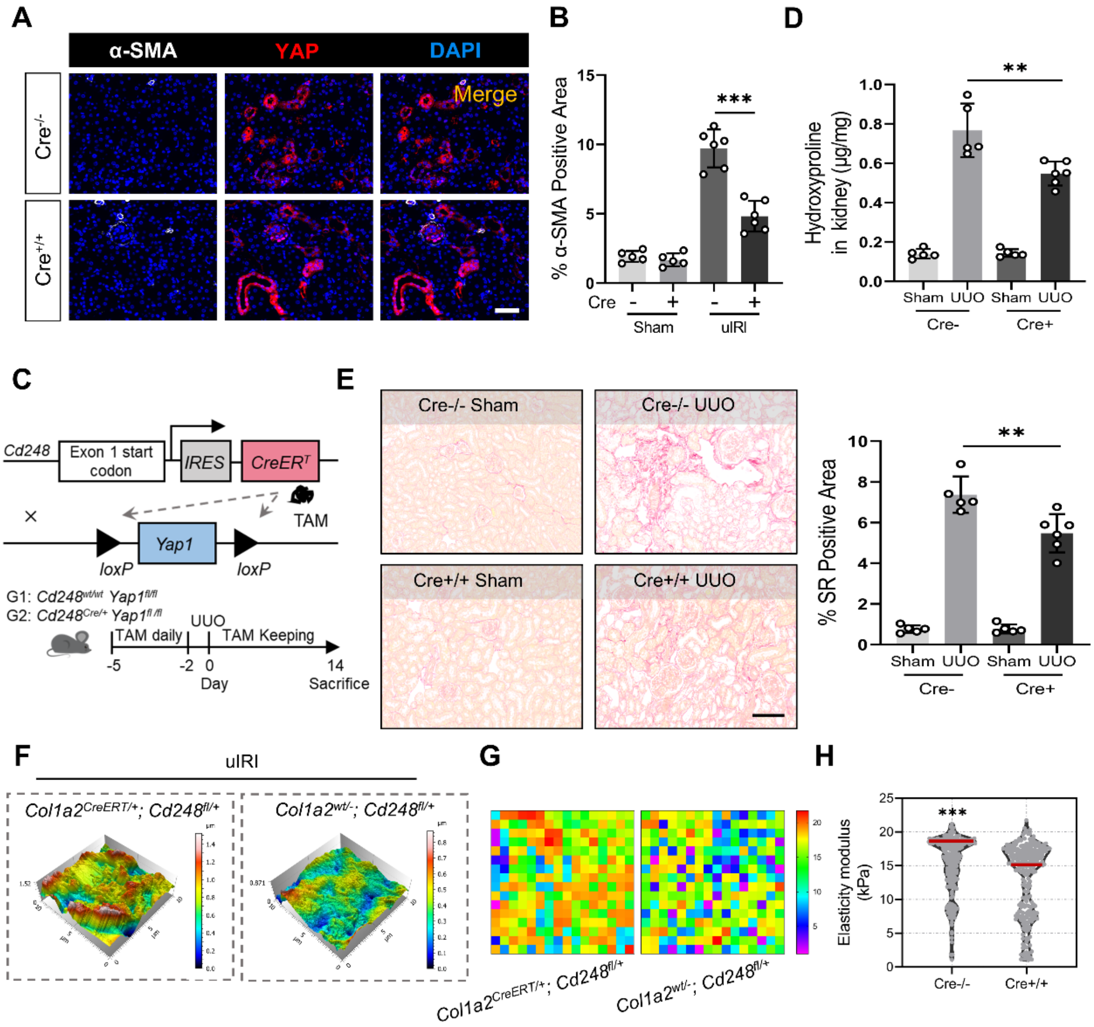

Figure S9. Loss of YAP in CD248$^+$ myofibroblasts decreased ECM stiffness and fibrosis, related to figure 6. (**A**) Immunofluorescence analysis of α-SMA and YAP in kidney tissues of genetic mice of sham groups for uIRI injury (n = 5). Scale bar, 50 µm. (**B**) Quantification of positive area of α-SMA. (**C**) Schematic of the genetic (top) and experimental (bottom) approach for the generation of $Cd248^{CreERT;}Yap1^{fl/fl}$ mice with UUO injury. (**D**) The hydroxyproline content in murine kidney tissues with UUO (n = 5 to 6). (**E**) SR staining of kidney tissues after UUO (left), and quantification of positive area of SR (n = 5 to 6) (right). Scale bar, 50 µm. (**F**) AFM scanning three-dimensional reconstruction map of stromal matrix for kidney tissues with uIRI injury in $Col1a2^{wt/wt;}Cd248^{fl/fl}$ and $Col1a2^{CreERT;}Cd248^{fl/fl}$ mice ($n \geq 3$ biological duplication). (**G**) Representative images of stiffness map of the renal matrix measured by AFM. (**H**) Quantification of tumor matrix stiffness. **$P < 0.01$, ***$P < 0.001$. All data are represented as mean ± s.d from $n \geq 3$ independent experiments. Statistics were calculated using two-tailed, unpaired Student's t test (H) or ANOVA with Tukey's test (B, D and E). Source data are provided as a Source Data file.

Figure S10

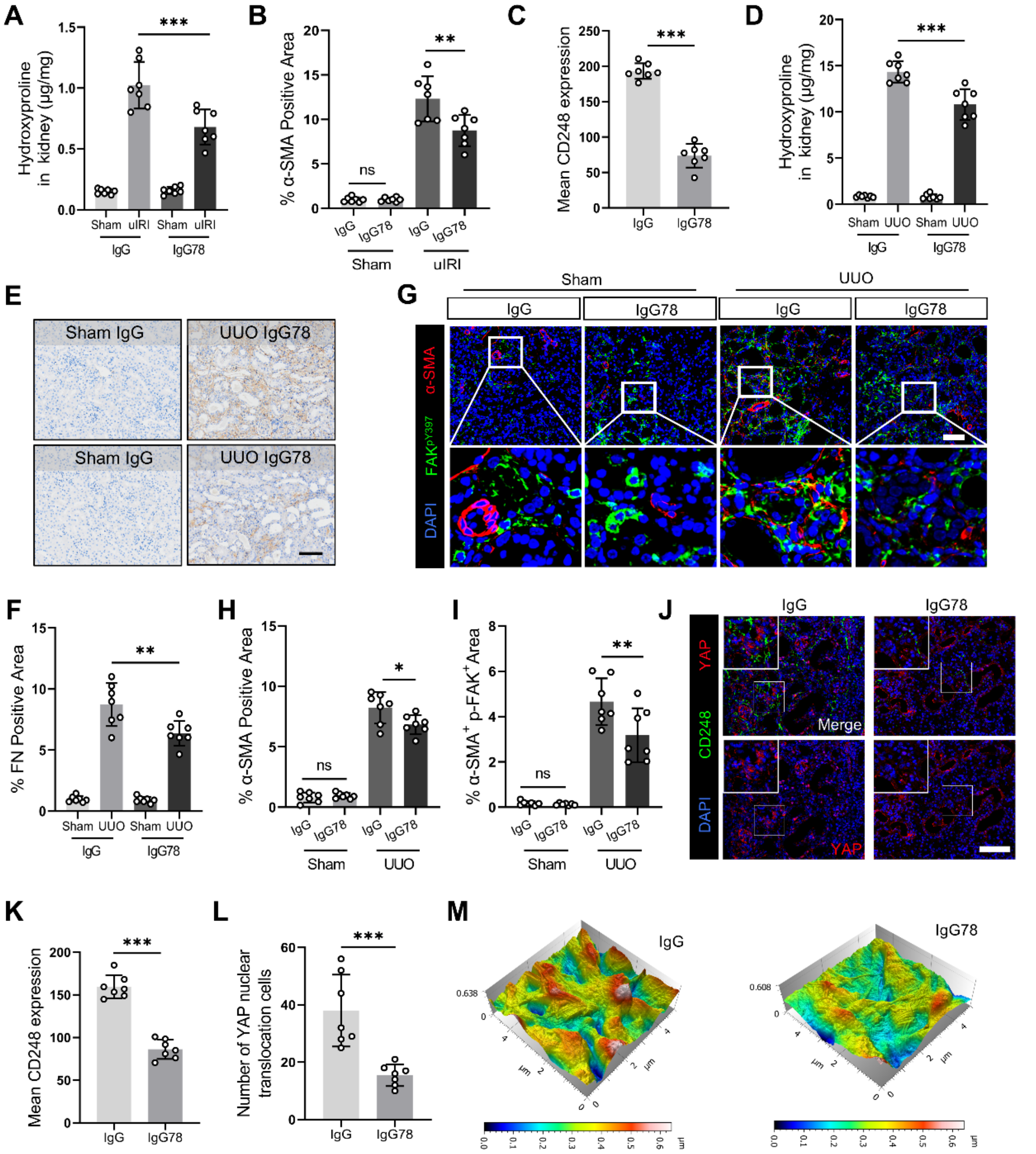

Figure S10. Monoclonal antibody IgG78 administration effectively attenuates ECM deposition *in vivo*, related to figure 8. (**A**) The hydroxyproline content in murine kidney tissues with uIRI (*n* = 7). (**B**) Quantification of positive area of α-SMA. (**C**) Quantification of mean expression of CD248 in uIRI kidneys. (**D**) The hydroxyproline content in murine kidney tissues with UUO (*n* = 7). (**E and F**)

Representative images of IHC staining of FN (n = 7). Scale bar, 50 μm and quantification of positive area of FN. (**G to I**) Representative immunofluorescence images of pY397-FAK and α-SMA in mice with UUO injury. Scale bar, 100 μm (G). Qualification of positive area of α-SMA (H), as well as α-SMA^+pY397-FAK^+ myofibroblasts (n = 7) (I). (**J to L**) Representative immunofluorescence images of CD248 and YAP in mice with UUO injury. Scale bar, 100 μm (J). Qualification of mean expression of CD248 (K), as well as $CD248^+$ myofibroblasts with YAP nuclear translocation (n = 7) (L). (**M**) AFM scanning reconstruction map of stromal matrix for kidney of UUO mice. *$P < 0.05$, **$P < 0.01$, ***$P < 0.001$. All data are represented as mean ± s.d from $n \geq 3$ independent experiments. Statistics were calculated using two-tailed, unpaired Student's t test or ANOVA with Tukey's test. Source data are provided as a Source Data file.

Figure S11

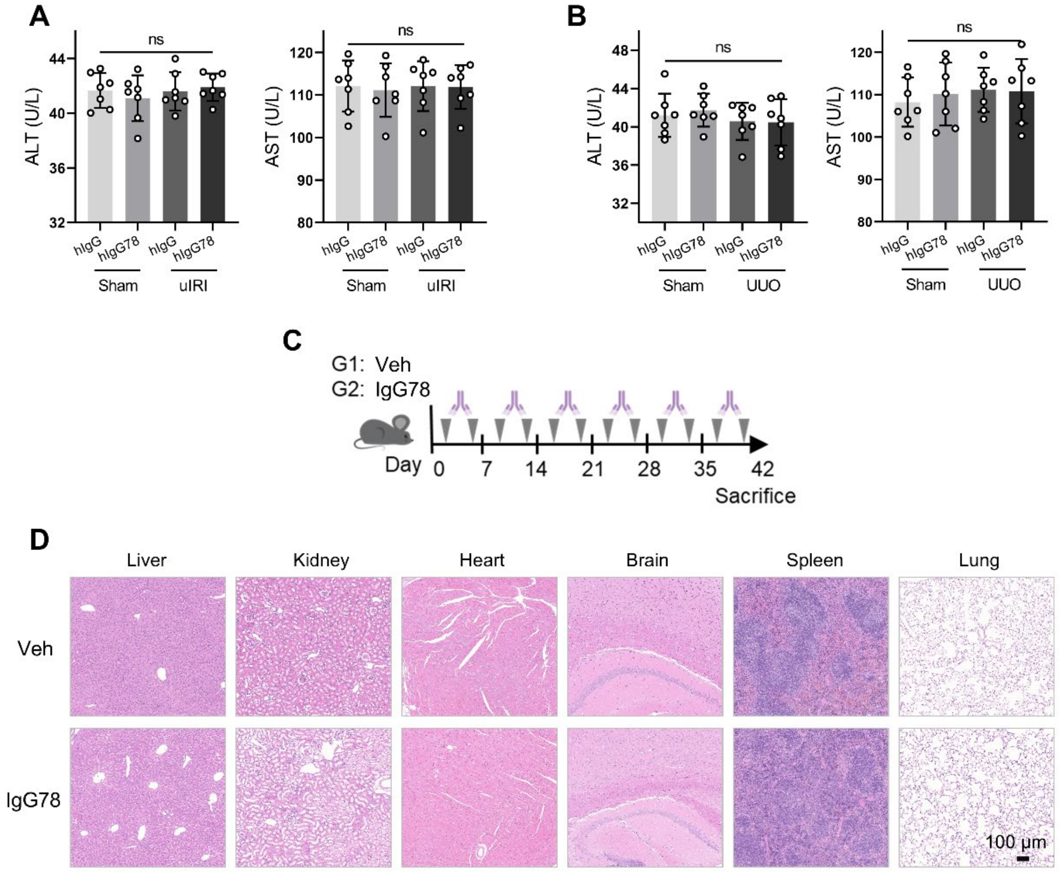

Figure S11. In vivo safety assessment of IgG78. (**A and B**) Liver function injury indicated by serum AST and ALT levels following uIRI (A) or UUO (B). (**C**) Schematic of safety assessment for IgG78 administration. (**D**) HE staining of important organs from mice with vehicle or IgG78 therapy treatment. Source data are provided as a Source Data file.
