# Supplementary material for: CD248 acts as a mechanosensory switch in fibroblast subsets to establish distinct pathological niches in renal fibrosis
Source: Nat Commun. 2026 May 6;17:7361. doi: 10.1038/s41467-026-72187-0 (PMC13402351; doi:10.1038/s41467-026-72187-0)
Supplement: Supplementary file 2 — Description of Additional Supplementary Files [file 41467_2026_72187_MOESM2_ESM.pdf]

Description of Additional Supplementary Files

Title: Supplementary Data 1

Description: Differentially expressed genes (DEGs) and annotation markers used to identify distinct fibroblast subpopulations. The table details average log2 fold changes, detection percentages, and adjusted p-values for each cluster. P values were determined using a two-sided Wilcoxon rank-sum test with Bonferroni correction for multiple comparisons.

Title: Supplementary Data 2

Description: Transcriptomic profiling and Gene Ontology (GO) enrichment analysis of the seven distinct fibroblast subsets. The table details enriched GO terms across biological processes, cellular components, and molecular functions, along with corresponding q-values, gene ratios, and associated gene symbols for each cluster.

Title: Supplementary Data 3

Description: Gene signatures characterizing each of the identified fibroblast subpopulations, including ECM-hi Fibs, ECM-med Fibs, CCL21/CCL19-hi Fibs, CCL2-hi Fibs, Actin Fibs, Metabol Fibs, and Resident Fibs.

Title: Supplementary Data 4

Description: Demographic and clinical profiles of the CKD patient cohort (n = 56) included in this study. The cohort comprises 36 males and 20 females, with a mean age of 47.3 years (range: 24–79 years). The table details baseline clinical parameters, including serum creatinine and estimated glomerular filtration rate (eGFR), alongside the corresponding histological scores assessed from human kidney biopsy sections.

Title: Supplementary Data 5

Description: List of differentially expressed membrane molecules (DEMMs) identified in CKD fibroblasts. The table provides specific gene symbols alongside their corresponding log2 fold change (logFC) values.

Title: Supplementary Data 6

Description: Correlation analysis evaluating the relationship between membrane molecules specifically expressed by ECM-hi fibroblasts and the core matrisome score across public CKD datasets (E-MTAB-2502 and GSE66494). Statistical values were calculated using two-sided Pearson/Spearman correlation analysis, providing correlation coefficients and adjusted p-values.

Title: Supplementary Data 7

Description: Differential gene expression analysis comparing CD248+ and CD248- fibroblast populations derived from scRNA-seq data. P values were determined using a two-sided Wilcoxon rank-sum test with Bonferroni correction for multiple comparisons.

Title: Supplementary Data 8

Description: Gene Set Enrichment Analysis (GSEA) results highlighting CD248-associated pathways. The analysis compares groups across three distinct datasets: UUO bulk RNA-seq (CD248 WT vs. CD248 -/-), scRNA-seq (CD248+ vs. CD248- fibroblasts), and the GSE66494 clinical cohort (CD248-high vs. CD248-low patients). Normalized Enrichment Scores (NES) and False Discovery Rates (FDR) are provided.

Title: Supplementary Data 9

Description: Results of the mass spectrometry analysis identifying CD248-interacting proteins. The data displays comparative protein abundances between Fibs-CD248 and control Fibs-IgG groups, including specific gene names, protein descriptions, and enrichment ratios.

Title: Supplementary Data 10

Description: Oligonucleotide sequences of the forward and reverse primers used for RT-qPCR experiments in this study.

Title: Supplementary Data 11

Description: Oligonucleotide sequences used for designing lentiviral shRNA constructs in this study. The table provides the specific target sequences utilized for the targeted knockdown of IQGAP1 and ARF6.
