# Supplementary material for: CD248 acts as a mechanosensory switch in fibroblast subsets to establish distinct pathological niches in renal fibrosis
Source: Nat Commun. 2026 May 6;17:7361. doi: 10.1038/s41467-026-72187-0 (PMC13402351; doi:10.1038/s41467-026-72187-0)
Supplement: Supplementary file 4 — Reporting Summary [file 41467_2026_72187_MOESM4_ESM.pdf]

nature portfolio

Corresponding author(s): *Double-anonymous peer review submissions: write DAPR and your manuscript number here instead of author names.*

Last updated by author(s): *YYYY-MM-DD*

Reporting Summary

Nature Portfolio wishes to improve the reproducibility of the work that we publish. This form provides structure for consistency and transparency in reporting. For further information on Nature Portfolio policies, see our Editorial Policies and the Editorial Policy Checklist.

Statistics

For all statistical analyses, confirm that the following items are present in the figure legend, table legend, main text, or Methods section.

n/a	Confirmed	
☐	☒	The exact sample size (*n*) for each experimental group/condition, given as a discrete number and unit of measurement
☐	☒	A statement on whether measurements were taken from distinct samples or whether the same sample was measured repeatedly
☐	☒	The statistical test(s) used AND whether they are one- or two-sided<br>*Only common tests should be described solely by name; describe more complex techniques in the Methods section.*
☐	☒	A description of all covariates tested
☐	☒	A description of any assumptions or corrections, such as tests of normality and adjustment for multiple comparisons
☐	☒	A full description of the statistical parameters including central tendency (e.g. means) or other basic estimates (e.g. regression coefficient) AND variation (e.g. standard deviation) or associated estimates of uncertainty (e.g. confidence intervals)
☐	☒	For null hypothesis testing, the test statistic (e.g. *F*, *t*, *r*) with confidence intervals, effect sizes, degrees of freedom and *P* value noted<br>*Give P values as exact values whenever suitable.*
☐	☒	For Bayesian analysis, information on the choice of priors and Markov chain Monte Carlo settings
☐	☒	For hierarchical and complex designs, identification of the appropriate level for tests and full reporting of outcomes
☐	☒	Estimates of effect sizes (e.g. Cohen's *d*, Pearson's *r*), indicating how they were calculated

Our web collection on statistics for biologists contains articles on many of the points above.

Software and code

Policy information about availability of computer code

Data collection	Microscopy images were collected using an Olympus VS200 system and Olympus FV3000 system confocal microscope software. Flow cytometry data were acquired using BD FACSDiva software (v8.0). Atomic Force Microscopy (AFM) data were collected using Keysight 5500 software. Western blot images were captured using Bio-Rad Image Lab (v6.0).
Data analysis	Statistical analysis and graphing were performed using GraphPad Prism (version 9.0). Flow cytometry data were analyzed using FlowJo (version 10.8). Image analysis was performed using ImageJ/Fiji (version 1.53). Single-cell RNA-seq data were processed using Cell Ranger (v6.1.2) and analyzed using R (v4.1.0) with Seurat (v4.3.0), Monocle (v2.22.0), SCENIC (v1.2.4), and clusterProfiler (v3.0.4). Bulk RNA-seq data were processed using Cutadapt (v1.9) and FastQC (v0.11.9). Custom scripts for bioinformatics analysis are available at https://github.com/RangerXc/CKD.

For manuscripts utilizing custom algorithms or software that are central to the research but not yet described in published literature, software must be made available to editors and reviewers. We strongly encourage code deposition in a community repository (e.g. GitHub). See the Nature Portfolio guidelines for submitting code & software for further information.

Data

Policy information about availability of data

All manuscripts must include a data availability statement. This statement should provide the following information, where applicable:

- Accession codes, unique identifiers, or web links for publicly available datasets
- A description of any restrictions on data availability
- For clinical datasets or third party data, please ensure that the statement adheres to our policy

All the expression data can be obtained from the public databases. ScRNA-seq data of CKD patients are available on the website (https://zenodo.org/record/4059315). For RNA-seq datasets of CKD patients, E-MTAB-2502 was downloaded from the Array Express database, and GSE66494 was downloaded from the gene expression omnibus (GEO) database. For mouse RNA-seq data, Processed and raw data are available via the GEO under the accessions GSE241634. Source data are provided with this paper. Custom scripts used in Single cell and bulk RNA-seq data analysis are available at: https://github.com/RangerXc/CKD.

Research involving human participants, their data, or biological material

Policy information about studies with human participants or human data. See also policy information about sex, gender (identity/presentation), and sexual orientation and race, ethnicity and racism.

Reporting on sex and gender	For the human kidney biopsy validation cohort, biological sex was determined based on clinical records. Both male and female samples were included (Male: n=36, Female: n=20). Sex-disaggregated analysis was not performed due to the limited sample size used for histological validation. For public datasets (scRNA-seq and bulk RNA-seq), sex information was utilized as provided in the original study metadata. Only male mice were used in this study to minimize the variability caused by the estrous cycle and because male mice exhibit more consistent and robust renal fibrosis in the UUO and uIRI models, consistent with standard practices in the field.
Reporting on race, ethnicity, or other socially relevant groupings	Race and ethnicity data were not collected for the archival human biopsy samples, as the study focuses on conserved molecular mechanisms of renal fibrosis and these variables were not considered covariates. For public datasets, demographic information was used as provided in the source repositories.
Population characteristics	Human kidney tissue samples were obtained from patients clinically diagnosed with Chronic Kidney Disease (CKD) (including Lupus Nephritis, Hypertensive Nephropathy, Diabetic Nephropathy, and Chronic Glomerulonephritis) and normal tissues. The validation cohort consisted of 56 patients. Detailed clinical characteristics for the public datasets (GSE66494, E-MTAB-2502) are available in their respective original publications.
Recruitment	Participants were not actively recruited for this study. Human kidney tissues were obtained retrospectively from archival samples collected during standard diagnostic biopsies at the Department of Nephrology, Xijing Hospital. Samples were selected based on confirmed pathological diagnosis of fibrosis and tissue availability. No self-selection bias was applicable.
Ethics oversight	This study was approved by the ethics committee of Xijing Hospital, Air Force Medical University (Xi'an, China), and all patient samples were collected with informed consent.

Note that full information on the approval of the study protocol must also be provided in the manuscript.

Field-specific reporting

Please select the one below that is the best fit for your research. If you are not sure, read the appropriate sections before making your selection.

☒ Life sciences ☐ Behavioural & social sciences ☐ Ecological, evolutionary & environmental sciences

For a reference copy of the document with all sections, see nature.com/documents/nr-reporting-summary-flat.pdf

Life sciences study design

All studies must disclose on these points even when the disclosure is negative.

Sample size	Sample sizes for animal experiments (typically n=5-7 per group) were chosen based on previous experience with similar renal fibrosis models (UUO and uIRI) and standard practices in the field to ensure adequate statistical power to detect significant differences in fibrosis metrics.
Data exclusions	For single-cell RNA-seq analysis, strict quality control criteria were applied as pre-established: cells with fewer than 200 detected genes, greater than 20% mitochondrial content were excluded. No data were excluded from the in vivo or in vitro functional analyses.
Replication	All in vitro experiments were performed at least three independent times with similar results. In vivo findings were verified in two independent mouse models (UUO and uIRI) and multiple cohorts. All attempts at replication were successful.
Randomization	For animal experiments, age- and sex-matched mice were randomly assigned to experimental groups. For in vitro studies, culture wells were randomly assigned to different treatment conditions to minimize potential batch effects.

Blinding	Investigators were blinded to group allocation during data collection and analysis, particularly for histological quantification and Atomic Force Microscopy measurements.

Reporting for specific materials, systems and methods

We require information from authors about some types of materials, experimental systems and methods used in many studies. Here, indicate whether each material, system or method listed is relevant to your study. If you are not sure if a list item applies to your research, read the appropriate section before selecting a response.

Materials & experimental systems

n/a	Involved in the study
☐	☒ Antibodies
☐	☒ Eukaryotic cell lines
☒	☐ Palaeontology and archaeology
☐	☒ Animals and other organisms
☒	☐ Clinical data
☒	☐ Dual use research of concern
☒	☐ Plants

Methods

n/a	Involved in the study
☒	☐ ChIP-seq
☐	☒ Flow cytometry
☒	☐ MRI-based neuroimaging

Antibodies

Antibodies used	HRP-labeled secondary antibodies (#ab6721, Abcam); anti-CD248 (#ab204914, Abcam); anti-ZEB1 (#303480, Abcam); anti-NF-κB1 (#AF3219, Affinity Bioscience); anti-Tenascin C (#ab108930, Abcam); anti-α-SMA (#14395-1-AP, Proteintech); anti-Fibronectin (#15613-1-AP, Proteintech); anti-COL1A1 (#E8F4L, CST); anti-αSMA (#14395-1-AP, Proteintech); anti-pY118Paxillin (#E9U9F, CST); anti-Fibronectin (#15613-1-AP, Proteintech); anti-YAP (#13584-1-AP, Proteintech); anti-p65 (#ab32536, Abcam); anti-DCN (#ab277636, Abcam); anti-POSTN (#ab215199, Abcam); anti-PDGFRB (#ab69506, Abcam); anti-CD3 (#ab16669, Abcam); anti-CCR7 (#ab32524, Abcam); anti-CD68 (#25747-1-AP, Proteintech); anti-CCR2 (#ab273050, Abcam); F-actin (#p1951, Sigma-Aldrich); Anti-EpCAM (#118214, BioLegend), anti-CD45 (#157212, BioLegend), and anti-CD31 (#160204, BioLegend); anti-CD248 (#sc-377221, Santa Cruz Biotechnology); anti-CTGF (#25474-1-AP, Proteintech); anti-FAK (phospho Y397) (#ab81298, Abcam); anti-FAK (#3285, CST); anti-Paxillin (phospho Y118) (#ab32084, CST); anti-Paxillin (#50195, CST); anti-GAPDH (#10494-1-AP, Proteintech); anti-FLAG (#20543-1-AP, Proteintech);
Validation	All antibodies were validated for the species and applications used in this study. Validation statements for each antibody are provided on the manufacturers' websites. Specifically, antibodies were validated by the manufacturers using genetic or biological controls. Flow cytometry antibodies from BioLegend (EpCAM, CD45, CD31) were validated for specificity on relevant cell populations. In this study, specificity was further confirmed using isotype controls, secondary-only controls, and by observing expected molecular weights and localization patterns.

Eukaryotic cell lines

Policy information about cell lines and Sex and Gender in Research

Cell line source(s)	HFL1, obtained from ATCC
Authentication	Cell line identity was confirmed by STR profiling
Mycoplasma contamination	Cells were routinely tested for mycoplasma contamination and were negative on 2 weeks prior to experiments.
Commonly misidentified lines (See ICLAC register)	No. HFL1 is not listed as commonly misidentified in the ICLAC register

Animals and other research organisms

Policy information about studies involving animals; ARRIVE guidelines recommended for reporting animal research, and Sex and Gender in Research

Laboratory animals	Male C57BL/6J mice, aged 8-10 weeks, were used for all experiments. Wile type animals were obtained from GemPharmatech Co., Ltd. For genetic mice lines, all mice were of the C57BL/6J background. Cd248CreERT mice, Rosa26LSL-TdTomato-DTR mice, Co1la2CreERT mice and Cd248fl/fl mice were generated, and purchased from Shanghai Model Organisms Center, Inc. Cd248-/- mice, Cd248CreERT mice, and Yap1 fl/fl mice(34) were also obtained as described previously. Mice were housed in specific pathogen-free (SPF) conditions with a 12-hour light/dark cycle and free access to food and water. Ambient temperature was maintained at 22±2°C with 40-60% humidity.
Wild animals	The study did not involve wild animals.
Reporting on sex	Only male mice were used in this study. This decision was based on established literature indicating that male mice develop more

Reporting on sex	robust and consistent renal fibrosis in the UUO and uIRI models compared to females, which are partially protected by estrogen. Excluding females minimizes variability associated with the estrous cycle, allowing for a clearer assessment of the specific molecular mechanism driven by CD248. Findings are interpreted in the context of male renal fibrosis.
Field-collected samples	The study did not involve samples collected from the field.
Ethics oversight	The animal experiments conducted in this study were conducted in accordance with the Guidelines for the Care and Use of Laboratory Animals of Air Force Military Medical University.

Note that full information on the approval of the study protocol must also be provided in the manuscript.

Plants

Seed stocks	NA
Novel plant genotypes	NA
Authentication	NA

Flow Cytometry

Plots

Confirm that:

☒ The axis labels state the marker and fluorochrome used (e.g. CD4-FITC).

☒ The axis scales are clearly visible. Include numbers along axes only for bottom left plot of group (a 'group' is an analysis of identical markers).

☒ All plots are contour plots with outliers or pseudocolor plots.

☒ A numerical value for number of cells or percentage (with statistics) is provided.

Methodology

Sample preparation	Fibrotic kidneys were harvested and minced with scissors, then enzymatically digested in CO2-independent incubator shaker (#ISF1-XC, Kuhner, Germany) with 1mg/ml Collagenase I (#SCR103, Sigma-Aldrich) and IV mixture (#C4-28-100MG, Sigma-Aldrich) for 1h at 37°C under 80 rpm agitation. After diluted with serum-free medium and centrifuged at 200g for 10min, the cell pellets were resuspended in ACK lysis buffer (#NC9067514, ThermoFisher) to remove blood cells. Prior to staining, the cell suspended in FACS buffer comprised of PBS with 5% BSA (#SRE0096, Sigma-Aldrich) was filtered through a 40 µm mesh (#BS-40-XBS, Biosharp).
Instrument	Flow cytometry data were acquired on a BD LSRFortessa flow cytometer.
Software	Data were collected using BD FACSDiva and analyzed using FlowJo software.
Cell population abundance	live cells were processed to remove epithelial, immune, and endothelial cells using EpCAM, CD45, and CD31 markers, respectively. Subsequently, triple-negative cells were sorted, and TdTomato was employed to categorize fibroblasts into CD248-negative and CD248-positive populations.
Gating strategy	Live, single cells were sorted based on negative selection for EpCAM, CD45, and CD31 (Lineage-negative) and positive selection for CD248 (tdTomato+). CD248+ fibroblasts constituted approximately 16.43% of the total lineage-negative stromal population prior to sorting.

☒ Tick this box to confirm that a figure exemplifying the gating strategy is provided in the Supplementary Information.
