# Supplementary material for: CD248 acts as a mechanosensory switch in fibroblast subsets to establish distinct pathological niches in renal fibrosis
Source: Nat Commun. 2026 May 6;17:7361. doi: 10.1038/s41467-026-72187-0 (PMC13402351; doi:10.1038/s41467-026-72187-0)
Supplement: Supplementary file 5 — Transparent Peer Review file [file 41467_2026_72187_MOESM5_ESM.pdf]

nature portfolio

Peer Review File

CD248 acts as a mechanosensory switch in fibroblast subsets to establish distinct pathological niches in renal fibrosis

Corresponding Author: Professor Weijun Qin

This file contains all reviewer reports in order by version, followed by all author rebuttals in order by version.

Version 0:

Reviewer comments:

Reviewer #1

(Remarks to the Author)
The authors conducted gene expression analyses of kidney fibrotic tissues from patients with diabetic kidney disease (DKD) and identified various fibroblast subpopulations associated with renal fibrosis and inflammation. In subsequent analyses, the membrane receptor CD248 was found to be predominantly expressed in the ECM-associated fibroblast subpopulation. They demonstrate that in response to disordered ECM, CD248, through its ligand-binding domain, acts as the driver of a pro-fibrotic biomechanical feedback loop, leading to the activation of myofibroblasts and renal fibrosis. Mechanistically, CD248 interacts with the scaffold protein IQGAP1, which promotes downstream FAK/Paxillin-YAP signaling and stromal remodeling. These results were validated using mouse models, specifically in the unilateral ischemia-reperfusion injury (uIRI) and unilateral ureteral obstruction (UUO) models, to assess functional implications. The authors have also demonstrated the potential benefits of therapeutic approaches targeting CD248 to inhibit renal fibrosis in these models.

The studies are clearly presented and reveal relevant and novel insights by which CD248 promotes signaling pathways that trigger myofibroblast activation and organ fibrosis. My recommendations are as follows:

1) CD248 is recognized as a marker for pericytes. In Figure S1, it is illustrated that pericytes and fibroblasts cluster closely together, suggesting a potential overlap between these cell types. The authors need to clarify whether the observed results are specific to fibroblasts. On line 136, it is essential to describe the methods used to exclude pericytes from the re-clustering analysis. In Figure S2A, the highlighted rectangle encompasses both cell types—pericytes and fibroblasts. Table S5 indicates that PDGFRB is highly co-expressed with the ECMhi-Fibs signature, implying that pericytes might be included in the ECMhi-Fibs cluster. This overlap raises questions about the specificity of the findings to fibroblasts and needs to be addressed to validate the conclusions drawn.

2) There appears to be an inconsistency in the description of the spatial distribution results (Line 162-166). Specifically, the authors claim that POSTN is specific to Cluster 1, yet the data indicate that POSTN is expressed by both Cluster 0 and Cluster 1. Similarly, CCL19 is described as specific to Cluster 4, but is shown to be expressed by Clusters 3 and 2 as well. This section of the manuscript needs a thorough revision to accurately reflect the gene expression data across the clusters. Additionally, it would be beneficial to include detailed gene expression data for each cluster as supplementary material. Furthermore, the analysis should address whether pericytes are included in these clusters, as their presence could influence the specificity of the gene expression profiles attributed to fibroblasts.

3) The authors assert that i-Fibs dominate in areas with significant immune cell infiltration. To substantiate this claim, it is crucial to provide empirical data demonstrating the prevalence of i-Fibs in these regions. Detailed quantitative analyses, possibly including spatial mapping and correlation studies between i-Fibs and immune cell markers, should be presented to clarify this observation.

4) The manuscript is divided into two sections: the first part demonstrates fibroblast heterogeneity by showing that CD248 is specifically expressed in a subset of fibroblasts designated as ECMhi-Fibs. The second part investigates the role of CD248 in renal fibrosis induced by unilateral ureteral obstruction (UUO) and unilateral ischemia-reperfusion injury (uIRI), using various genetic mouse models, including CD248 knockout (KO) mice and conditional CD248 KO mice (Col1a2-CreERT

mice). It is imperative that the authors provide a rationale for not employing a knockout strategy specific to ECMhi-Fibs. This explanation is crucial, especially given the specific expression of CD248 in this subpopulation, as a targeted knockout could potentially yield more direct insights into the functional role of CD248 within these particular cells.

5) The study describes the engineering of two truncated forms of CD248: CD248 ΔECD, which lacks the extracellular domain (ECD), and CD248 ΔCTLD, which lacks the C-type lectin domain (CTLD). It is essential to include detailed methodology for the engineering and validation of these truncated proteins in the Methods section of the manuscript. Specifically, the procedures for constructing these variants, the expression systems used, and the validation assays to confirm their structural integrity and functional activity should be shown.

Minor:
Line 97: Monocle antibodies (mAbs) should be monoclonal antibodies (mAbs).
Line 150: Please specify what is meant by the term corresponding controls in that context.
Line 151: The heatmap does not show me an increase in ECM fibs or i-fibs. Please explain what is shown on the heatmap.
Line 176: The term "renal hypofunction" should be replaced with "renal dysfunction" or "renal insufficiency".
Line 184-186: Which gene clusters are the authors referring to? Are they the same clusters described in line 140 or are they specific clusters in ECMhi-Fibs?
Line 197: The authors should provide information why they only focus on ECMhi-Fibs (cluster 0).
Line 209: The list of genes that are diffenrently expressed between CD248+ and CD248- pericytes should be provided as supplementary table.
Line 213-216: The authors should refer to the respective figure showing multiplex immunohistochemical (mIHC) staining.
Line 217: Information about the in-house CKD cohort should be provided and explianed in the materials and methods section.
Line 297: It should be specified which tissue was used for these analyses. Whole kidney lysates?
Line 301: CRISPR-Cas9-induced CD248 knockout in primary fibroblasts resulted in a decrease in pro-fibrotic ECM molecules as shown in Fig.5B. However, Fig.5B shows increased expression of Col1a2. The authors should comment on that result.
Line 305: The authors should refer to the respective figure.
Line 350: There is a "," that needs to be deleted.
Line 453-456: There are no data in the manuscript to show that CCL19&21 hi-fibs recruit immune cells. This should be stated as a hypothesis by the authors.
Line 494: The results highlight the role of the CTLD of CD248.
Line 1081: Figure 3H shows no statistical calculations.
Line 1152: Please indicate which tissue was analysed. Which tumour tissue was used here?
Line 1196-1200: Please rephrase, do not parenthesise the different groups.
Figure 1B: The presented plots are not vulcano plots. In a vulcano plot the X-axis represents the logarithm of the fold change between conditions.
Figure 1E: POSTN-expressing cells are not detectable.
Figure 1I: The KEGG enrichment is shown in Cluster2. It should be explained which cells Cluster2 refers to. In addition, the figure legend mentions Cluster 1, but Cluster 2 is shown in Fig1I.
Figure 2B: The labelling of the figures must be explained in the legend (E-MTAB-2502...).
Figure 2G-H: The labeling of the figures must to be explained. What is meant by “In-house data”?
Figure 3 H-O: The wild type control with uIRI is missing.
Figure 5C: The Venn diagram should include all numbers of KEGG pathways. Not only the overlap.
Figure 5D: Why there are 2 bands visible for CD248?
Figure 7B: Since both lines have a "+" in the same column, the panel indicates that CD248-pos and CD248-KO have been grown together. Please correct.
Figure 7H, I, J: Why there are 2 bands visible for CD248 in Figure 7H and 7I but not in Figure 7J?
Figure legends: The figure legends incorrectly use the term "qualification". It should be "quantification", e.g. line 1040, 1071, 1074, 1076, 1128 and many others.
Table S2: In which cluster CD248 is increased?
Figure S3A,B: The labeling of the figures need to be explained in the legend (E-MTAB-2502…)
The font size in the figures needs to be adjusted: for example, Figure S1 D and E. The small font size in the figures is difficult to read.

Reviewer #2

(Remarks to the Author)
The manuscript titled "CD248 drives an ECM-myofibroblast feedback loop, establishing aberrant biomechanical niche to promote renal fibrosis" analyzed in renal fibrosis the role of CD248 in the progression of renal fibrosis. The study employs genetically engineered mouse models, and therapeutic antibody interventions, which have been published in part previously. The findings present CD248 as a potential promising therapeutic target for tissue renal fibrogenesis.
While the study makes use of several transgenic mouse models to study the role of CD248 in fibrosis I have substantial problems with the novelty of the pathomechanisms and approach in this manuscript.
That CD248 or Endosialin has a role in fibrogenesis in cancer and other disease setting is not novel at all, it is an “old hat”. A humanized monoclonal anti-CD248 antibody, ontuxizumab, is in clinical trials and has been discussed for other fibrotic human disease settings for almost a decade now (PMID: 26673786).
While the manuscript ambitiously aims to present CD248 as a critical mediator in kidney fibrosis and a promising therapeutic

target, significant methodological and conceptual flaws undermine the validity and impact of the findings. Below, I outline key concerns regarding the study's design, data interpretation, and overall conclusions.
- The role of CD248 in stromal remodeling and fibrosis has been explored extensively in other organ systems, such as cancer-associated fibroblasts (CAFs) in tumors. The extrapolation of these findings to kidney fibrosis lacks sufficient novelty to justify the claims of a breakthrough yet the discovery of a novel pathomechanism linked to mechanobiology.
- The molecular pathway involving CD248, IQGAP1, and ARF6·GTP is described as novel; however, similar mechanotransduction pathways involving focal adhesions and YAP signaling are well-documented (PMID: 31040262). The study fails to clarify how the CD248 pathway is distinct from already-established mechanisms.
- The use of mouse models such as unilateral ureteral obstruction (UUO) and ischemia-reperfusion injury (uIRI) provides limited relevance to the human context. These models fail to replicate the complexity of chronic kidney disease (CKD) progression, especially the contributions of inflammation, comorbidities, and systemic factors seen in human patients. As such it remains unclear how similar the CD248 population in human vs. mouse are. The stainings shown in Figure 3a-d suggest that CD248 fibroblasts/myofibroblasts are localized in the entire interstitium of the mouse kidney, yet in the human tissue this is not the case in fibrosis. Single-cell RNA sequencing of those tdtom positive cells would be necessary and a comparison to tdtom- PDGFRbeta cells to explore the identify of these cells.
- The authors do not adequately address the differences in CD248 expression and fibroblast phenotypes between human CKD and the mouse models. Given these discrepancies, the translational potential of the findings is questionable.
- Although the single-cell RNA sequencing (scRNA-seq) data is central to identifying CD248+ fibroblasts, the analysis is not sufficiently rigorous:
- The clustering of fibroblast subpopulations lacks validation using independent datasets. Particullary it is well known that RNA and protein expression do not necessarily correlate. As the CD248 protein expression is central to the paper, I would suggest to repeat single cell RNA sequencing of human fibroblasts from kidney and adding CD248 oligolabeled antibodies for CITE-seq to gain deeper insights which fibroblasts express CD248 on their surface. Also for the other mural cells this would be important including pericyte populations.
- Key markers used to define the ECMhi and i-Fibs subpopulations are poorly justified, with limited evidence supporting their specificity for fibrosis-driving cells.
- The transition of fibroblasts from inflammatory (i-Fibs) to ECM-producing (ECMhi) phenotypes is based solely on trajectory analysis, which is speculative and not experimentally validated. This would have to be shown using e.g. RNAscope or spatial transcriptomics.
- The monoclonal antibody IgG78 is described as a promising therapeutic intervention; however:
- The lack of pharmacokinetic and toxicity data in the in vivo models raises concerns about its safety and feasibility for clinical translation.
- The effectiveness of IgG78 is only tested in two mouse models, both of which artificially induce fibrosis. Its impact on more complex or spontaneous CKD models is not addressed.
- The authors fail to discuss potential compensatory mechanisms or the redundancy of mechanotransduction pathways that could limit the efficacy of CD248 targeting.
- The interactions between CD248+ fibroblasts and other cell types in the niche are not explored, leaving a significant gap in understanding how CD248 contributes to the broader fibrotic process.
- The authors make bold claims about the therapeutic potential of targeting CD248 without sufficiently addressing limitations. For instance, while CD248 knockout or depletion reduces fibrosis, it is unclear whether this effect translates into functional improvements in kidney health or protection against CKD progression.
- The reliance on α-SMA as a myofibroblast marker, despite acknowledging its limitations, undermines the specificity of the conclusions drawn about the role of CD248+ fibroblasts.
The manuscript's attempt to position CD248 as a novel therapeutic target for kidney fibrosis falls short due to insufficient novelty, incomplete validation of findings, and a lack of robust translational evidence. While the study incorporates cutting-edge techniques, its reliance on simplistic models and overstated conclusions limits its overall contribution to the field. Future work should focus on addressing these limitations, particularly by validating findings in human samples and expanding the scope to include the broader fibrogenic niche.

Reviewer #3

(Remarks to the Author)
This manuscript provides a comprehensive and innovative exploration of the role of CD248 in renal fibrosis. The authors have identified CD248 as a critical mechanotransduction mediator in fibroblast activation through the IQGAP1-ARF6-GTP pathway, bridging aberrant ECM properties and myofibroblast-driven fibrogenesis. By combining single-cell RNA sequencing, genetic mouse models, and bioinformatics analysis, the study demonstrates the central role of CD248 in sustaining the biomechanical feedback loop within the fibrotic niche. Furthermore, the development and functional validation of a CD248-targeting monoclonal antibody, IgG78, adds substantial therapeutic potential to the findings.
The manuscript is well-written, methodologically rigorous, and aligns with Nature Communications. The work has significant implications for understanding fibrotic disease mechanisms and developing targeted therapeutics. However, there are some issues listed below.

1. It is suggested to measure changes in ECM stiffness in healthy, CKD and CD248 deficient kidneys, and provide additional analyses on CD248's mechanosensory function under varying ECM stiffness levels in vitro to strengthen its role as an ECM sensor.
2. More evidence is needed on the safety of IgG78 therapy, focusing on its potential immunogenicity and effects on critical organs.
3. Although the effects of CD248 and IgG78 have been validated in animal models, systematic validation in human tissue

samples is lacking. Validation experiments of IgG78 in vitro human cell lines or organoids can be added, and associations between CD248 expression levels and clinical indicators such as kidney function in samples from CKD patients can be systematically analyzed.
4. In Figure 8, fibrosis-related markers like α-SMA, YAP, and renal function such as the estimated glomerular filtration rate after IgG78 treatment should be characterized.
5. A more intuitive schematic of the molecular mechanism of CD248 or the whole story would be better.

And some other minor issues:
6. Figure 1A and Supplemental Figure 9A do not seem to be mentioned in the text.
7. In Figure 2E, when EPCAM first appeared, what did it stand for.
8. The zoom image in the picture also needs a scale bar.
9. It is suggested to put the more intuitive staining data in the main figures, and put its statistical analysis data in the supplemental figures.
10. Should the figure 1I caption refer to gene cluster 2?
11. The label of FAK, T-FAK; Paxillin, T-Paxillin in the Western blot should be unified.

Together, this manuscript identified CD248 as a pivotal player in ECM-myofibroblast interactions and the therapeutic validation of IgG78 are of high scientific and clinical relevance.
This paper can be accepted with minor revisions. Addressing the outlined suggestions will further enhance the manuscript's impact and translational potential.

Reviewer #4

(Remarks to the Author)
I co-reviewed this manuscript with one of the reviewers who provided the listed reports. This is part of the Nature Communications initiative to facilitate training in peer review and to provide appropriate recognition for Early Career Researchers who co-review manuscripts.

Version 1:

Reviewer comments:

Reviewer #1

(Remarks to the Author)
I have no further major concerns and am satisfied with the revisions. All points raised during the initial review have been satisfactorily addressed.

Importantly, the authors have incorporated substantial new data that significantly strengthen the manuscript and further support the central conclusions. The additional experiments and analyses provide a more comprehensive mechanistic understanding of the role of CD248 in ECM-driven fibroblast activation and renal fibrosis, and they enhance the overall robustness and impact of the study.

The revised manuscript is clearly written, well structured, and the new data convincingly reinforce the proposed pro-fibrotic signaling axis involving CD248. Overall, the revisions have improved the quality of the work, and I believe that the manuscript now represents a strong and compelling contribution to the field.

Reviewer #2

(Remarks to the Author)
All my comments have been addressed.

Reviewer #3

(Remarks to the Author)
The authors have revised the manuscript and addressed most of the previous concerns. The overall clarity and presentation of the study have improved. The addition of the schematic model and reorganization of several figures have strengthened the narrative of the manuscript.
However, several issues still require clarification to further improve the rigor and completeness of the work.

1. The proposed positive feedback loop involving matrix stiffening and CD248 activation is conceptually compelling. The authors may consider clarifying whether direct experimental evidence supports stiffness-induced CD248 upregulation, or whether this remains a model-based interpretation.
2. The therapeutic mechanism of IgG78 could be briefly discussed in more detail, particularly whether the antibody primarily blocks ECM binding or modulates receptor signaling/clustering.
3. The causal relationship between CD248 and IQGAP1-ARF6 signaling would benefit from future genetic epistasis validation, which could be acknowledged as a limitation.
4. Figure S8 E needs to include fluorescence intensity data. Currently, the differences appear to be insignificant.

The manuscript is substantially improved and presents interesting findings. Addressing the points above would further enhance the mechanistic rigor and clarity of the study.

Reviewer #4

(Remarks to the Author)
I co-reviewed this manuscript with one of the reviewers who provided the listed reports. This is part of the Nature Communications initiative to facilitate training in peer review and to provide appropriate recognition for Early Career Researchers who co-review manuscripts.

Open Access This Peer Review File is licensed under a Creative Commons Attribution 4.0 International License, which permits use, sharing, adaptation, distribution and reproduction in any medium or format, as long as you give appropriate credit to the original author(s) and the source, provide a link to the Creative Commons license, and indicate if changes were made.
In cases where reviewers are anonymous, credit should be given to 'Anonymous Referee' and the source.
The images or other third party material in this Peer Review File are included in the article's Creative Commons license, unless indicated otherwise in a credit line to the material. If material is not included in the article's Creative Commons license and your intended use is not permitted by statutory regulation or exceeds the permitted use, you will need to obtain permission directly from the copyright holder.
To view a copy of this license, visit https://creativecommons.org/licenses/by/4.0/

Responses to Reviewers' Comments for Manuscript NCOMMS-24-83524

Point-by-point reply

Reviewer #1

The authors conducted gene expression analyses of kidney fibrotic tissues from patients with diabetic kidney disease (DKD) and identified various fibroblast subpopulations associated with renal fibrosis and inflammation. In subsequent analyses, the membrane receptor CD248 was found to be predominantly expressed in the ECM-associated fibroblast subpopulation. They demonstrate that in response to disordered ECM, CD248, through its ligand-binding domain, acts as the driver of a pro-fibrotic biomechanical feedback loop, leading to the activation of myofibroblasts and renal fibrosis. Mechanistically, CD248 interacts with the scaffold protein IQGAP1, which promotes downstream FAK/Paxillin-YAP signaling and stromal remodeling. These results were validated using mouse models, specifically in the unilateral ischemia-reperfusion injury (uIRI) and unilateral ureteral obstruction (UUO) models, to assess functional implications. The authors have also demonstrated the potential benefits of therapeutic approaches targeting CD248 to inhibit renal fibrosis in these models.

The studies are clearly presented and reveal relevant and novel insights by which CD248 promotes signaling pathways that trigger myofibroblast activation and organ fibrosis. My recommendations are as follows:

Major question:

[1] Reviewer 1' Comment: CD248 is recognized as a marker for pericytes. In Figure S1, it is illustrated that pericytes and fibroblasts cluster closely together, suggesting a potential overlap between these cell types. The authors need to clarify whether the observed results are specific to fibroblasts. On line 136, it is essential to describe the methods used to exclude pericytes from the re-clustering analysis. In Figure S2A, the highlighted rectangle encompasses both cell types—pericytes and fibroblasts. Table S5 indicates that PDGFRB is highly co-expressed with the ECMhi-

Fibs signature, implying that pericytes might be included in the ECMhi-Fibs cluster. This overlap raises questions about the specificity of the findings to fibroblasts and needs to be addressed to validate the conclusions drawn.

Authors' Response: We thank you for this constructive and insightful comment. We agree that the specific distinction between fibroblasts and pericytes within the stromal cell compartment is a critical point that requires careful clarification. We apologize that our original manuscript did not sufficiently detail the methods used to ensure the specificity of our fibroblast analyses. Our findings are indeed specific to fibroblasts, and we have now extensively revised the manuscript to address this. Specifically, we have implemented the following clarifications and revisions:

1. Marker-based cell type identification and exclusion of pericytes: at the ScRNA -seq analysis data, we employed a marker-based approach for cell annotation. Fibroblasts were specifically identified by the canonical markers *DCN*, *COL1A2*, and *LUM*, whereas pericytes were identified by the specific marker *RGS5*. Furthermore, although both are stromal cell types, our dimensionality reduction and clustering analyses (UMAP) demonstrated that fibroblasts and pericytes form distinct and non-overlapping clusters. This clear separation is visualized in the revised Figure S1A-C.

Figure S1A-C (*Highlighted in red boxes*)

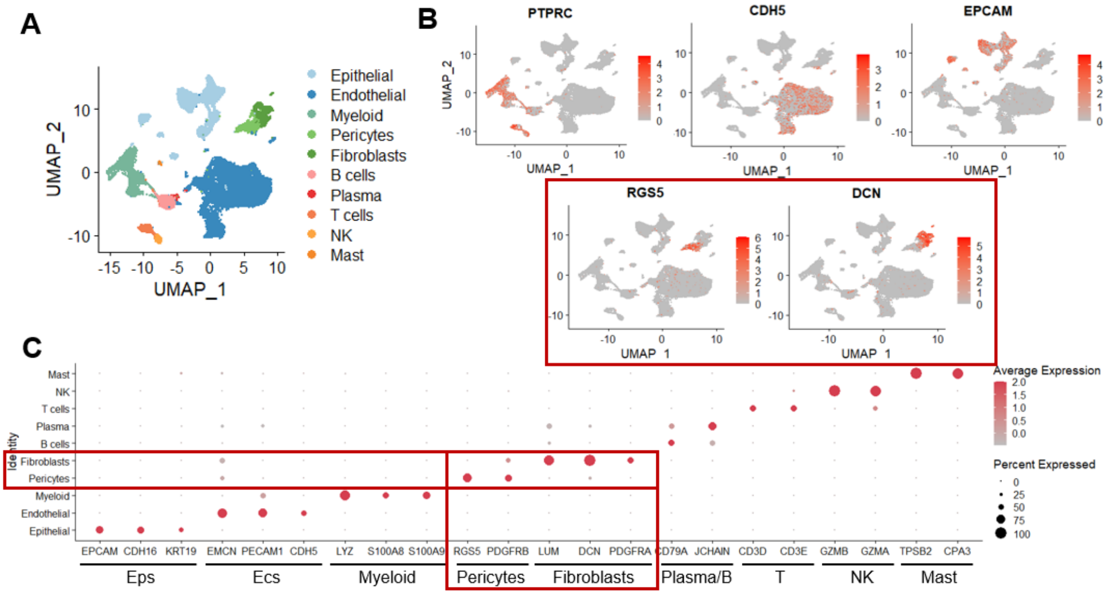

2. Correction of Figure S3A (formerly **Figure S2A**): We are grateful to the reviewer for this keen observation. We have corrected the highlighted rectangle in the figure, which is now Figure S3A, to specifically and accurately encompass only the fibroblast cluster, excluding the adjacent pericyte population.

Previous

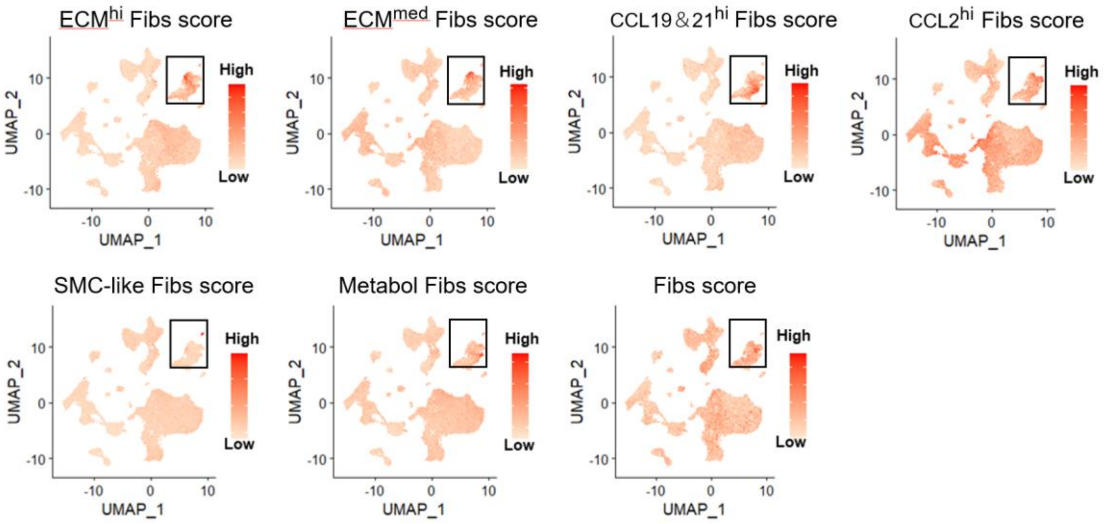

Revised

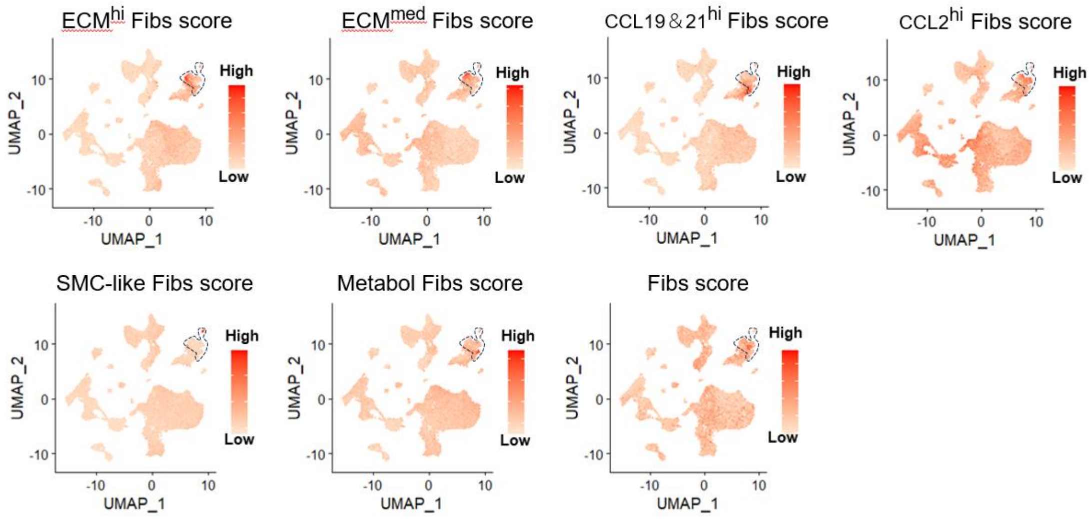

3. Clarification on PDGFRB expression and the ECMhi-Fibs signature: The reviewer correctly points out that PDGFRB is highly expressed in pericytes. However, it is also well-documented in the literature that PDGFRB expression is significantly upregulated in activated fibroblasts, rendering it a non-specific marker for distinguishing between these two cell types, particularly in a pathological context.

Our study addresses this potential ambiguity in two crucial ways. First, the ECMhi-Fibs cluster was identified from a cell population where pericytes had already been rigorously excluded based on the RGS5 marker, as detailed above. Therefore, the PDGFRB expression observed in this cluster originates specifically from activated fibroblasts. Second, as shown in Table S5, the gene signature of the ECMhi-Fibs cluster is highly enriched for a panel of well-established fibroblast activation markers, including CD248, PDGFRA, MRC2, DDR2, and CDH11. The co-expression of PDGFRB with this robust signature of fibroblast activation provides strong evidence that its presence is linked to the activated fibroblast phenotype, rather than contamination from pericytes.

In summary, the specificity of our fibroblast populations is foundational to our study. We have taken rigorous steps in our analysis pipeline to exclude potential confounding effects from pericytes and have now clarified these procedures in the manuscript to ensure the validity and accuracy of our conclusions. We thank you again for prompting these important improvements.

(*See the line 135-138. The first paragraph of the Results part "Identification of phenotypic heterogeneity of fibroblasts in the fibrogenic niche after kidney injury": To decipher the fibroblast heterogeneity in the fibrogenic niche, we firstly used canonical markers (RGS5 and PDGFRB for pericytes, DCN, LUM, and COL1A2 for fibroblasts) to distinguish fibroblasts from mesenchymal cells.*)

[2] Reviewer 1' Comment: There appears to be an inconsistency in the description of the spatial distribution results (Line 162-166). Specifically, the authors claim that POSTN is specific to Cluster 1, yet the data indicate that POSTN is expressed by both Cluster 0 and Cluster 1. Similarly, CCL19 is described as specific to Cluster 4, but is shown to be expressed by Clusters 3 and 2 as well. This section of the manuscript needs a thorough revision to accurately reflect the gene expression data across the clusters. Additionally, it would be beneficial to include detailed gene expression data for each cluster as supplementary material. Furthermore, the analysis should address whether pericytes are included in these clusters, as their presence could influence the specificity of the gene expression profiles attributed to fibroblasts.

Authors' Response: We sincerely thank you for this meticulous and valuable critique. You are correct; our previous description of the spatial distribution results (Lines 162-166) was inaccurate and contained errors that required correction. We have addressed each of your points as follows:

1. Regarding marker specificity and rationale: We have revised the manuscript to clarify the basis for our marker selection.

- Our choice of POSTN as a key marker for ECMhi-Fibs (Cluster 1) is based on two main criteria: ①it exhibits the most significant average expression level in this cluster compared to all others (Figure 1C), and ②its use is consistent with high-impact literature where it serves as a reliable marker for myofibroblast subpopulations (*1*).
- Furthermore, we sincerely apologize for a critical typographical error in the original text regarding the immune-related clusters. The correct assignment, which our data supports, is that CCL19 marks Cluster 2 and CCL2 marks Cluster 3 (not Cluster 4 as previously stated). This selection is based on their markedly higher and more specific expression in these respective clusters (Figure 1C). We

have thoroughly revised this section in the manuscript to accurately reflect the data.

2. Regarding supplementary gene expression data: We have now included a comprehensive table detailing the differentially expressed genes (DEGs) for each fibroblast cluster as Supplementary Table S1. This unbiased gene list further strengthens the characterization of each subpopulation and enhances the transparency of our findings.

3. Regarding pericyte exclusion: We wish to clarify that pericytes were rigorously excluded from our fibroblast dataset from the outset. During the initial bioinformatic filtering, we removed all cells expressing canonical pericyte markers, primarily RGS5 and PDGFRB. Therefore, the gene expression profiles and conclusions presented are indeed specific to the fibroblast lineage.
(*See the line 158-163. The one paragraph of the Results part "Fibroblasts establish functionally distinct and mutually exclusive pathological niches in injured kidneys": To characterize the spatial distribution of distinct fibroblast subsets during renal fibrotic progression, we performed RNAscope on serial sections of fibrotic renal tissue. Probes for DCN, POSTN, CCL19, and CCL2 were used to identify total fibroblasts, ECM-Fibs (POSTN), and two major i-Fib subsets: C2 (CCL19) and C3 (CCL2), respectively. Compared to normal kidney tissue, the abundance of ECM-Fibs, C2, and C3 subsets was markedly increased in fibrotic areas (Figure 1, D to F and Supplemental Figure 3.)*

Reference:

1 Kuppe, C., et al. Decoding myofibroblast origins in human kidney fibrosis. *Nature* 589, 281-286,
doi:10.1038/s41586-020-2941-1 (2021).

[3] Reviewer 1' Comment: The authors assert that i-Fibs dominate in areas with significant immune cell infiltration. To substantiate this claim, it is crucial to provide empirical data demonstrating the prevalence of i-Fibs in these regions. Detailed quantitative analyses, possibly including spatial mapping and correlation studies between i-Fibs and immune cell markers, should be presented to clarify this observation.

Authors' Response: We have now performed a series of new experiments and analyses to directly address this, and the new data have significantly strengthened our findings.

1. Direct quantification of i-Fib subpopulations in CKD: First, we performed multiplex RNAscope staining and statistical analysis to directly quantify the abundance of our identified i-Fib subpopulations. Our results confirm a significant increase in the numbers of both *CCL19+DCN+* (Cluster 2) and *CCL2+DCN+* (Cluster 3) i-Fibs in patient CKD tissues compared to their sparse distribution in normal kidney tissue. Crucially, the abundance of these i-Fib populations correlates positively with the degree of tissue fibrosis and negatively with renal function, underscoring their pathological relevance (newly added Figure 1, D to I and Figure S3).

Figure 1, D to I: The distribution of ECM-Fibs and i-Fibs in CKD tissues.

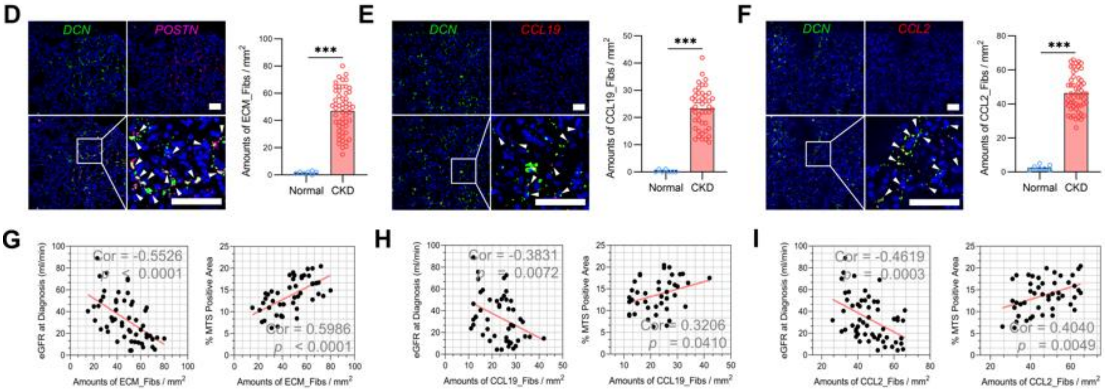

Figure S3: The distribution of ECM-Fibs and i-Fibs in human normal kidney.

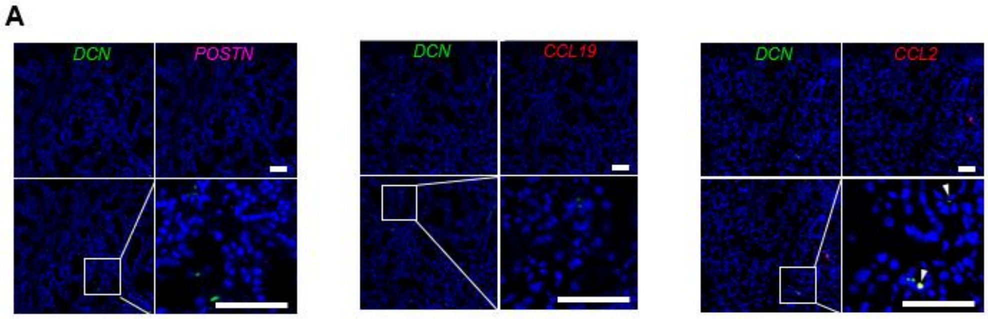

2. Demonstrating the spatial link between i-Fibs and T cell infiltration: To directly investigate the relationship between i-Fibs and immune cells, we focused on the less-characterized CCL19＆21hi Fibs.

Using immunofluorescence (IF), we first confirmed that co-receptor of CCL19/21, CCR7, is primarily expressed on T cells, and that these CCR7+ T-cells are significantly upregulated in CKD tissue (new Figure S4A). Strikingly, our new combined RNAscope and IF analysis revealed that local inflammatory niches (marked by T cell infiltration) and fibrogenic niches (marked by COL1A1 deposition) are spatially segregated and mutually exclusive (new Figure S4B). (*See the line 156-191. The total Results part "Fibroblasts establish functionally distinct and mutually exclusive pathological niches in injured kidneys"*)

Most importantly, quantitative spatial analysis provided direct evidence for our claim: the local density of i-Fibs (specifically CCL19+ Fibs) shows a strong positive correlation with T-cell infiltration but a negative correlation with COL1A1 deposition. Conversely, ECM-Fibs exhibit the exact opposite relationship. These data provide direct, empirical evidence of a tight spatial association between i-Fibs and infiltrating T-cells (newly added Figure 1, K to N).

Figure S4, A and B

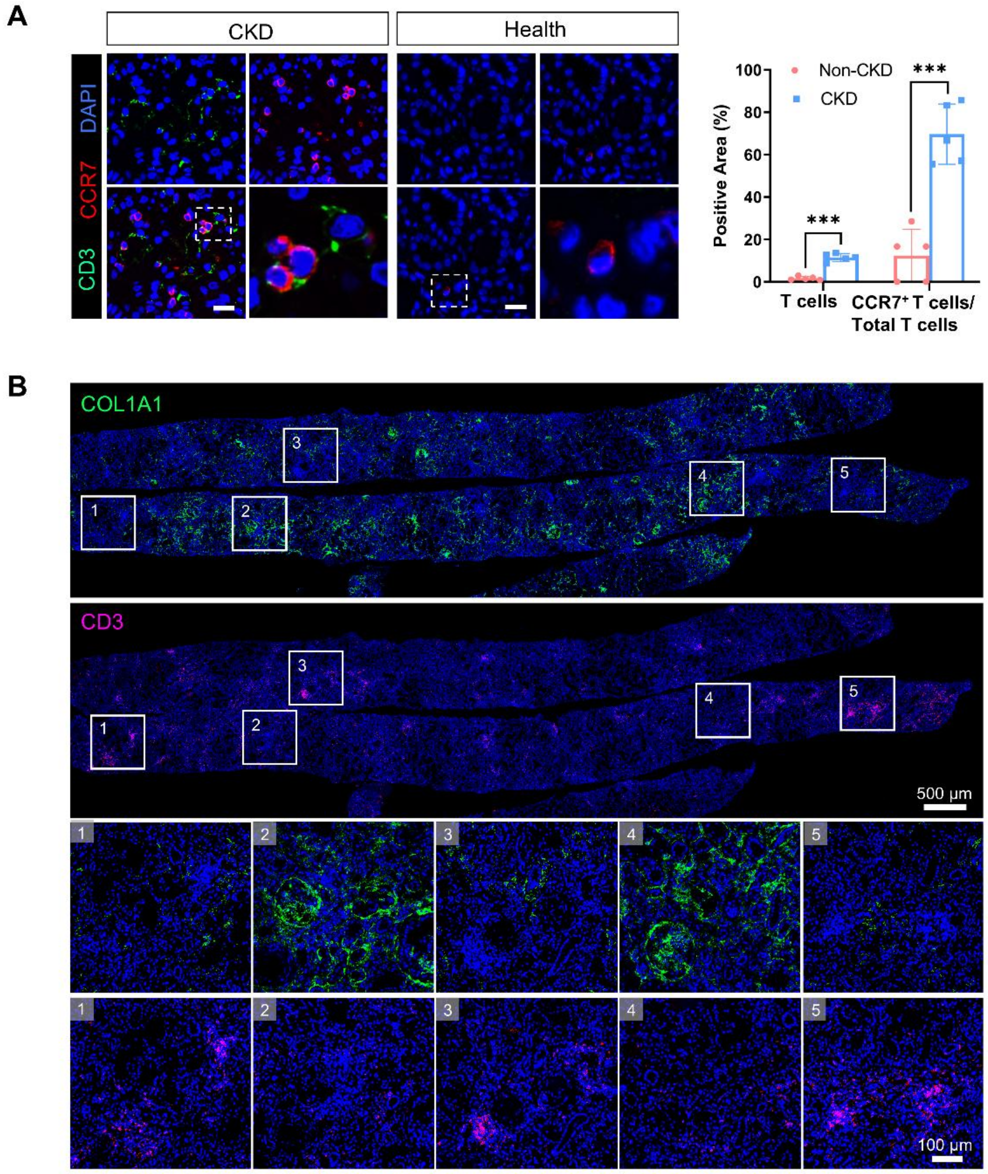

Figure 1, K to N

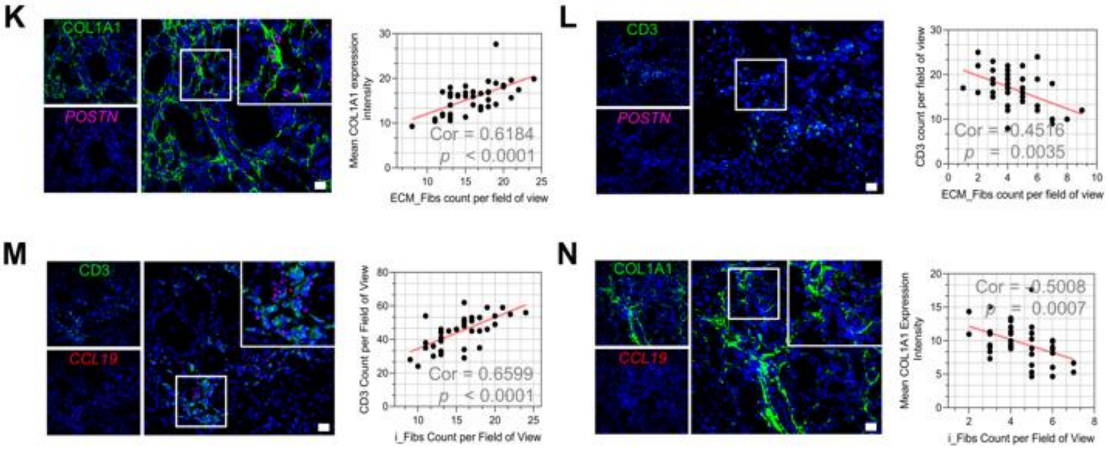

3. Contextualizing the significance of our findings: Finally, these new findings are particularly timely. A recent landmark study in *Nature Genetics* used high-resolution spatial transcriptomics to identify an "immune microenvironment" in CKD (*1*), characterized by T/B cell markers and, notably, high expression of CCL19, CCL21, and CXCL12—the signature genes of our CCL19&21hi Fibs. Our study builds directly upon this by providing, for the first time, the precise single cell identity of the key cellular architects of this niche. We not only name these cells but also demonstrate their direct, positive spatial relationship with infiltrating T cells. The significance of these findings is now addressed in the revised Discussion. (*See the line 498-512. The second paragraph of the Discussion part: A recent high-resolution spatial atlas of CKD has revealed distinct microenvironment subtypes of renal stromal areas, often presenting as "mosaic" distribution dominated by fibrogenic microenvironment (FME) and immune microenvironment (IME). The former is characterized by matrix activation markers like POSTN, while the latter is enriched with lymphocytes, as well as chemokines such as CCL19, CCL21, CXCL12 and their receptors. In this study, we provided a critical cellular dimension to the establishment of this spatial map. Through integrated ScRNA-seq analysis and multiplex RNAscope, we identified a previously uncharacterized fibroblast subset, termed CCL19&21hi-Fibs, whose localization is strongly correlated with that of CCR7+ lymphocytes, a defining feature of the IME. Besides, our data revealed that ECM-Fibs act as the architects of the FME by remodeling stromal biomechanical properties. Interestingly, we found that the distinct fibroblast populations were spatially segregated, occupying mutually exclusive territories that correspond to either the IME or FME.*)

We are very grateful for your guidance, as these new experiments have substantially reinforced our central hypothesis and added significant depth and novelty to our manuscript.

Reference:

1 Abedini A., et al. Single-cell multi-omic and spatial profiling of human kidneys implicates the fibrotic microenvironment in kidney disease progression. Nat Genet 56(8):1712-1724. doi: 10.1038/s41588-024-01802-x (2024).

[4] Reviewer 1' Comment: The manuscript is divided into two sections: the first part demonstrates fibroblast heterogeneity by showing that CD248 is specifically expressed in a subset of fibroblasts designated as ECMhi-Fibs. The second part investigates the role of CD248 in renal fibrosis induced by unilateral ureteral obstruction (UUO) and unilateral ischemia-reperfusion injury (uIRI), using various genetic mouse models, including CD248 knockout (KO) mice and conditional CD248 KO mice (Col1a2-CreERT mice). It is imperative that the authors provide a rationale for not employing a knockout strategy specific to ECMhi-Fibs. This explanation is crucial, especially given the specific expression of CD248 in this subpopulation, as a targeted knockout could potentially yield more direct insights into the functional role of CD248 within these particular cells.

Authors' Response: We thank you for this important question regarding our choice of genetic strategy. This was a deliberate decision based on two primary considerations: ensuring lineage specificity and aligning with our therapeutic objective.

1. Rationale for ensuring fibroblast lineage specificity: While using a marker for ECMhi-Fibs, such as *Postn*, is an intriguing idea, markers for activated fibroblasts can sometimes be expressed by other non-fibroblast stromal cells during injury, such as endothelial cells (*1*). This could lead to off-target recombination and confound the interpretation of the results. In contrast, Col1a2 is a well-established and highly specific lineage-defining marker for fibroblasts. By using the *Col1a2-*

CreERT model, we could confidently restrict CD248 deletion specifically to the fibroblast population, which was crucial for our study's foundational claims.

2. The second part of our manuscript was designed to test a broader therapeutic hypothesis: whether targeting CD248 across the entire fibroblast compartment can mitigate renal fibrosis. This approach closely mimics the action of a systemic therapeutic agent, like our IgG78 antibody, which would engage CD248 on all expressing fibroblasts, not just a single predefined subset. Therefore, evaluating the effect of a pan-fibroblast CD248 knockout is more directly relevant to assessing the therapeutic potential of our antibody.

We have revised our manuscript to more precisely frame the scope of our knockout strategy. We thank the reviewer for their insightful suggestion to use subset-specific Cre lines in the future, which we agree is a valuable next step for the field.

(*See the line 311-317. The second paragraph of the Results part "Specific depletion of CD248 in fibroblasts inhibits myofibroblast activation and reduces fibrotic progression": <u>While our studies using both the uIRI and UUO models further strengthened the evidence for the anti-fibrotic effects of global Cd248 deletion, the key outstanding question was whether this anti-fibrotic effect is primarily driven by its deletion in fibroblasts. To address this directly, we employed the well-established pan-fibroblast Col1a2-Cre driver to specifically delete Cd248 within the fibroblast lineage. This allowed us to precisely assess the cell-autonomous contribution of fibroblast-expressed CD248 to their phenotypic state and pro-fibrotic function in vivo.</u>*)

Reference:

1 Barnett SN., et al. An organotypic atlas of human vascular cells. Nat Med 30(12):3468-3481. doi: 10.1038/s41591-024-03376-x (2024).

[5] Reviewer 1' Comment: The study describes the engineering of two truncated forms of CD248: CD248 ΔECD, which lacks the extracellular domain (ECD), and CD248 ΔCTLD, which lacks the C-type lectin domain (CTLD). It is essential to include detailed methodology for the engineering and validation of these truncated proteins in the Methods section of the manuscript. Specifically, the

procedures for constructing these variants, the expression systems used, and the validation assays to confirm their structural integrity and functional activity should be shown.

Authors' Response: We thank you for this suggestion to enhance the methodological detail regarding our CD248 truncated constructs. As requested, we have substantially expanded both the Methods and Results sections to provide a comprehensive account of the construction, expression, and validation of these critical reagents.

Specifically, we have now added the following details:

1. Detailed construction and expression methodology: The Methods section now explicitly describes the construction of four key lentiviral expression vectors: ①A full-length CD248 control. ② The extracellular domain deletion mutant ($CD248^{\Delta ECD}$, retaining the transmembrane and cytoplasmic domains, amino acids 696-765). ③The C-type lectin-like domain deletion mutant ($CD248^{\Delta CTLD}$, retaining amino acids 157-766).

We clarify that these sequences were cloned into the Ubi-MCS-3FLAG-SV40 lentiviral backbone. The protocol now also details how these vectors were co-transfected with packaging plasmids (psPAX2 and pMD2G) into HEK-293T cells to produce viral supernatants, which were subsequently used to transduce CD248-null fibroblasts for our rescue experiments.

(*See the Supplementary Materials and Methods part "Specific depletion of CD248 in fibroblasts inhibits myofibroblast activation and reduces fibrotic progression"<u>: For overexpression studies, several constructs were cloned into the Ubi-MCS-SV40 lentiviral vector. These included full-length murine CD248 (amino acids 1-766) with or without an N-terminal EGFP tag, a CD248 mutant lacking the extracellular domain (amino acids 715-766), and a CD248 mutant with a deletion of the C-type lectin-like domain((amino acids 157-766).</u>*

<u>Lentiviral particles were produced in HEK293T cells by co-transfecting the respective lentiviral plasmid with the packaging plasmids psPAX2 and pCMV-VSV-G using Lipofectamine 2000 (Invitrogen). The medium was replaced 12 hours post-transfection, and viral supernatants were harvested at 48 and 72 hours, pooled, and centrifuged at 3,000 g for 10 minutes at 4°C to remove</u>

cell debris before filtration through a 0.45 μm sterile filter. For transduction, primary fibroblasts were seeded and exposed to viral supernatant diluted 1:1 with fresh culture medium, supplemented with 6 μg/mL polybrene (Sigma-Aldrich). After 24 hours, the virus-containing medium was replaced with fresh medium containing 2 μg/mL puromycin (Sigma-Aldrich) for selection. Selection was carried out for 5-7 days, and the efficiency of overexpression, knockout, or knockdown was validated by Western Blot analysis.)

2. Rigorous structural and functional validation: We have strengthened the manuscript by including clear validation data for these constructs: ①Structural validation: We have now included Western Blot data**,** presented in the new Figure 7D, which confirms the stable expression of the truncated proteins at their expected molecular weights in the transduced fibroblasts. ②Functional validation: To confirm the functional consequences of these deletions, we performed a series of rescue experiments. We demonstrated that while re-expression of full-length CD248 successfully rescued the ECM-producing phenotype in CD248- fibroblasts, the $CD248^{\Delta CTLD}$ mutant failed to do so (Figure 7E). Critically, we further showed that fibroblasts expressing the $CD248^{\Delta ECD}$ and $CD248^{\Delta CTLD}$ mutant did not exhibit activation of the mechanotransduction pathway, in stark contrast to wile type CD248+ fibroblasts (Figure 7F).

Figure 7D (structural validation)

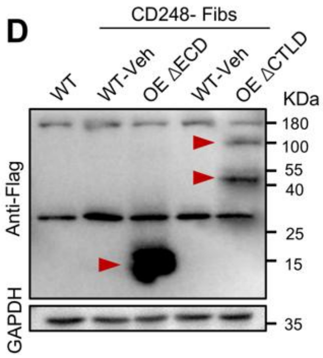

Figure 7, E and F (functional validation)

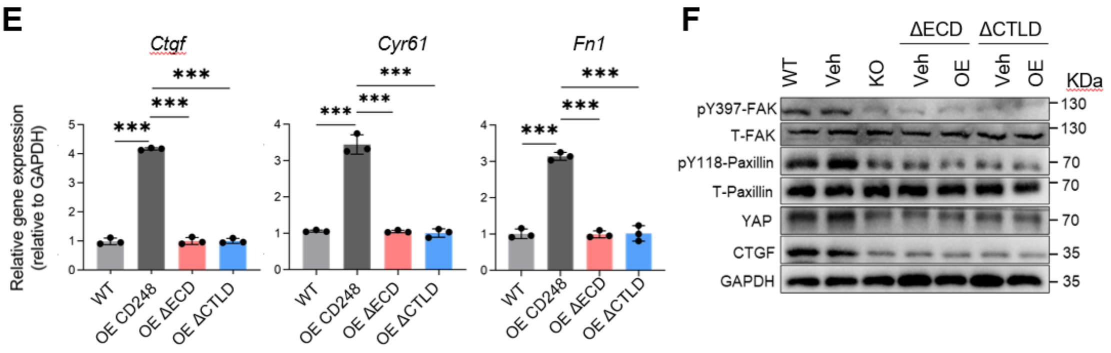

Together, these detailed additions to the Methods and Results provide further validation for our constructs and empirically demonstrate that the C-type lectin-like domain (CTLD) is indispensable for the pro-fibrotic, mechanosensory function of CD248. We hope these comprehensive additions will address your request and significantly strengthen the methodological rigor of our study.

(*See the line 403-413. The first paragraph of the Results part "CD248 mediates mechanotransduction by recruiting IQGAP1-ARF6·GTP via interaction with aberrant ECM": To dissect how CD248 bridges aberrant ECM to fibroblast activation, we generated two deletion mutants: one lacking the extracellular domain (CD248-ΔECD) and another specifically missing the C-type lectin-like domain (CD248-ΔCTLD), which is critical for its interaction with the ECM. (**Figure 7C**). Following confirmation of successful construct expression by Western blot (**Figure 7D**), RT-qPCR was conducted to find that the mRNA levels of Ctgf, Cyr61, and Fn1 were specifically elevated in the CD248 overexpressing group, but not in the other groups (**Figure 7E**). Moreover, overexpression of exogenous CD248 ΔECD and CD248 ΔCTLD fails to restore the response of fibroblasts to ECM, including mechanical activation, focal adhesion formation and YAP nuclear translocation (**Figure 7, F to I**). These data demonstrated that CD248-mediated interaction between ECM and myofibroblasts is essential for maintaining the mechanical feedback loop during myofibroblast activation.*)

Minor questions:

[6] Reviewer 1' Comment: Line 97: Monocle antibodies (mAbs) should be monoclonal antibodies.

Authors' Response: We thank the reviewer for their careful reading and for pointing out this typographical error. We have corrected "monocle" to "monoclonal" throughout the revised manuscript as suggested.

[7] Reviewer 1' Comment: Line 150: Please specify what is meant by the term corresponding controls in that context.

Authors' Response: We apologize for the ambiguity in our original description. The term "corresponding controls" referred to publicly available mRNA-seq datasets from healthy, non-diseased kidney tissue. However, we agree with you that this term was imprecise.

More importantly, prompted by your comment, we re-evaluated the methodological rigor of this analysis. The original approach relied on integrating bulk RNA-seq datasets from different sources using the "ComBat" algorithm. While this bioinformatic strategy has been used in published studies, we concluded that it was not sufficiently robust to definitively support our claims.

Therefore, to improve the quality and rigor of our study, we have completely removed this analysis and the original Supplemental Figure 3A from the revised manuscript. To replace this, we have performed a new experiment. We used multiplex RNAscope *in situ* hybridization to simultaneously visualize and quantify both ECM-Fibs and i-Fibs directly on clinical tissue sections. This analysis provides direct visual and quantitative evidence that the abundance of both fibroblast subsets is significantly increased in kidney tissue from CKD patients compared to normal adjacent tissue.

As detailed in our response to your comment [3], these new and more definitive data are now presented in the revised Figure 1, D to I and Figure S2.

We are very grateful to you for this insightful comment, as it led us to replace a weaker, indirect

analysis with much stronger, more direct evidence, thereby significantly strengthening our manuscript's conclusions.

[8] Reviewer 1' Comment: Line 151: The heatmap does not show me an increase in ECM fibs or i-fibs. Please explain what is shown on the heatmap.

Authors' Response: We sincerely apologize for the lack of clarity in our original description of the heatmap. This figure did not adequately convey the intended message. In light of this, and as detailed in our response to your previous comment [7], we have removed this heatmap and replaced it with a more direct and quantitative spatial staining analysis.

[9] Reviewer 1' Comment: Line 176: The term "renal hypofunction" should be replaced with "renal dysfunction" or "renal insufficiency".

Authors' Response: We agree that "renal dysfunction" is the more appropriate term. We have corrected this throughout the manuscript, *as exemplified in the revised sentence on Line 164-166: Furthermore, correlation analysis revealed that the abundance of both ECM-Fibs and i-Fibs was positively correlated with both collagen deposition and <u>the severity of renal dysfunction</u>, as measured by the estimated glomerular filtration rate (eGFR).*

[10] Reviewer 1' Comment: Line 184-186: Which gene clusters are the authors referring to? Are they the same clusters described in line 140 or are they specific clusters in ECMhi-Fibs?

Authors' Response: We sincerely thank you for pointing out this lack of clarity in our description. You are correct that our original text was confusing, and we apologize for this oversight. The gene clusters mentioned here are not the same as the fibroblast gene signature but are instead groups of genes identified from the trajectory analysis that show dynamic expression changes as fibroblasts transition from an i-Fib to an ECM-Fib state.

To eliminate this ambiguity and improve the clarity of our manuscript, we have substantially revised

this section. The updated text now first describes the pseudotime trajectory itself and then explicitly introduces the two functionally distinct gene clusters that were derived from this analysis based on their dynamic expression patterns.

We believe this revised description, now located on *Lines 200-202*, makes it unequivocally clear that both of the gene clusters represent dynamic transcriptional programs, not any subpopulations, thereby fully addressing your concern: *To investigate the plasticity between fibroblast subsets, we performed trajectory inference analysis. This analysis suggested a potential differentiation path from i-Fibs towards ECM-Fibs, characterized by a progressive shift in gene expression: a gradual decrease in inflammatory chemokines was paralleled by an increase in extracellular matrix components along the pseudotime trajectory (Supplemental Figure 5, A to C). To understand the biological processes driving this transition, we examined the dynamic gene expression patterns along this trajectory. We identified two major, functionally distinct gene clusters based on their expression dynamics (Figure 1O).*

[11] Reviewer 1' Comment: Line 197: The authors should provide information why they only focus on ECMhi-Fibs (cluster 0).

Authors' Response: Thank you for this insightful question and for highlighting an area where our scientific rationale was not explicitly stated.

Our rationale is that the trajectory analysis positions ECM-Fibs as the terminal, pro-fibrotic effector cells that are the primary source of pathological matrix deposition in renal fibrosis. Therefore, targeting this specific subpopulation represents the most direct and impactful strategy to halt fibrosis progression. To clarify and explicitly present this idea in the manuscript, we have now inserted a transitional paragraph to bridge the gap. *The newly added text (Lines 224-225) is as follows: As the terminal pro-fibrotic effector population identified by our trajectory analysis, ECMhi Fibs represent a prime target for anti-fibrotic therapy. To identify targetable cell-surface markers on these cells, we intersected their differentially expressed membrane molecules (DEMMs) (logFC > 0.5, FDR < 0.25) with a comprehensive human membrane proteome database (Figure 2A and Table S5).*

[12] Reviewer 1' Comment: Line 209: The list of genes that are differently expressed between CD248+ and CD248- pericytes should be provided as supplementary table.

Authors' Response: Many thanks for your suggestion. We agree that providing this gene list is important for the transparency and completeness of our data. As requested, we have compiled the list of DEGs between CD248+ and CD248- fibroblasts and have included it as a new supplementary table in the revised manuscript (now Table S7). *See Lines 235-238: Herein, we noticed that CD248, which is significantly upregulated in activated fibroblasts of diverse organ fibrosis diseases, was specifically expressed in ECMhi Fibs. The volcano plot visualizing the differential expression analysis between CD248+ and CD248− fibroblasts indicated that compared with other fibroblasts, CD248+ fibroblasts exhibited the most significant differences in the expression genes encoding ECM molecules, notably associated with stromal activation and fibrosis progression, such as POSTN, COL1A1 and FN1 (Figure 2D, Table S7).*

[13] Reviewer 1' Comment: Line 213-216: The authors should refer to the respective figure showing multiplex immunohistochemical (mIHC) staining.

Authors' Response: We thank you for this helpful suggestion to improve clarity. We agree that the reference to the figure should be introduced earlier to ensure it clearly pertains to the entire description. We have revised the text accordingly and believe the new version is much clearer. *See Lines 242-243: Additionally, the results of multiplex immunohistochemical (mIHC) staining demonstrated that in renal tissues of CKD patients (Figure. 2E), CD248 was selectively co-localized with COL1A1 in the non-epithelial stroma.*

[14] Reviewer 1' Comment: Line 217: Information about the in-house CKD cohort should be provided and explained in the materials and methods section.

Authors' Response: We thank you for this constructive suggestion. A more detailed description of our in-house CKD cohort is important for transparency and rigor. To address this, we have

substantially expanded the "Patients and specimens" section within the Materials and Methods to include key details about the cohort's demographics, age range, and, crucially, the distribution of underlying disease etiologies:

Patients and specimens

This study was approved by the ethics committee of Xijing Hospital, Air Force Medical University (Xi'an, China), and all patient samples were collected with informed consent. The study cohort consisted of 56 patients with CKD who underwent percutaneous renal biopsy at our institution. This cohort included both male and female patients, with ages ranging from 24 to 79 years, and was representative of common CKD etiologies, including Diabetic Nephropathy, Lupus Nephritis, Hypertensive Nephropathy, and Chronic Glomerulonephritis. For control tissues, we utilized paraffin-embedded sections from 7 archived, pathologically normal nephrectomy samples from our hospital's tissue bank. Comprehensive clinical and demographic data, including disease etiology, eGFR, and serum creatinine levels, were collected from electronic medical records. A detailed summary of patient characteristics is provided in Table S4.

[15] Reviewer 1' Comment: Line 297: It should be specified which tissue was used for these analyses. Whole kidney lysates?

Authors' Response: We thank you for highlighting this critical omission. Specifying the tissue source for our transcriptomic analyses is essential. Bulk mRNA-seq experiments described in this section were performed on whole kidney tissue. We have now revised the manuscript to state this explicitly at the first mention of the analysis. *See Lines 338-339: To further investigate the molecular events in the kidney fibrogenic niche following CD248 deletion, we performed bulk mRNA-seq on whole kidney tissue of CD248-/- mice with fibrosis.*

[16] Reviewer 1' Comment: Line 301: CRISPR-Cas9-induced CD248 knockout in primary fibroblasts resulted in a decrease in pro-fibrotic ECM molecules as shown in Fig.5B. However, Fig.5B shows increased expression of Col1a2. The authors should comment on that result.

Authors' Response: We sincerely thank you for pointing out the apparent inconsistency regarding Col1a2 expression.

Our original, broad statement, "a decrease in pro-fibrotic ECM molecules", was imprecise and did not accurately reflect the nuance in the data. To address this and improve the clarity of our manuscript, we have revised the text. Instead of making a generalized claim, we now specifically highlight the consistent and significant downregulation of several canonical markers of fibroblast activation that strongly support our conclusion.

We believe this revised phrasing more accurately and rigorously presents our findings, focusing on the most critical and consistent changes, and thereby strengthening the overall conclusion of this experiment. We are grateful for the opportunity to make this important clarification. *See Lines 340-342: To further investigate the molecular events in the kidney fibrogenic niche following CD248 deletion, we performed bulk mRNA-seq on whole kidney tissue of CD248-/- mice with fibrosis. GSEA revealed that CD248 deficiency suppressed classical pro-fibrotic signaling pathways (Figure 5A). Mirroring our data from bulk renal tissues, CRISPR-mediated CD248 knockout in primary fibroblasts also downregulated canonical pro-fibrotic genes such as Col1a1, Postn and Fn1 (Figure 5B).*

[17] Reviewer 1' Comment: Line 305: The authors should refer to the respective figure.

Authors' Response: We thank the reviewer for pointing out this omission. We have revised the sentence to include the appropriate citation for Figure 5C, ensuring clarity and proper attribution of the data. *See Lines 346: The top overlap pathways were related to cell response to ECM and the activation of biomechanical signaling (Figure 5C). Intriguingly, the CD248-enriched pathways aligned with those driving fibroblast differentiation into ECMhi Fibs, suggesting a key role for CD248 in mechanotransduction-mediated ECMhi Fibs activation (we continue to use the term 'myofibroblasts' following, which is widely accepted in mouse models) (Figure 1P and 5C, and Table S8).*

[18] Reviewer 1' Comment: Line 350: There is a "," that needs to be deleted.

Authors' Response: We thank you for pointing out this typo. We have corrected it in the revised manuscript.

[19] Reviewer 1' Comment: Line 453-456: There are no data in the manuscript to show that CCL19&21 hi-fibs recruit immune cells. This should be stated as a hypothesis by the authors.

Authors' Response: We thank you for this highly constructive point.

To provide stronger support for this idea, we have substantially revised the Discussion. We now contextualize our findings with a recent landmark study on 'immune microenvironments' (IME) in CKD and highlight our multiplex RNAscope data, which demonstrates a direct spatial correlation between our novel CCL19&21hi-Fibs and CCR7+ lymphocytes.

Nonetheless, we agree that our data does not show direct recruitment. Therefore, as you rightly suggested, we have reframed this conclusion as a testable hypothesis. We believe this revision greatly improves the scientific accuracy of the manuscript and are grateful for the suggestion.

(*See the line 504-508. The Discussion part: In this study, we provided a critical cellular dimension to the establishment of this spatial map. Through ScRNA-seq analysis and multiplex RNAscope, we identified a previously uncharacterized fibroblast subset, termed CCL19&21hi-Fibs . The strong spatial correlation of these cells with CCR7+ lymphocytes leads us to hypothesize that they are the key architects of the IME, a defining feature of which is the presence of these immune cells.)*

[20] Reviewer 1' Comment: Line 494: The results highlight the role of the CTLD of CD248.

Authors' Response: We are extremely grateful to you for meticulous reading and for bringing this critical error to our attention. Our original text mistakenly referred to 'full-length CD248,' which was an oversight on our part and did not accurately reflect our data or our intended conclusion.

Our results, as you inferred, specifically pinpoint the CTLD as the indispensable domain. We have

now corrected the manuscript to state this clearly. *See Lines 413-414: This finding underscores the indispensable role of the CTLD in mediating the mechanosensory feedback loop that drives myofibroblast activation.*

[21] Reviewer 1' Comment: Line 1081: Figure 3H shows no statistical calculations.

Authors' Response: We thank you for this suggestion. We believe this comment refers to Figure 4H, and we agree that statistical analysis was needed. We have now performed the appropriate statistical tests on the quantification data from Figure 4G and have updated the revised Figure 4H accordingly.

[22] Reviewer 1' Comment: Line 1152: Please indicate which tissue was analyzed. Which tumour tissue was used here?

Authors' Response: We thank you for this important comment and agree that the tissue source was not clearly stated. We have now revised the manuscript to specify that all relevant analyses were performed on tissue from our renal fibrosis models.

[23] Reviewer 1' Comment: Line 1196-1200: Please rephrase, do not parenthesise the different groups.

Authors' Response: We thank you for this stylistic suggestion. We agree that the use of parentheses in this context was unconventional and may have hindered readability.

We have now revised the sentence to integrate the group descriptions directly into the text, removing the parentheses as requested.

[24] Reviewer 1' Comment: Figure 1B: The presented plots are not vulcano plots. In a vulcano plot the X-axis represents the logarithm of the fold change between conditions.

Authors' Response: We sincerely thank you for pointing out this significant inaccuracy. The plot was mislabeled and was not a true volcano plot.

We have corrected this in the revised manuscript. Specifically, we have removed the term 'volcano plot' from the legend and replaced it with the more accurate description, "Differential gene expression analysis". Furthermore, we have taken this opportunity to improve the legend's clarity by specifying the color coding for both significant (green) and non-significant (blue) genes.

The corrected legend as followed: ***(C)*** *Differential gene expression analysis showing up- and down-regulated genes across all fibroblast clusters. Genes with an adjusted P value < 0.01 are highlighted in green, while an adjusted P value ≥ 0.01 are indicated in blue.*

[25] Reviewer 1' Comment: Figure 1E: POSTN-expressing cells are not detectable.

Authors' Response: We sincerely thank you for your keen observation and for providing us with the opportunity to clarify the central message of this figure (now Figure 1J in the revised manuscript).

You are correct in noting that POSTN expression is nearly absent in the right-hand panels, under the "Immunoreactive foci" heading. This observation is, in fact, central to the scientific point we aim to convey with this figure: the stark spatial segregation and mutually exclusive nature of the two major fibroblast subpopulations, ECM-Fibs and i-Fibs.

To clarify, the figure is designed as a direct side-by-side comparison between two distinct pathological niches found within the same patient's tissue:

1. The "Fibrogenic foci" (middle panels): In these regions, which are characterized by dense collagen deposition (as confirmed by the strong blue Masson staining), there is a strong and clear signal for POSTN (magenta), the marker for ECM-Fibs. Importantly, in this same fibrotic area, the signal for the i-Fib marker, CCL19 (red), is minimal.

2. The "Immunoreactive foci" (right panels): Conversely, in separate regions characterized by prominent immune cell infiltration and sparse collagen, we observe a high expression of the i-Fib marker, CCL19 (red). It is precisely in this immune-rich niche that POSTN expression is, as the reviewer correctly observed, largely undetectable.

Therefore, the near absence of POSTN in the "Immunoreactive foci" is not a technical failure but rather a key biological finding. The figure demonstrates that POSTN-expressing ECM-Fibs and CCL19-expressing i-Fibs occupy distinct, non-overlapping spatial territories within the fibrotic kidney.

To prevent any future misinterpretation, we have substantially revised the figure legend for Figure 1J to more explicitly state that the figure's purpose is to highlight this mutual exclusivity and spatial partitioning between the two fibroblast subtypes: *(**J**) Analysis of consecutive kidney sections using RNAscope, PAS, and Masson staining. Fibrogenic foci, identified by heavy collagen deposition (Masson), are predominantly populated by POSTN+ ECM-Fibs (purple). Conversely, immunoreactive foci with dense immune infiltrates are occupied by CCL19+ i-Fibs (red) and are largely devoid of POSTN expression, demonstrating a clear spatial partitioning of the two fibroblast subtypes.*

Figure 6J

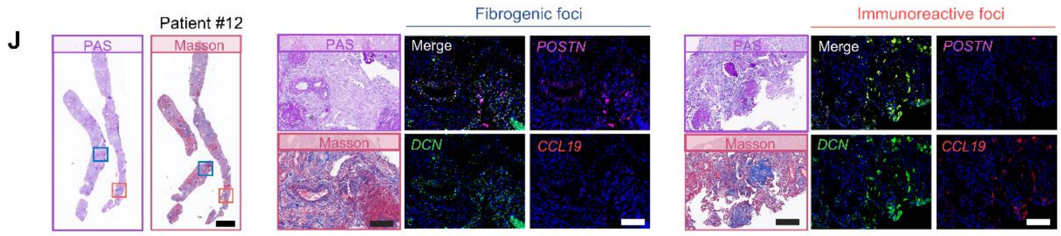

[26] Reviewer 1' Comment: Figure 1I: The KEGG enrichment is shown in Cluster2. It should be explained which cells Cluster2 refers to. In addition, the figure legend mentions Cluster 1, but Cluster 2 is shown in Fig1I.

Authors' Response: We sincerely thank you for identifying these important points of confusion.

1. Regarding the identity of the gene clusters: As we have detailed in our response to your comment [10], these "clusters" refer to functionally distinct gene sets identified from the pseudotime trajectory analysis, not fibroblast cell subclusters. To prevent this confusion, we have substantially revised the main text to explicitly define these gene sets before presenting the enrichment analysis.

2. Regarding the figure legend discrepancy: This was a typographical error. The legend should have referred to Gene Cluster 2, consistent with the figure's content. We have corrected this typo in the revised legend.

We are confident that these combined revisions have resolved the ambiguity surrounding this figure and its description.

[27] Reviewer 1' Comment: Figure 2B: The labelling of the figures must be explained in the legend (E-MTAB-2502...).

Authors' Response: We thank you for pointing out this omission. We agree that the data source for this analysis must be clearly stated. We have revised the legend for Figure 2B to explicitly include the accession numbers of the public datasets used.

The revised legend now reads: *(B) Correlation analysis of membrane protein gene expression with the ECM^{hi} Fibs signature score. <u>The analysis was performed on publicly available bulk RNA-seq datasets from human kidney biopsies (GSE66469 and E-MTAB-2502)</u>. Genes are ranked by their Pearson correlation coefficients.*

[28] Reviewer 1' Comment: Figure 2G-H: The labeling of the figures must to be explained. What is meant by "In-house data"?

Authors' Response: We agree that the term "In-house data" was not sufficiently specific, and that a more detailed description of our patient cohort is needed.

To address this, we have made the following revisions:

1. Expanded methods section: To provide greater detail, we have also added a comprehensive description of this cohort to the "Patients and specimens" section of the Methods. This text outlines the cohort's characteristics, ethics approval, and references the supplementary table (Table S4) for further information.

2. Revised figure legend: We have removed the term and revised the legend for Figure 2F and 2G to be more informative. The legend now reads: *(F and G) Correlation analysis of the COL1A1+CD248+ area with (F) the collagen deposition area and (G) eGFR* *__in our clinical CKD patient cohort (n = 56)__*. *Pearson correlation coefficients and P values are indicated.*

We hope these revisions provide the necessary clarity and detail regarding our clinical samples.

[29] Reviewer 1' Comment: Figure 3 H-O: The wild type control with uIRI is missing.

Authors' Response: We apologize if our figure legend or manuscript text was not sufficiently clear, which may have led to this misunderstanding.

We would like to clarify that the wild-type control with uIRI was indeed included in our study. To be precise, the experimental groups shown in Figure 3，L to Q are:

1. Sham Control: *Wile type* mice receiving saline instead of Diphtheria Toxin (DT).
2. uIRI Control (*Cd248*$^{wt/wt;TdTomato\text{-}DTR}$; Wild-Type Equivalent): DTR-negative littermates that underwent the uIRI procedure and received DT injections. This group serves as the proper wild-type control, as they are genetically wild-type for the DTR allele and thus their CD248+ cells are not depleted by DT.
3. uIRI + Depletion: *Cd248*$^{CreERT;\ TdTomato\text{-}DTR}$ positive mice that underwent the uIRI procedure and received DT to deplete $CD248^{+}$ cells.

Our results, which compare the uIRI Control (Group 2) with the uIRI + DTR (Group 3), directly demonstrate that the attenuation of fibrosis is specifically due to the depletion of CD248+ cells and not an artifact of the injury model or DT administration.

We have now revised the figure legend for [Figure 6] to state these experimental groups with greater clarity, ensuring there is no ambiguity: *Schematic illustrating the depletion of CD248^{+} cells in the uIRI model. Both experimental (Cd248$^{CreERT;\ TdTomato\text{-}DTR}$) mice and their Cre-negative littermate controls (Cd248$^{wt/wt;TdTomato\text{-}DTR}$) were subjected to uIRI followed by DT administration to ablate DTR-expressing cells.*

We hope this clarification resolves your concern and confirms the robustness of our experimental design.

[30] Reviewer 1' Comment: Figure 5C: The Venn diagram should include all numbers of KEGG pathways. Not only the overlap.

Authors' Response: We agree that including the number of pathways for each individual group is essential for proper interpretation. Accordingly, we have revised the Venn diagram in Figure 5C to include these numbers for each component, in addition to the overlap. We believe this revision makes the figure more informative and easier to understand.

Figure 5C

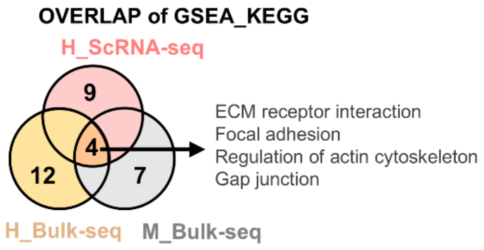

[31] Reviewer 1' Comment: Figure 5D: Why there are 2 bands visible for CD248?

Authors' Response: We thank you for this keen observation. The appearance of a doublet or multiple bands for CD248 on a Western Blot is an expected and well-characterized feature of this protein. This phenomenon is attributed to the extensive post-translational modifications, primarily N-linked glycosylation, which results in various glycoforms of the protein with distinct electrophoretic mobilities. This has been consistently reported in the literature for CD248/Endosialin (*1*).

Reference:

1 Kuppe, C., et al. aldez Y., et al. CD248: reviewing its role in health and disease. Curr Drug Targets 13(3):432-9. doi: 10.2174/138945012799424615 (2014).

[32] Reviewer 1' Comment: Figure 7B: Since both lines have a "+" in the same column, the panel indicates that CD248-pos and CD248-KO have been grown together. Please correct.

Authors' Response: We sincerely thank you for pointing out the ambiguity in the labeling of Figure 7B. You are correct that our original +/- system was confusing and could be misinterpreted.

To clarify the experimental design**,** all experiments were conducted on a single population of primary fibroblasts, which are inherently CD248-positive. The confusion arose from our labeling, where "CD248-pos: +" was intended to define the parental cell type, while "CD248-KO: +" indicated the specific experimental group in which CD248 had been successfully knocked out using CRISPR.

To completely resolve this ambiguity, we have revised the figure to include a more direct and intuitive labeling scheme. The experimental groups are now explicitly labeled as "Ctrl" (for fibroblasts transduced with a control/scramble vector) and "*Cd248*KO" (for fibroblasts with CRISPR-mediated knockout). This new labeling is now reflected directly on the Western Blot panel and is clearly defined in the updated figure legend.

Figure 7B

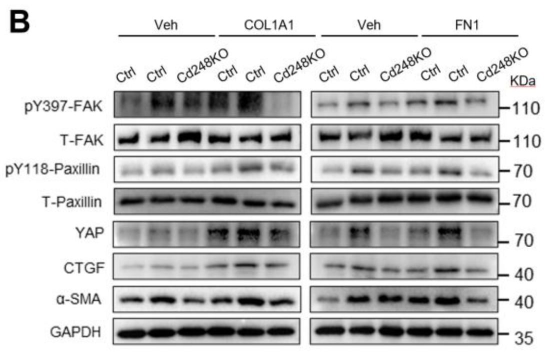

We are confident that this revised presentation eliminates any potential for misinterpretation and accurately reflects our experimental design. Thank you again for your valuable feedback, which has helped us improve the clarity of our manuscript.

[33] Reviewer 1' Comment: Figure 7H, I, J: Why there are 2 bands visible for CD248 in Figure 7H and 7I but not in Figure 7J?

Authors' Response: We sincerely thank you for your keen observation and for pointing out the inconsistency in the CD248 banding patterns across different panels in Figure 7.

The appearance of CD248 as a doublet (Figure7, H and I) or a single band (Figure7J) is due to its known post-translational modification, specifically glycosylation. As a heavily glycosylated protein, CD248 exhibits varying electrophoretic mobility, resulting in either multiple bands (representing different glycosylation states) or a single, broader band. This variability is well-documented in the literature and does not affect the protein's identification or the interpretation of its expression levels.

1. For instance, studies such as Yang et al. (*1*) have reported CD248 appearing as a doublet, consistent with our findings in panels H and I.

2. Conversely, other studies, such as Krishnan et al. (*2*), have shown CD248 as a single band.

To ensure consistency and clarity within our manuscript, we have now updated Figure 7L (previous Figure 7J) to display a representative blot that also shows the characteristic doublet pattern of CD248, matching panels H and I.

Figure 7L

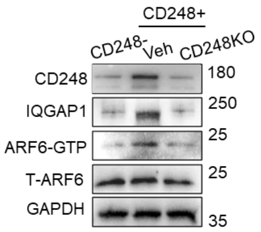

We are confident that this clarification, supported by the literature, and the revision to the figure fully address your concern. The major conclusion, that the CTLD domain is critical for CD248 function, remains robustly supported by the data.

Reference:

1 Yang F., et al. Interaction with CD68 and Regulation of GAS6 Expression by Endosialin in Fibroblasts Drives Recruitment and Polarization of Macrophages in Hepatocellular Carcinoma. Cancer Res 15;80(18):3892-3905. doi: 10.1158/0008-5472.CAN-19-2691. (2020).

2 Krishnan S., et al. CD248 induces a maladaptive unfolded protein response in diabetic kidney disease. Kidney Int 103(2):304-319. doi: 10.1016/j.kint.2022.09.024. (2022).

.

[34] Reviewer 1' Comment: Figure legends: The figure legends incorrectly use the term "qualification". It should be "quantification", e.g. line 1040, 1071, 1074, 1076, 1128 and many others.

Authors' Response: We are sincerely grateful for your meticulous attention to detail and for bringing this error to our attention.

We have now systematically corrected "qualification" to "quantification" in all instances throughout the entire manuscript, including all figure legends, the main text, and supplementary materials, to

ensure the accuracy and professionalism of our work. Thank you again for helping us improve the quality of our manuscript.

[35] Reviewer 1' Comment: Table S2: In which cluster CD248 is increased?

Authors' Response: Thank you for the opportunity to enable us clarify the specific purpose of this supplementary table.

First, please note that the table you are referring to (formerly Table S2) has been re-designated as Table S3 in the revised manuscript.

The primary purpose of this table is to showcase the top differentially expressed marker genes for each fibroblast subcluster, which were selected using highly stringent statistical criteria (avg_log2FC > 1 and p_val_adj < 0.001). While CD248 is undoubtedly a critical gene in our study and is expressed in the relevant clusters, its differential expression level did not meet this specific high threshold to be included in this particular list.

To provide further context, other key fibroblast activation membrane proteins, such as *PDGFRA* and *FAP*, are also absent from this table for the same reason. Therefore, the omission of CD248 from this list is a direct consequence of the strict filtering criteria applied to define only the most robust marker genes, and it does not impact our other conclusions regarding its functional importance.

[36] Reviewer 1' Comment: Figure S3A, B: The labeling of the figures needs to be explained in the legend (E-MTAB-2502…) The font size in the figures needs to be adjusted: for example, Figure S1 D and E. The small font size in the figures is difficult to read.

Authors' Response: Thank you for your valuable feedback on improving the clarity and labeling of our figures. We have addressed both points as follows:

1. Regarding the figure labeling: You correctly pointed out that the legend for the correlation analysis was missing the dataset identifiers. Please note that this figure is now designated as Figure S3C in the revised manuscript. We have updated its legend to be more informative, as you suggested: *Correlation analysis showing the relationship between ecm- , or i-Fibs and core ECM score of matrisome based on public mRNA-seq datasets (E-MTAB-2502 and GSE66494, respectively).*

Furthermore, to ensure full transparency, detailed information about these public datasets is now provided in this section: ***Data and materials availability:*** *All the expression data can be obtained from the public databases. ScRNA-seq data of CKD patients is available in the website (https://zenodo.org/record/4059315). For RNA-seq datasets of CKD patients, E-MTAB-2502 was download from Array Express database, and GSE66494 was download from gene expression omnibus (GEO) database. For normal kidney samples of human, RNA-seq data is available in GEO database (GSE173343).*

2. Regarding the font size: We have increased the font size in the specific figures you mentioned (Figure S1D and E) to ensure they are easily legible. Prompted by your feedback, we have also conducted a thorough review of all figures in the manuscript and adjusted fonts where necessary to improve overall readability.

Figure 1A (previous Figure S1D)

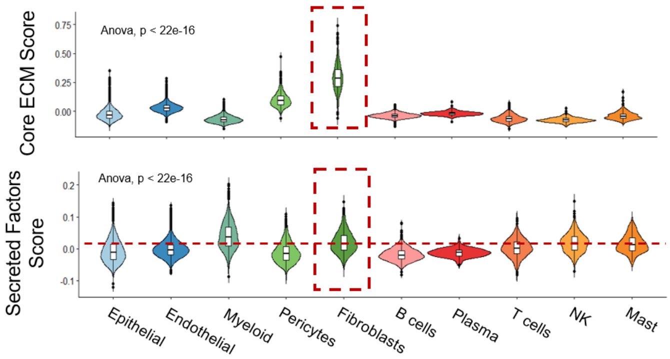

Figure S1D (previous Figure S1E)

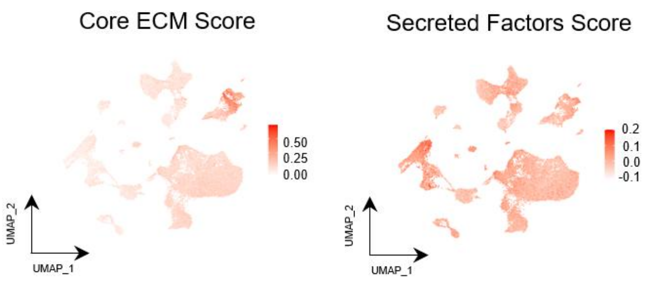

We are confident that these revisions have significantly improved the accessibility and professionalism of our figures.

Finally, we would like to extend our most sincere and heartfelt gratitude to you. Your comments, which ranged from major conceptual points that prompted new experiments to subtle but crucial details of presentation, pushed us to significantly strengthen our manuscript.

The paper is now substantially clearer and more scientifically sound as a direct result of the time and expertise you so generously dedicated to it. We are truly grateful for your invaluable contribution.

Reviewer #2

We sincerely thank you for the critical assessment of our manuscript. In the following response, we have addressed each of your concerns point-by-point. To do so, we have incorporated significant new experimental data, particularly regarding human tissue validation and therapeutic safety, and have carefully discussed the relevant literature to clarify our positions.

We believe these additional efforts have considerably strengthened the scientific rigor of our study, and we hope the revised manuscript now meets your high standards.

Major question:

[1] Reviewer 2' Comment: [The role of CD248 in stromal remodeling and fibrosis has been explored extensively in other organ systems, such as cancer-associated fibroblasts (CAFs) in tumors. The extrapolation of these findings to kidney fibrosis lacks sufficient novelty to justify the claims of a breakthrough yet the discovery of a novel pathomechanism linked to mechanobiology.**]**

Authors' Response: We thank you for highlighting the established role of CD248 in the tumor microenvironment. We agree that CD248 is a known marker in cancer-associated fibroblasts (CAFs). However, we respectfully submit that our study represents a conceptual advance rather than a simple extrapolation of these findings to the kidney.

Our conclusion is supported by the following lines of evidence:

- **Data-driven discovery:** Our identification of the CD248-mechanotransduction-YAP axis was not based on *a priori* assumptions or extrapolation from tumor studies. Instead, it was rigorously derived from our own **unbiased multi-omic analyses**, including scRNA-seq, transcriptomics, and mass spectrometry (IP-MS). It was these objective data that specifically guided us to uncover the interaction between CD248 and the mechanosensitive machinery (IQGAP1/ARF6), pointing to a function previously unrecognized in the context of CD248 biology.

• **Distinct functional mechanism:** As the reviewer correctly noted, previous studies in CAFs have extensively explored CD248. However, these studies have primarily focused on its regulation of cell adhesion and migration. To our knowledge, there is no clear report linking CD248 to intracellular mechanotransduction signaling, nor describing the phenotype where CD248 actively remodels the biomechanical properties of the ECM.

Therefore, the CD248-IQGAP1/ARF6-Focal Adhesion-YAP loop elucidated in our study represents a novel pathomechanism. It not only highlights CD248's role in cellular mechanosensing but also reveals how this signaling actively reshapes the stiffness of the surrounding matrix niche. This bidirectional feedback mechanism, to the best of our knowledge, is a fundamental advance that has not been reported in either tumor or fibrosis research.

[2] Reviewer 2' Comment: [The molecular pathway involving CD248, IQGAP1, and ARF6·GTP is described as novel; however, similar mechanotransduction pathways involving focal adhesions and YAP signaling are well-documented (PMID: 31040262). The study fails to clarify how the CD248 pathway is distinct from already-established mechanisms.**]**

Authors' Response: We thank you for pointing out this reference (Ghosh *et al.*, PMID: 31040262). Upon detailed comparison, we find that the mechanism identified in our study is fundamentally distinct from the cited work.

To summarize the key distinctions:

1. The cited work (Ghosh, et al.): Investigates CD13-mediated integrin recycling as a mechanism for cell migration. The described pathway facilitates the physical movement of cells.

2. Our study: Defines CD248 as a core component of a sustained mechanotransduction feedback loop that drives pathological niche formation and fibrosis via YAP activation. The described pathway promotes pathological matrix deposition, not cell movement.

In summary, while there are general thematic parallels regarding focal adhesions, the specific CD248-driven mechanotransduction loop described here is a novel discovery. It represents a

mechanism of fibrosis rather than migration.

[3] Reviewer 2' Comment: [The use of mouse models such as unilateral ureteral obstruction (UUO) and ischemia-reperfusion injury (uIRI) provides limited relevance to the human context. These models fail to replicate the complexity of chronic kidney disease (CKD) progression, especially the contributions of inflammation, comorbidities, and systemic factors seen in human patients. As such it remains unclear how similar the CD248 population in human vs. mouse are. The stainings shown in Figure 3a-d suggest that CD248 fibroblasts/myofibroblasts are localized in the entire interstitium of the mouse kidney, yet in the human tissue this is not the case in fibrosis. Single-cell RNA sequencing of those tdtom positive cells would be necessary and a comparison to tdtom-PDGFRbeta cells to explore the identify of these cells.**]**

Authors' Response: We appreciate the opportunity to address this multifaceted concern. For clarity, we have broken down this critique into three distinct components, which we will address sequentially:

① **The translational relevance of the UUO and uIRI mouse models:** Your raises an important point regarding the complexity of human CKD compared to preclinical models. While we agree that no single mouse model perfectly replicates the systemic comorbidities of human CKD, the UUO and uIRI models are widely recognized as robust tools specifically for studying renal fibrogenesis and myofibroblast activation—the core focus of our study.

These models have been successfully utilized in numerous high-impact studies to identify clinically relevant mechanisms and targets (e.g., *Kuppe et al., Nature 2021*; *Tanaka et al., Science Translational Medicine 2022*). More recently, *Reck et al. (Nature Communications, 2025)* and *Xiao et al. (Kidney International, 2025)* have employed these models to decode pro-fibrotic phenotypes similar to those investigated here. Therefore, we believe these models are appropriate and valid for dissecting the specific matrix-remodeling mechanisms driven by CD248. (*1-4*).

Reference:

1 Reck, M., *et al*. Multiomic analysis of human kidney disease identifies a tractable inflammatory and pro-fibrotic tubular cell phenotype. *Nat Communications* 16, 4745, doi: 10.1038/s41467-025-59997-4 (2025). [Both UUO and uIRI models for renal fibrosis during CKD progression]

2 Xiao A., *et al*. Gene signature-guided drug screening identified narciclasine as a potential therapeutic for interstitial fibrosis of the kidney. Kidney International 108(1):74-89, doi: 10.1016/j.kint.2025.03.021. (2025). [Both UUO and uIRI models for renal fibrosis during CKD progression]

3 Kuppe, C., *et al.* Decoding myofibroblast origins in human kidney fibrosis. *Nature* 589, 281-286, doi:10.1038/s41586-020-2941-1 (2021). [UUO for renal fibrosis during CKD progression]

4 Tanaka S., *et al*. Sphingosine 1-phosphate signaling in perivascular cells enhances inflammation and fibrosis in the kidney. Science Translational Medicine 17;14(658):eabj2681, doi: 10.1126/scitranslmed.abj2681. (2022). [uIRI for renal fibrosis during CKD progression]

② **The perceived discrepancy in CD248+ cell localization between mouse and human fibrotic tissue:** Your noted a potential discrepancy based on the comparison between our mouse tdTomato reporter images which reflect cumulative lineage history and human protein staining.

To address this and provide a direct, equivalent comparison, we have now performed new multiplex immunofluorescence for CD248 protein in both mouse and human fibrotic kidneys. These new data (Figure 2, D and E) demonstrate a highly consistent protein expression pattern between species. In both mouse uIRI and human CKD tissues, CD248 protein is specifically concentrated in the fibrotic interstitium. This aligns with established literature (*Smith et al., Kidney Int 2011; Xu et al., FASEB J 2022*) showing that CD248 expression significantly intensifies and expands with disease progression.

Figure 2, D and E (CD248 protein expression in mice and human renal fibrosis)

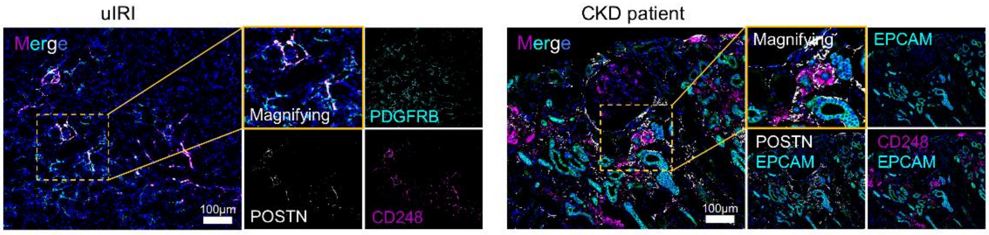

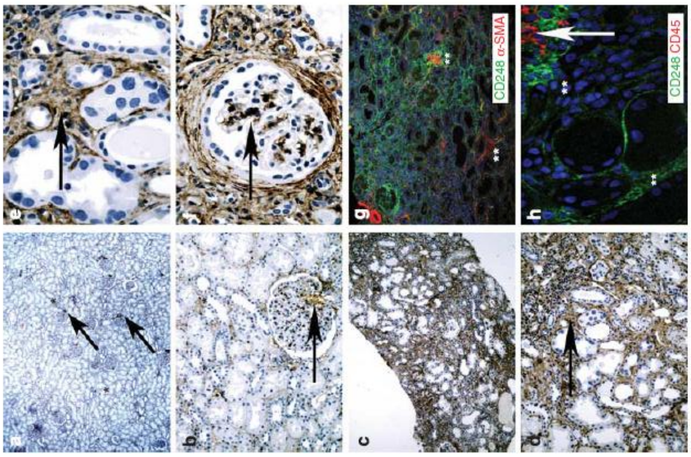

CD248 expression during CKD (*1*)

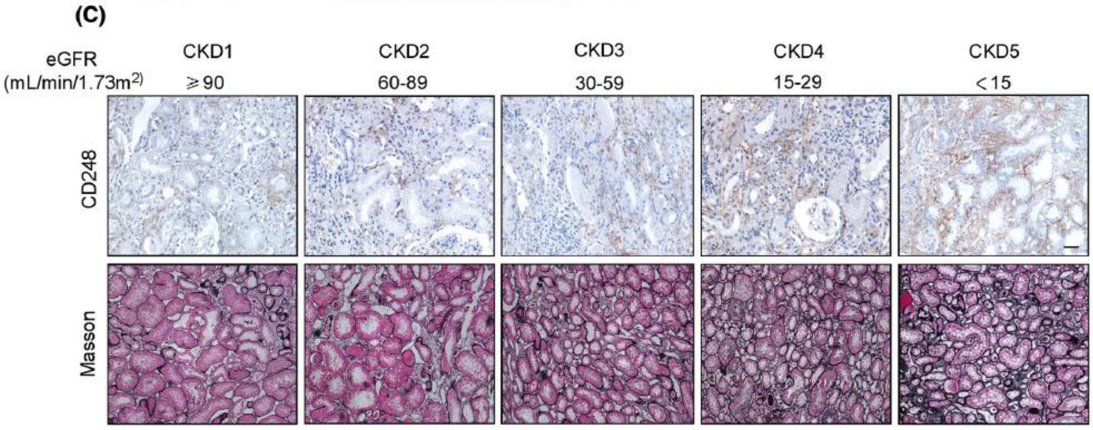

CD248 expression with CKD progression (*2*)

Reference:

1 Smith SW., *et al*. CD248+ stromal cells are associated with progressive chronic kidney disease. *Kidney Int* 80(2):199-207. doi: 10.1038/ki.2011.103 (2011).

2 Xu C., *et al*. Antibody-drug conjugates targeting CD248+ myofibroblasts effectively alleviate renal fibrosis in mice. *FASEB J* 36(2):e22102. doi: 10.1096/fj.202101441R (2022).

3 Wilhelm A., *et al*. CD248/endosialin critically regulates hepatic stellate cell proliferation during chronic liver injury via a PDGF-regulated mechanism. *Gut* 65(7):1175-85. doi: 10.1136/gutjnl-2014-308325 (2016).

4 Mogler C., *et al*. Hepatic stellate cell-expressed endosialin balances fibrogenesis and hepatocyte proliferation during liver damage. *EMBO Mol Med* 7(3):332-8. doi: 10.15252/emmm.201404246 (2015).

③ **The suggestion for additional scRNA-seq of sorted TdTomato+ cells:** We appreciate your suggestion to further characterize these cells. Regarding the proposed comparison with PDGFRβ+ cells, recent atlases (*Kuppe et al., Nature 2021*) indicate that PDGFRβ is a broad pan-mural marker expressed by pericytes and diverse fibroblast subsets, which may limit its utility as a specific control for identifying distinct pro-fibrotic populations.

To address the goal of functionally characterizing CD248+ fibroblasts with high resolution, we performed a targeted transcriptomic comparison. We FACS-sorted TdTomato+ (CD248-lineage) versus TdTomato- fibroblasts from fibrotic kidneys and performed bulk RNA-sequencing. The results (Figure 3, F to J) are conclusive:

- The CD248+ population is significantly enriched for genes driving matrix remodeling (e.g., *Postn, Ccn2, Fn1*).
- In contrast, classical inflammatory factors (*C3, Ccl2*) were downregulated.
- Critically, while activation markers were distinct, the expression of pan-fibroblast markers *Pdgfra* and *Pdgfrb* was indistinguishable between groups, further highlighting that CD248 marks a specific functional state rather than a broad lineage.

These data demonstrate that CD248 defines a functionally distinct, pro-fibrotic subset, directly addressing the need to dissect fibroblast heterogeneity.

Figure 3, F to J

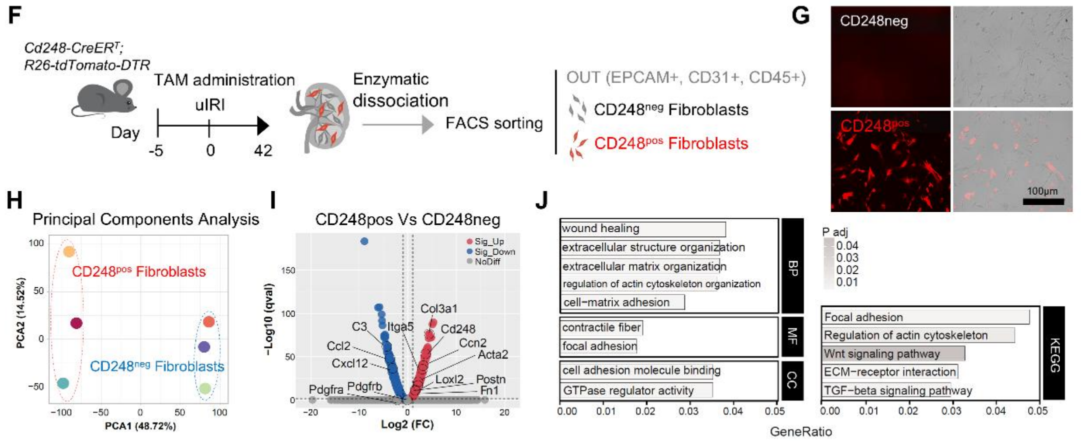

(*See the line 272-282. The second paragraph of the Results part "Selective tracing and ablation of CD248+ fibroblasts in a genetic mouse model reveal their role in facilitating stromal remodeling": To further identify the function of CD248+ fibroblasts, fluorescence-activated cell sorting (FACS) was used to isolate CD248+ (TdTomato+) primary fibroblasts (CD45–CD31–EPCAM–) from the kidneys of uIRI mice (Figure 3, F and G and Supplemental Figure 6B). Subsequent bulk mRNA-seq revealed that CD248+ fibroblasts were transcriptionally distinct from CD248- cells (Figure 3H). Specifically, we found ECM-related genes such as Postn, ccn2, Acta2 and Fn1 were upregulated in CD248+ fibroblasts, by contrast, inflammatory factors such as C3, Ccl2 and Cxcl12 were downregulated, similar with our findings in ScRNA-seq data shown in ECM^{hi} Fibs (Figure 3I). qRT-PCR validation confirmed that markers of stromal activation were significantly enriched in the CD248+ myofibroblast population (Supplemental Figure 6C). Functional analysis indicated that the transcriptional signature of CD248+ myofibroblasts was dominated by pro-fibrotic pathways (Figure 3J). These findings demonstrated that CD248+ fibroblasts represented a subset contributing to excessive ECM deposition in the renal fibrogenic niche.*)

This key finding demonstrates that CD248 defines a functionally distinct, pro-fibrotic state that cannot merely be defined by PDGFR expression levels. This directly addresses the critical need for a more specific membrane marker to dissect fibroblast heterogeneity, the central premise of our study.

Reference:

1 Kuppe, C.*, et al.* Decoding myofibroblast origins in human kidney fibrosis. *Nature* 589, 281-286, doi:10.1038/s41586-020-2941-1 (2021).

[4] Reviewer 2' Comment: [The authors do not adequately address the differences in CD248 expression and fibroblast phenotypes between human CKD and the mouse models. Given these discrepancies, the translational potential of the findings is questionable.**]**

Authors' Response: We appreciate your emphasis on ensuring translational relevance. This comment aligns with the concerns raised in Comment [3] regarding the comparison between human and mouse CD248 expression patterns.

As detailed in our response to Comment [3], we have addressed this by performing a direct, side-by-side comparison of CD248 protein expression in both species to eliminate any potential ambiguity arising from the previous reporter-based data.

Key clarifications and new data:

1. Consistent protein localization: Our new immunofluorescence data (Figure 3, above comment) confirm that CD248 protein expression is highly conserved between human CKD and the mouse uIRI model. In both species, CD248 is specifically upregulated in activated fibroblasts within the fibrotic interstitium.

2. Resolution of discrepancy: The perceived discrepancy likely arose from comparing the broad mRNA lineage tracing (TdTomato) in our initial mouse data with protein staining in human tissue. By comparing protein-to-protein in the revised manuscript, we demonstrate that the spatial distribution is indeed consistent.

3. Support from Literature: This conserved expression pattern is further supported by previous studies *(e.g., Smith et al., Kidney Int 2011; Xu et al., FASEB J 2022)*, reinforcing the validity of our mouse models for studying human renal fibrosis *(1-4)*.

Therefore, we believe the new data clearly demonstrate that the CD248-driven mechanism is conserved across species, supporting the translational potential of our findings.

Reference:

1 Smith SW., *et al*. CD248+ stromal cells are associated with progressive chronic kidney disease. *Kidney Int* 80(2):199-207. doi: 10.1038/ki.2011.103 (2011).

2 Xu C., *et al*. Antibody-drug conjugates targeting CD248+ myofibroblasts effectively alleviate renal fibrosis in mice. *FASEB J* 36(2):e22102. doi: 10.1096/fj.202101441R (2022).

3 Wilhelm A., *et al*. CD248/endosialin critically regulates hepatic stellate cell proliferation during chronic liver injury via a PDGF-regulated mechanism. *Gut* 65(7):1175-85. doi: 10.1136/gutjnl-2014-308325 (2016).

4 Mogler C., *et al*. Hepatic stellate cell-expressed endosialin balances fibrogenesis and hepatocyte proliferation during liver damage. *EMBO Mol Med* 7(3):332-8. doi: 10.15252/emmm.201404246 (2015).

[5] Reviewer 2' Comment: [Although the single-cell RNA sequencing (scRNA-seq) data is central to identifying CD248+ fibroblasts, the analysis is not sufficiently rigorous: The clustering of fibroblast subpopulations lacks validation using independent datasets. Particularly it is well known that RNA and protein expression do not necessarily correlate. As the CD248 protein expression is central to the paper, I would suggest to repeat single cell RNA sequencing of human fibroblasts from kidney and adding CD248 oligolabeled antibodies for CITE-seq to gain deeper insights which fibroblasts express CD248 on their surface. Also for the other mural cells this would be important including pericyte populations.]

Authors' Response: We appreciate the reviewer's rigorous suggestion to use CITE-seq for simultaneously validating surface protein expression and transcriptional states. We fully agree with the underlying principle: establishing a direct link between CD248 mRNA, surface protein, and cell function is critical for the validity of our conclusions.

While we acknowledge the power of CITE-seq, we respectfully submit that we have addressed this specific goal through a multi-layered, orthogonal approach that provides robust functional

validation.

1. Direct linkage of CD248 lineage, protein, and transcriptome: Our experimental design (Figure 3, F–J and Figure 5O) was specifically tailored to bridge the gap between transcript and protein:

- We utilized the *Cd248*$^{CreERT2;R26\text{-}tdTomato}$ mouse model to genetically label the CD248 lineage.
- We then FACS-sorted these tdTomato-positive (CD248-lineage) fibroblasts from fibrotic kidneys.
- Crucially, our Western Blot analysis (Figure 5O) on these sorted populations provides direct proof: the tdTomato-positive cells show robust CD248 protein expression, while the tdTomato-negative fraction shows significantly lower expression.
- Concurrently, the bulk RNA-seq analysis of these tdTomato-positive cells (Figure 3, I and J) confirmed their pro-fibrotic transcriptional signature.

Figure 3, F to J

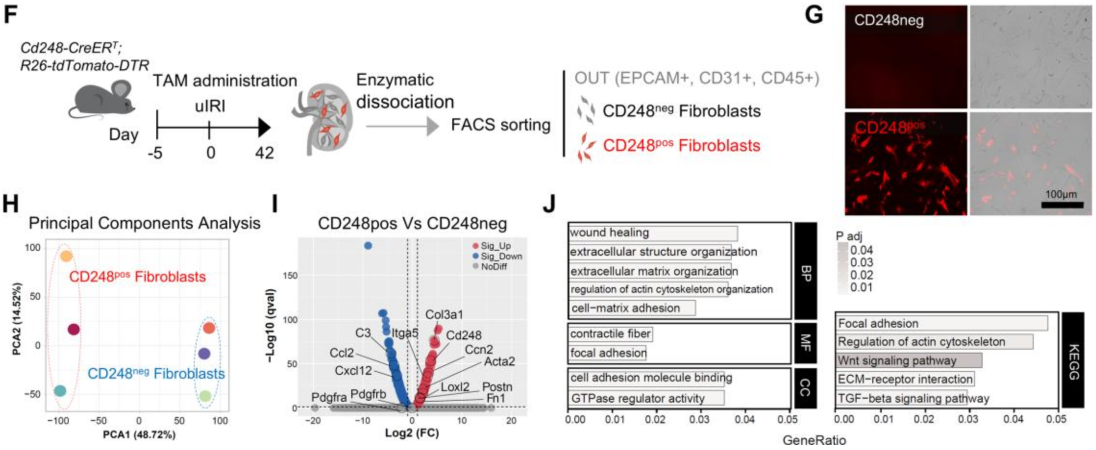

Figure 5O

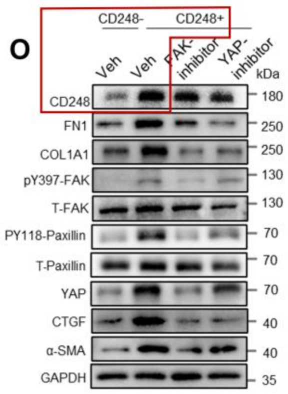

This strategy effectively achieves the core scientific objective of the suggested CITE-seq experiment: explicitly linking the cell identity to high CD248 protein levels and a specific functional state.

2. Protein-level validation in human tissue: To ensure human relevance, our multiplex immunofluorescence data in human CKD biopsies (Figure 1, A and B) demonstrate that CD248 protein spatially co-localizes with key ECM markers (COL1A1, POSTN). This confirms the existence of the CD248+ fibroblast niche at the protein level in human disease, independent of RNA correlations.

3. Genetic and pharmacological functional causality: Beyond correlation, we provide definitive functional proof. Both genetic ablation of *Cd248* and pharmacological blockade with the IgG78 antibody (targeting CD248 protein) significantly ameliorate fibrosis. These results unequivocally demonstrate that the CD248-expressing population is the functional driver of the pathology.

4. Clarification on pericytes: Regarding the comment on pericytes, as detailed in our response to Reviewer #1, our sorting strategy was designed to enrich for interstitial fibroblasts. However, our lineage tracing data suggest that CD248 defines a state of activation that is distinct from quiescent pericyte markers.

In summary, we believe that the combination of lineage-specific protein analysis (FACS and WB), Multiplex immunofluorescence in humans, and functional intervention provides a comprehensive

validation of the CD248+ fibroblast identity, effectively addressing the concern regarding RNA-protein correlation without the need for additional CITE-seq.

[6] Reviewer 2' Comment: [Key markers used to define the ECMhi and i-Fibs subpopulations are poorly justified, with limited evidence supporting their specificity for fibrosis-driving cells.]

Authors' Response: We thank you for this question. While the pro-fibrotic role of POSTN-high fibroblasts (our ECM-Fibs) is well-established, our identification of a distinct immune-modulatory i-Fib state in CKD represents a key conceptual advance. We have now validated this population, which opens exciting new avenues for future research.

The markers used to define the ECM-Fib and i-Fib subpopulations were identified directly from analysis of scRNA-seq data as the top differentially expressed genes that statistically distinguish distinct fibroblast subsets.

In our revised manuscript, we have provided substantial new data to address the justification of our markers directly. Using combined RNAscope and immunofluorescence, we now demonstrate the precise spatial co-localization and mutual exclusivity of these two niches (Figure 1, K to N):

- We confirm that the ECM-Fib state, defined by markers like POSTN, physically co-localizes with its functional output, COL1A1 mRNA.
- Conversely, we show that the i-Fib state, defined by its unique chemokine signature, is spatially associated with CD3+ T cell infiltrates

Figure 1, K to N

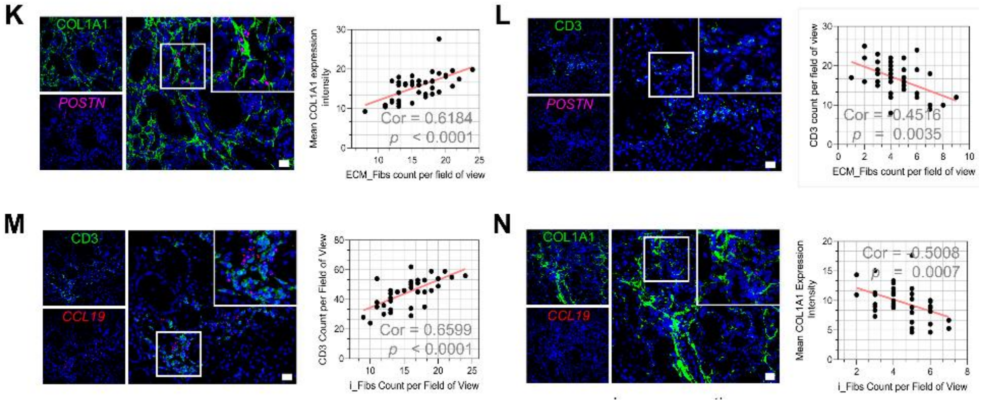

This provides strong, direct evidence for two distinct, spatially segregated, and functionally specialized fibroblast-driven microenvironments in the fibrotic kidney.

Furthermore, our identification of the i-Fib subpopulation is strongly supported by very recent, independent findings in the field. Consistently, a recent spatial multi-omic study (*Single-cell multi-omic and spatial profiling of human kidneys implicates the fibrotic microenvironment in kidney disease progression, Amin Abedini, et al., Nature Genetics, 2024)* described an "immune microenvironment" (IME) characterized by the precise signature we identify in i-Fibs (*CCL19*, *CCL21*, *CXCL12*, as well as fibroblast markers *DCN* and *LUM*, co-localization with T/B cells markers). We have now clarified these points and incorporated this new evidence and discussion into the revised manuscript (Results and Discussion sections).

For in-situ validation of ECM-Fibs and i-Fibs identity, *see lines 182-188. The second paragraph of the Results part "Fibroblasts establish functionally distinct and mutually exclusive pathological niches in injured kidneys": To quantify the relationship between fibroblast subsets and niche composition, we performed combined RNAscope and immunofluorescence analysis. Our data revealed that within local pathological niches, stromal POSTN expression was tightly coupled with COL1A1 deposition while being inversely correlated with CD3+ T cell density (Figure 1, K and L), whereas CCL19 expression was strongly associated with CD3+ T cell infiltration and negatively with COL1A1 deposition (Figure 1, M and N).*

For independent corroboration by a landmark study, *see lines 498-503. The second paragraph of the Discussion part: A recent high-resolution spatial atlas of CKD has revealed distinct microenvironment subtypes of renal stromal areas, often presenting as "mosaic" distribution dominated by fibrogenic microenvironment (FME) and immune microenvironment (IME). The former is characterized by matrix activation markers like POSTN, while the latter is enriched with lymphocytes, as well as chemokines such as CCL19, CCL21, CXCL12 and their receptors(11). In this study, we provided a critical cellular dimension to the establishment of this spatial map. Through integrated ScRNA-seq analysis and multiplex RNAscope, we identified a previously uncharacterized fibroblast subset, termed CCL19&21hi-Fibs, whose localization is strongly correlated with that of CCR7+ lymphocytes, a defining feature of the IME. Besides, our data revealed that ECM-Fibs act as the architects of the FME by remodeling stromal biomechanical properties.*

[7] Reviewer 2' Comment: [The transition of fibroblasts from inflammatory (i-Fibs) to ECM-producing (ECMhi) phenotypes is based solely on trajectory analysis, which is speculative and not experimentally validated. This would have to be shown using e.g. RNAscope or spatial transcriptomics.**]**

Authors' Response: We agree that trajectory analysis is inferential. However, our model of a transition from an i-Fib to an ECM-producing state is strongly supported by multiple lines of evidence.

Our use of pseudotime trajectory analysis to infer a transition from an inflammatory (i-Fib) to an ECM-producing (ECM-Fib) state is a widely accepted computational method in the field. This analytical approach has been instrumental in uncovering fibroblast plasticity in numerous high-impact studies across various pathologies, including cancer and fibrosis. For instance, our group and others have successfully used this exact methodology to demonstrate a similar iCAF-to-myCAF transition in tumors (*1*). A similar method is also applied to various fibrotic studies (*2, 3*).

Besides, this inferred transition is a standard analytical approach that aligns perfectly with the

known pathological progression of fibrosis from an inflammatory to a matrix-depositing phase.

While RNAscope and spatial transcriptomics provide valuable spatial information, they capture a static snapshot and cannot definitively trace dynamic lineage transitions. While excellent for showing co-localization, they cannot provide direct proof of a dynamic cellular state transition over time and would not resolve the inferential nature of the analysis.

Most importantly, our study provides direct experimental validation for the mechanistic driver of this transition. Our *in vitro* hydrogel experiments demonstrate that increasing matrix stiffness is sufficient to drive fibroblasts towards the ECM-producing phenotype predicted by our trajectory (Figure S8, D and E).

Figure S8, D and E

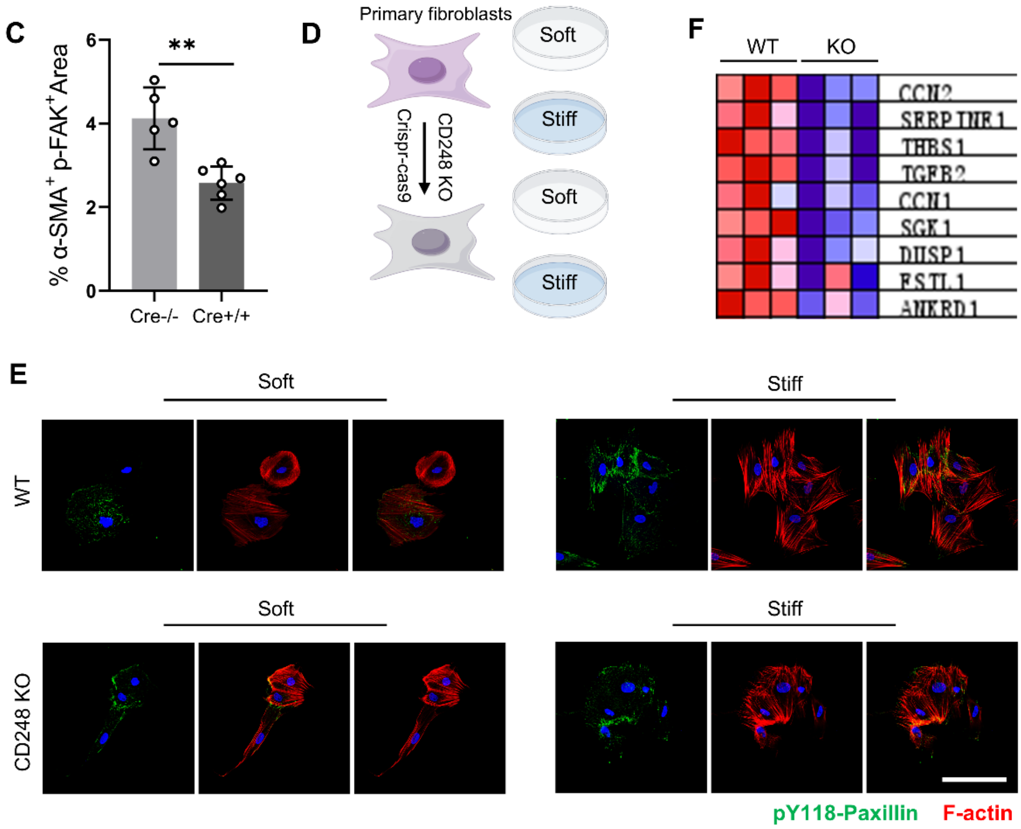

(See lines 357-363. The two paragraphs of the Results part "CD248 remodels the physical microenvironment of the kidney through mechanotransduction-mediated YAP nuclear translocation": <u>To further elucidate the role of CD248 in fibroblast mechanotransduction, we</u>

assessed the response of Cd248-deficient fibroblasts to substrate stiffness. We found that these cells exhibited an impaired response to rigid substrates, as evidenced by diminished focal adhesion formation and suppressed cytoskeletal remodeling, indicating a key role of mechanosignal in CD248-driven myofibroblasts transition (Supplemental Figure 8D and E).)

This provides strong functional support for the directionality and biological relevance of our model. Thus, our conclusion is based on a robust combination of standard bioinformatic inference, a critical understanding of methodological limitations, and direct functional validation of the key underlying mechanism.

Reference:

1 Song H., *et al.* YAP1 Inhibition Induces Phenotype Switching of Cancer-Associated Fibroblasts to Tumor Suppressive in Prostate Cancer. *Cancer Res* 15;84(22):3728-3742. doi: 10.1158/0008-5472.CAN-24-0932 (2024).

2 Kuppe, C., *et al.* Decoding myofibroblast origins in human kidney fibrosis. *Nature* 589, 281-286, doi:10.1038/s41586-020-2941-1 (2021).

3 Gao Y., *et al.* Cross-tissue human fibroblast atlas reveals myofibroblast subtypes with distinct roles in immune modulation. *Cancer Cell* 14;42(10):1764-1783.e10. doi: 10.1016/j.ccell.2024.08.020 (2024).

[8] Reviewer 2' Comment: [The monoclonal antibody IgG78 is described as a promising therapeutic intervention; however: The lack of pharmacokinetic and toxicity data in the in vivo models raises concerns about its safety and feasibility for clinical translation.**]**

Authors' Response: We thank you for raising these critical points regarding the preclinical evaluation of IgG78. We agree that safety and translational feasibility are paramount. We have addressed these concerns by providing new comprehensive safety data and clarifying the scope of our pharmacological characterization.

1. Comprehensive safety profile (new data provided): To directly address the concern regarding potential toxicity, we performed a thorough safety assessment following the therapeutic regimen.

Our new data demonstrate that a full 42-day therapeutic course of IgG78 induced no signs of systemic or organ-specific toxicity.

• Liver/Kidney Function: Serum analysis showed no elevation in ALT/AST or creatinine levels compared to controls (Figure S11, A and B).

• Histopathology: Detailed histological examination of major organs (heart, liver, spleen, lung, kidney) revealed no tissue damage or pathological changes (Figure S11, C and D).

These results provide strong empirical evidence for the favorable biosafety profile of IgG78 in preclinical models.

Figure S11

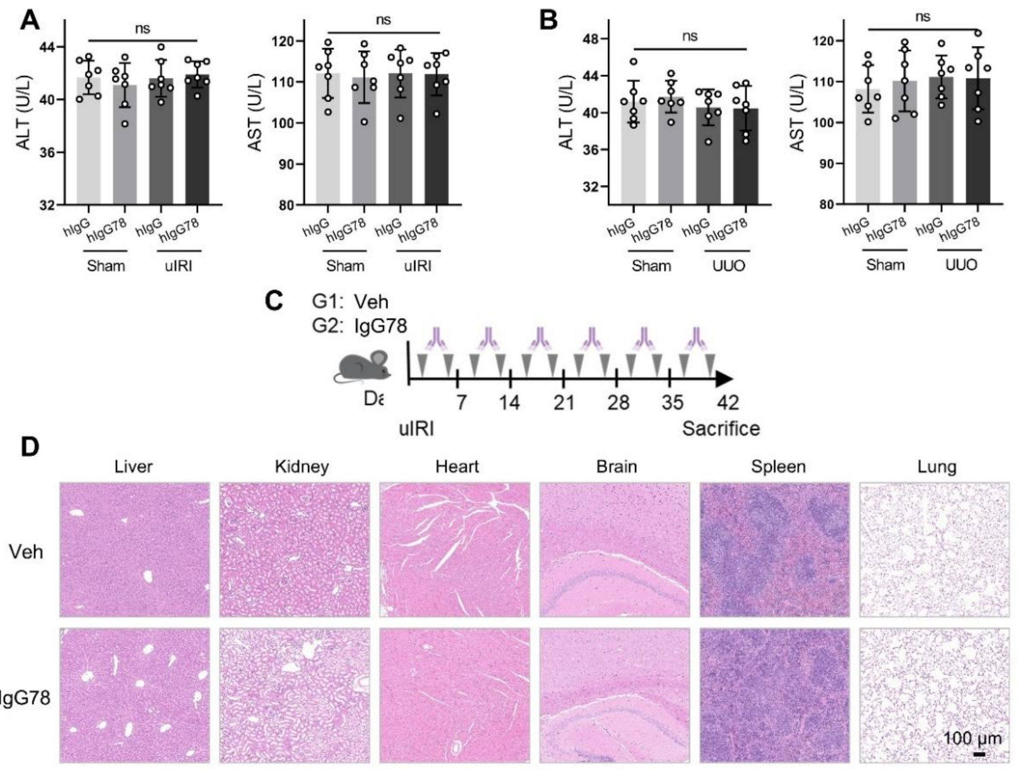

(See lines 476-484. The last paragraph of the Results part "Targeted CD248 monoclonal antibody IgG78 effectively suppresses myofibroblast activation and kidney fibrogenesis in vitro and in vivo": Finally, to assess the safety profile of IgG78, we evaluated its potential hepatotoxicity and systemic toxicity. Given that therapeutic antibodies are primarily metabolized by the liver, we measured serum levels of alanine aminotransferase (ALT) and aspartate aminotransferase (AST) in mice from both fibrosis models. No significant differences

were observed between IgG78-treated and control groups (Supplemental Figure 11, A and B). Furthermore, histological examination of major organs at the end of the treatment cycle revealed no evident tissue damage or pathological changes (Supplemental Figure 11, C and D). Collectively, these data demonstrated that IgG78 exhibits a promising biosafety in preclinical settings.)

2. On pharmacokinetics (PK) and the scope of the study: We appreciate the reviewer's focus on clinical translation.

• **Proof-of-concept scope:** For a discovery-stage study, the primary objective is to establish **safety** and pharmacodynamic (PD) efficacy (i.e., target engagement and anti-fibrotic effect). We have robustly demonstrated both. This aligns with standards for proof-of-concept antibody studies in high-impact journals *(1, 2)*.

• **Target engagement & biodistribution:** We have previously demonstrated the specific targeting capability of IgG78. Our *in vivo* imaging data using a fluorescently-labeled IgG78 (as shown below) *(3)* demonstrates: (i) time-dependent clearance from the animal over 5 days, and (ii) crucially, preferential accumulation in the fibrotic kidney compared to both the contralateral healthy kidney and a non-targeting control antibody. This provides strong, direct evidence of favorable biodistribution and target engagement in the diseased organ.

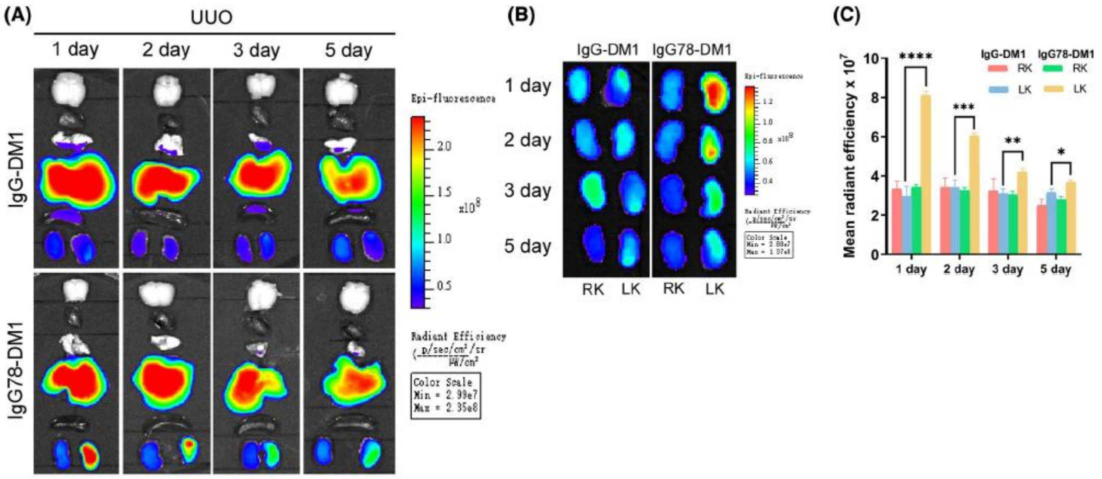

• **Future development:** While we acknowledge the importance of detailed PK parameters (e.g., half-life, clearance volume), it is well-recognized that rodent PK often has limited predictive value for human dosing. Therefore, we have prioritized demonstrating efficacy and safety here, with comprehensive PK studies in non-human primates planned as the next step in formal preclinical development.

Reference:

1 Cheng C., *et al*. Gremlin1 is a therapeutically targetable FGFR1 ligand that regulates lineage plasticity and castration resistance in prostate cancer. *Nat Cancer* 3(5):565-580. doi: 10.1038/s43018-022-00380-3 (2022).

2 Gan L., *et al*. Endosialin-positive CAFs promote hepatocellular carcinoma progression by suppressing CD8$^+$ T cell infiltration. *J Immunother Cancer* 10;12(9):e009111. doi: 10.1136/jitc-2024-009111 (2024).

3 Xu C., *et al*. Antibody-drug conjugates targeting CD248+ myofibroblasts effectively alleviate renal fibrosis in mice. *FASEB J* 36(2):e22102. doi: 10.1096/fj.202101441R (2022).

[9] Reviewer 2' Comment: [The effectiveness of IgG78 is only tested in two mouse models, both of which artificially induce fibrosis. Its impact on more complex or spontaneous CKD models is not addressed.**]**

Authors' Response: We appreciate your perspective on the importance of validating therapies in diverse models. We agree that spontaneous models (e.g., aging, diabetic nephropathy) offer distinct advantages in mimicking the slow, systemic nature of human CKD.

However, after careful consideration, we selected the UUO and uIRI models as the most rigorous tools for the specific objectives of this study: to dissect the molecular mechanism of fibroblast activation and test the direct anti-fibrotic efficacy of IgG78.

We respectfully submit that "spontaneous" models present several specific limitations that would have confounded the interpretation of our results in this context:

• **Confounding systemic pathologies:** Spontaneous models (e.g., STZ-diabetes or Lupus/MRL/lpr) are driven by complex systemic metabolic or inflammatory dysregulation. In such models, it is extremely difficult to distinguish whether a therapeutic effect is due to direct renal fibroblast inhibition (our hypothesis) or secondary to systemic changes (e.g., altered glucose metabolism or systemic immunity). The UUO/uIRI models allow us to isolate the renal fibrotic response without these confounding systemic variables. (*1-2*).

• **Variability and phenotypic robustness:** Spontaneous fibrosis in C57BL/6 mice (the necessary background for our genetic tools) is notoriously mild, slow (often taking >24 months), and highly variable (*3*). This inconsistency makes it statistically challenging to detect the specific impact of a molecular intervention on matrix remodeling. In contrast, UUO/uIRI provide a robust, reproducible fibrotic phenotype that is essential for quantifying the therapeutic window of IgG78.

• **Strain compatibility**: Many spontaneous models (e.g., hypertensive rats) are not compatible with the vast array of genetic tools available in C57BL/6 mice, which were crucial for our mechanistic dissection (e.g., *Cd248-CreERT2* tracing). Furthermore, C57BL/6 mice are known to be resistant to certain spontaneous CKD phenotypes (e.g., hypertension-induced injury), limiting the utility of these models for our specific genetic background (*2*).

While we acknowledge that no single model is perfect, we believe the UUO and uIRI models represent the industry "gold standard" for evaluating anti-fibrotic mechanisms. They allowed us to provide a clean, definitive proof-of-concept for IgG78's efficacy, paving the way for future studies in more complex systemic models.

Reference:

1 Yang HC., *et al*. Models of chronic kidney disease. *Drug Discov Today Dis Models* 7(1-2):13-19. doi: 10.1016/j.ddmod.2010.08.002 (2010).

2 Liang J., *et al*. Animal Models of Kidney Disease: Challenges and Perspectives. *Kidney360* 1;4(10):1479-1493. doi: 10.34067/KID.0000000000000227 (2023).

3 Yabuki A., *et al*. Morphometric study of gender differences with regard to age-related changes in the C57BL/6 mouse kidney. *Exp Anim* 55(4):399-404. doi: 10.1538/expanim.55.399 (2006).

[10] Reviewer 2' Comment: [The authors fail to discuss potential compensatory mechanisms or the redundancy of mechanotransduction pathways that could limit the efficacy of CD248 targeting.**]**

Authors' Response: We agree that this is an important theoretical point to consider.

Our genetic and therapeutic data, however, demonstrate that specific ablation of CD248 is sufficient to markedly attenuate fibrosis and inhibit downstream mechanotransduction in the kidney. This observation leads us to hypothesize that potential signal crosstalk may exist between CD248 and other compensatory pathways, such that targeting it is sufficient to disrupt the broader pro-fibrotic network.

We have now added a brief discussion of this point to the revised manuscript.
(See the line 568-571. The last paragraph of the Discussion part: In this study, we demonstrated that IgG78 represents a promising candidate for therapeutic interventions targeting ECM stiffening and renal fibrosis. Although biological systems possess redundancy, the profound anti-fibrotic effects observed upon either genetic ablation or antibody-mediated blockade of CD248 strongly suggest its role is both dominant and non-redundant in the context of the fibrotic kidney.

[11] Reviewer 2' Comment: [The interactions between CD248+ fibroblasts and other cell types in the niche are not explored, leaving a significant gap in understanding how CD248 contributes to the broader fibrotic process.**]**

Authors' Response: We agree with you that the interplay between fibroblasts and other cell types (e.g., immune cells) is a critical component of the fibrotic niche. However, we respectfully submit that the scope of our current study was specifically designed to elucidate the intrinsic mechanotransduction machinery of the fibroblast and its reciprocal interaction with the ECM.

Our rationale for this focus is two-fold:

1. Defining the core effector mechanism: While immune cells act as upstream activators, myofibroblasts are the primary effectors of scar formation. Our goal was to uncover the core intracellular mechanism that sustains this effector state. By focusing on the intrinsic CD248-YAP axis, we identified a feedback loop that functions autonomously within the fibroblast-matrix unit, independent of transient immune signals.

2. Cell-Matrix vs. Cell-Cell interactions: As the reviewer notes, we have previously explored CD248-mediated fibroblast-macrophage crosstalk in other contexts (e.g., tumor microenvironment, PMID: 32591411). However, in the context of renal fibrosis—where pathological stiffening is a dominant driver—we prioritized investigating cell-matrix interactions. Our data reveal that the physical microenvironment itself acts as a potent signal, and CD248 is the key sensor. This represents a distinct and fundamental layer of regulation that complements classical cell-cell signaling.

Therefore, while we acknowledge that broader cell-cell networks are important, our study intentionally focused on establishing this foundational fibroblast-matrix stiffness mechanism first. Our findings now provide the essential groundwork for future studies integrating this mechanobiology with immune crosstalk.

[12] Reviewer 2' Comment: [The authors make bold claims about the therapeutic potential of targeting CD248 without sufficiently addressing limitations. For instance, while CD248 knockout or depletion reduces fibrosis, it is unclear whether this effect translates into functional improvements in kidney health or protection against CKD progression.**]**

Authors' Response: We appreciate your insistence on verifying functional outcomes. We agree that translating histological fibrosis reduction into tangible renal function preservation is the ultimate goal of any CKD therapy.

1. Correlation between fibrosis and function: Clinically, it is a well-established principle in nephrology that the extent of tubulointerstitial fibrosis is the strongest predictor of renal function decline and CKD progression. Therefore, reducing fibrosis is widely accepted as a primary surrogate endpoint for therapeutic efficacy in preclinical models (*1, 2*).

2. Direct evidence of functional protection (new data): Crucially, to address your comment directly, we have now assessed renal function markers in our therapeutic study. Our new data demonstrate that IgG78 treatment not only reduced matrix deposition but also significantly lowered serum levels of Serum Creatinine (Scr) and Blood Urea Nitrogen (BUN) in the uIRI model (Figure 8J).

This result provides a direct link between the anti-fibrotic effect of CD248 targeting and the preservation of kidney filtration function, thereby substantially strengthening the translational relevance of our findings.

Figure 8J

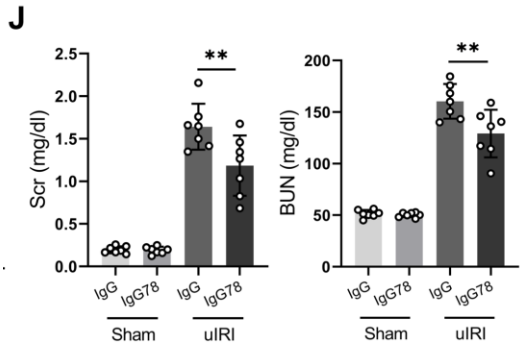

(See the line 463-464. The first paragraph of the Results part "Targeted CD248 monoclonal antibody IgG78 effectively suppresses myofibroblast activation and kidney fibrogenesis in vitro and in vivo": We subsequently aimed to investigate the therapeutic potential of IgG78 and assessed its effects on preclinical models of kidney fibrosis. Following induction of uIRI injury in mice (Figure 8F), our results demonstrated that IgG78 treatment could reduce collagen deposition and FN expression in the renal interstitium (Figure 8, G to I and Supplementary Figure 10A), and *<u>concurrently lowered serum levels of Scr and BUN, key indicators of renal</u>*

function (Figure 8J).)

Reference:

1 Aggarwal S., *et al.* SOX9 switch links regeneration to fibrosis at the single-cell level in mammalian kidneys. *Science* 23;383(6685):eadd6371. doi: 10.1126/science.add6371 (2024).

2 Zhou Q., *et al.* Phenylalanine impairs insulin signaling and inhibits glucose uptake through modification of IRβ. *Nat Commun* 25;13(1):4291. doi: 10.1038/s41467-022-32000-0 (2022).

3 Xu L., *et al.* Tubular GM-CSF Promotes Late MCP-1/CCR2-Mediated Fibrosis and Inflammation after Ischemia/Reperfusion Injury. *J Am Soc Nephrol*. 30(10):1825-1840. doi: 10.1681/ASN.2019010068 (2019).

[13] Reviewer 2' Comment: [The reliance on α-SMA as a myofibroblast marker, despite acknowledging its limitations, undermines the specificity of the conclusions drawn about the role of CD248+ fibroblasts.**]**

Authors' Response: We appreciate the opportunity to clarify this crucial distinction regarding fibroblast heterogeneity. We agree with the reviewer that α-SMA is a broad marker with limited specificity for distinct fibroblast subsets. In fact, this limitation of α-SMA is a central premise of our study.

Our manuscript highlights that while α-SMA marks a broad population of activated interstitial cells, it fails to distinguish the specific, high-matrix-producing subpopulation. Our key finding is precisely that CD248 serves as a superior, high-resolution marker for the specific ECM-high fibroblast state, which α-SMA cannot exclusively define.

Therefore, we utilized α-SMA intentionally to visualize the broad context of myofibroblast activation, against which the specificity of CD248 could be benchmarked. Rather than undermining our conclusions, the broad nature of α-SMA expression actually reinforces our argument: it highlights the urgent need for more specific markers like CD248 to target the true drivers of pathological matrix deposition.

In closing, we would like to express our genuine appreciation for the rigor of your critique. While the comments were undeniably challenging, addressing them has pushed us to generate a substantial body of new data that fundamentally strengthens the translational and mechanistic solidity of our study. We believe this revised manuscript is now a far more robust contribution to the field, and we hope that our extensive revisions have fully resolved the concerns raised.

Reviewer #3

This manuscript provides a comprehensive and innovative exploration of the role of CD248 in renal fibrosis. The authors have identified CD248 as a critical mechanotransduction mediator in fibroblast activation through the IQGAP1-ARF6-GTP pathway, bridging aberrant ECM properties and myofibroblast-driven fibrogenesis. By combining single-cell RNA sequencing, genetic mouse models, and bioinformatics analysis, the study demonstrates the central role of CD248 in sustaining the biomechanical feedback loop within the fibrotic niche. Furthermore, the development and functional validation of a CD248-targeting monoclonal antibody, IgG78, adds substantial therapeutic potential to the findings.

The manuscript is well-written, methodologically rigorous, and aligns with Nature Communications. The work has significant implications for understanding fibrotic disease mechanisms and developing targeted therapeutics. However, there are some issues listed below.

Major question:

[1] Reviewer 3' Comment: It is suggested to measure changes in ECM stiffness in healthy, CKD and CD248 deficient kidneys, and provide additional analyses on CD248's mechanosensory function under varying ECM stiffness levels in vitro to strengthen its role as an ECM sensor.

Authors' Response: We are sincerely grateful for your insightful suggestion. We have now performed both in vivo biophysical measurements and new in vitro functional assays to directly address your points.

1. While our original manuscript already provided evidence for this dynamic in mouse models of CKD and in CD248-deficient kidneys (as shown in Figure 6 G-H, Figure 8M, Figure S9 F-H, and Figure S10M.), your suggestion underscored the critical need for direct evidence from human tissue to establish the pivotal clinical context. Therefore, in direct response to your advice, we have now performed atomic force microscopy (AFM) on fresh human kidney sections. These new experiments provide direct, quantitative evidence that the renal interstitium becomes progressively stiffer as fibrosis advances, establishing the pathologically stiffened niche that our study addresses (as shown

in Figure S5, F to N).

(See the line 217-220. The last paragraph of the Results part "The transition of i-Fibs to ECM-Fibs is driven by mechanosensitive fibroblast activation in response to aberrant stromal properties": Finally, atomic force microscopy (AFM) analysis demonstrated that progressive matrix stiffening accompanies fibrotic progression, implicating aberrant mechanosignaling as a pivotal mechanism driving the activation of ECMhi Fibs and subsequent renal fibrosis (Figure 1R and Supplemental Figure 5, F to N))

Figure S5, F to N

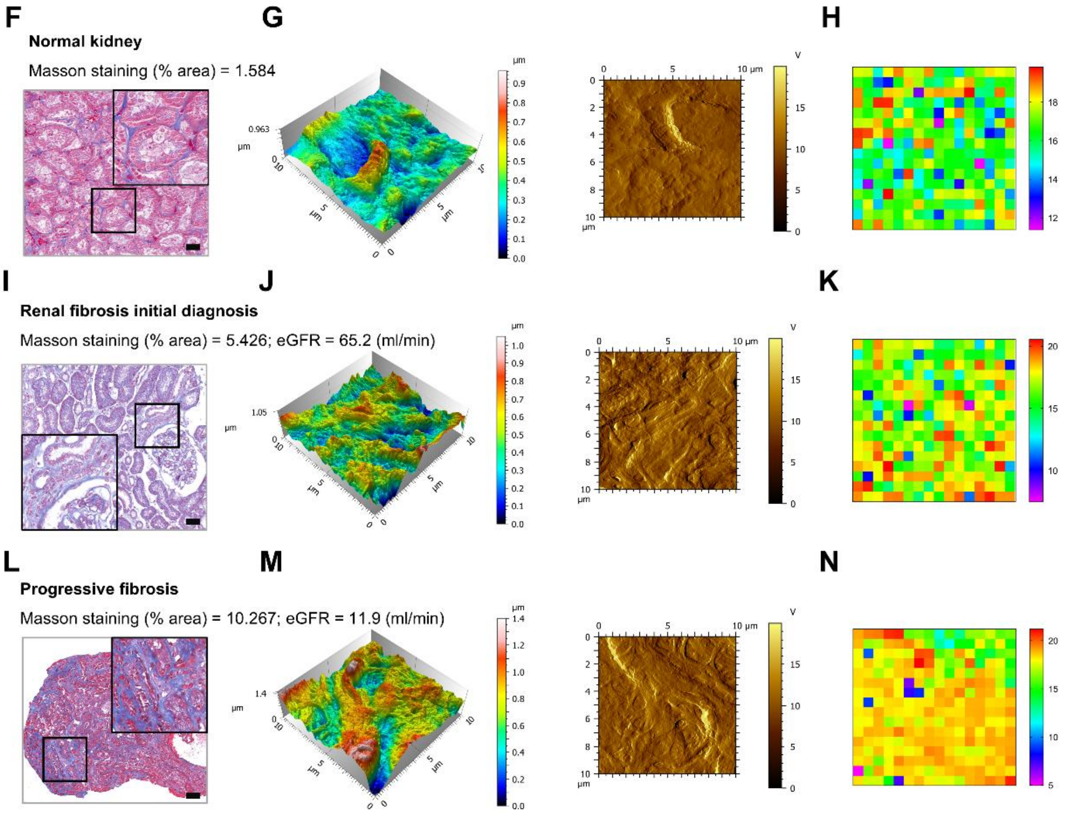

2. To directly test CD248's functional role in sensing this increased stiffness, we cultured primary wild-type and CD248-knockout (KO) fibroblasts on PDMS of controlled stiffness, mimicking 'soft' versus 'stiff' microenvironments. The results unequivocally showed that while WT fibroblasts robustly activated on the stiff substrate, CD248-KO fibroblasts were completely unresponsive to the mechanical cue (as shown in Figure S8 D and E).

Figure S8, D and E

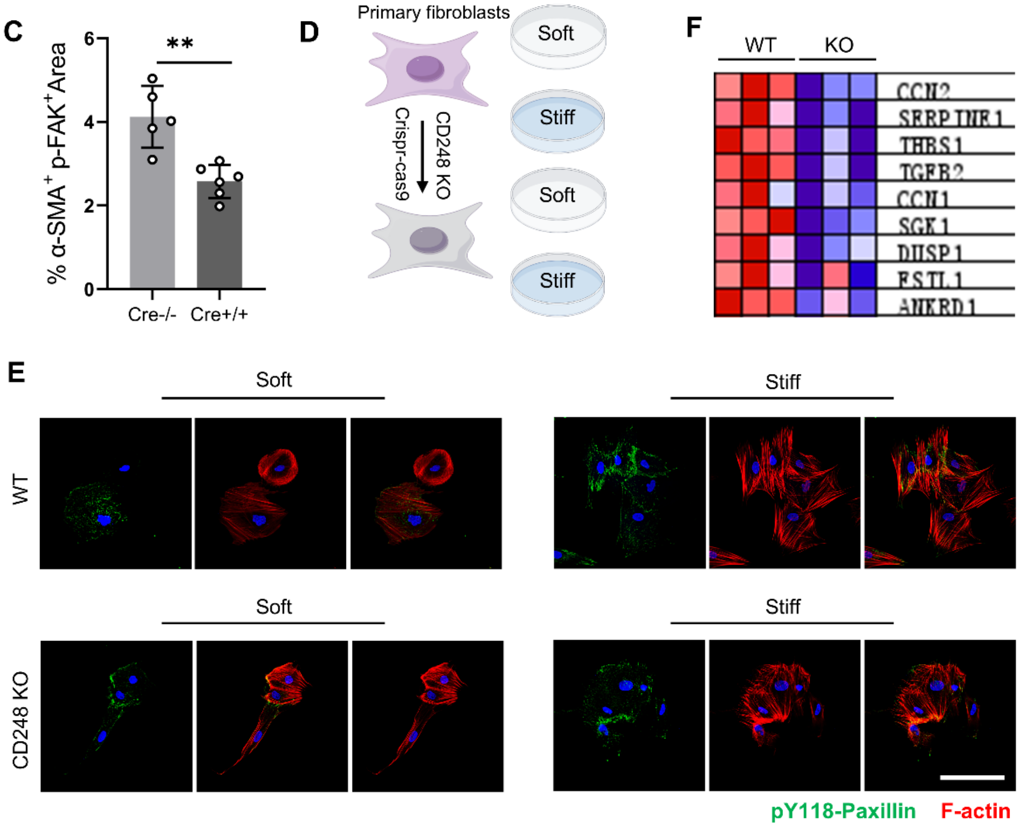

(See the line 357-363. The two paragraph of the Results part "CD248 remodels the physical microenvironment of the kidney through mechanotransduction-mediated YAP nuclear translocation": <u>To further elucidate the role of CD248 in fibroblast mechanotransduction, we assessed the response of Cd248-deficient fibroblasts to substrate stiffness. We found that these cells exhibited an impaired response to rigid substrates, as evidenced by diminished focal adhesion formation and suppressed cytoskeletal remodeling, indicating a key role of mechanosignal in CD248-driven myofibroblasts transition (Supplemental Figure 8D and E).</u>)

Together, these complementary in vivo biophysical measurements and in vitro functional assays forge a complete mechanistic chain: from a pathologically stiffened human ECM to the indispensable role of CD248 as the key cellular sensor that transduces these mechanical cues into a pro-fibrotic response.

We are extremely grateful for your guidance, as these additions have significantly strengthened the clinical relevance, mechanistic depth, and overall impact of our manuscript.

[2] Reviewer 3' Comment: More evidence is needed on the safety of IgG78 therapy, focusing on its potential immunogenicity and effects on critical organs.

Authors' Response: We sincerely thank you for raising this critical point about the preclinical safety of IgG78. To thoroughly address your concerns, we have performed a comprehensive safety evaluation and incorporated these new data into the manuscript.

1. No evidence of organ toxicity: To directly assess systemic safety, we conducted a full 42-day therapeutic course of IgG78. Our new analyses, including serum biochemical analysis (liver function: ALT/AST; kidney function: CREA) and histopathological examination (H&E staining of major organs), revealed no evidence of systemic organ toxicity (new Figure S11). This provides direct, empirical data supporting the safety of our therapeutic antibody.

2. On the point of immunogenicity: You raise a crucial point regarding immunogenicity. While a formal anti-drug antibody assessment was beyond the scope of this initial *proof-of-concept* study, we would like to highlight two key factors that mitigate this concern:
1) Antibody design: IgG78 is a humanized antibody, a design strategy specifically intended to minimize immunogenicity in future clinical applications.
2) Observed tolerability: Importantly, the absence of any observable adverse events in our previous work (*1, 2*), weight loss, or behavioral changes throughout our comprehensive safety studies provides strong indirect evidence of its good tolerability in this preclinical setting.

We are very grateful for your insightful guidance. It has prompted us to significantly strengthen the translational aspects of our manuscript with this essential new data.
(See the line 476-484. The last paragraph of the Results part "Targeted CD248 monoclonal antibody IgG78 effectively suppresses myofibroblast activation and kidney fibrogenesis in vitro and in vivo": <u>Finally, to assess the safety profile of IgG78, we evaluated its potential hepatotoxicity and</u>

systemic toxicity. Given that therapeutic antibodies are primarily metabolized by the liver, we measured serum levels of alanine aminotransferase (ALT) and aspartate aminotransferase (AST) in mice from both fibrosis models. No significant differences were observed between IgG78-treated and control groups (Supplemental Figure 11, A and B). Furthermore, histological examination of major organs at the end of the treatment cycle revealed no evident tissue damage or pathological changes (Supplemental Figure 11, C and D). Collectively, these data demonstrated that IgG78 exhibits a promising biosafety in preclinical settings.)

Figure S11

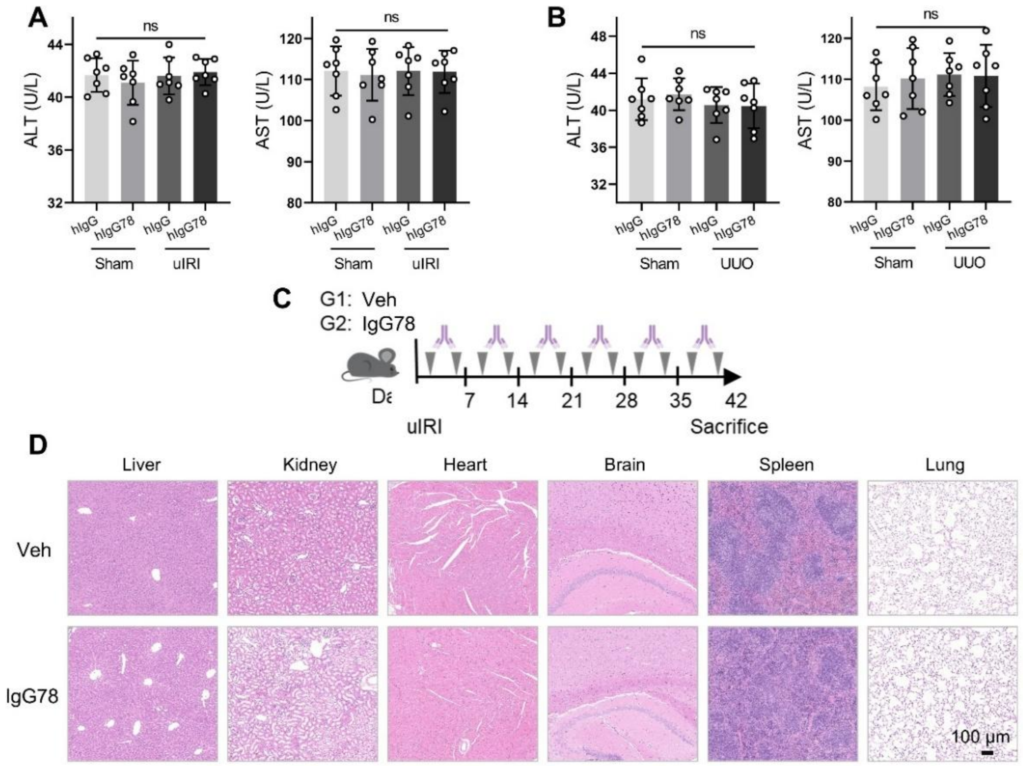

Reference:

1 Xu C., et al. Antibody-drug conjugates targeting CD248+ myofibroblasts effectively alleviate renal fibrosis in mice. FASEB J. 36(2):e22102. doi: 10.1096/fj.202101441R (2022).

2 Liu S., et al. Antibody-drug conjugates targeting CD248 inhibits liver fibrosis through specific killing on myofibroblasts. Mol Med. 22;28(1):37. doi: 10.1186/s10020-022-00460-1 (2022).

[3] Reviewer 3' Comment: Although the effects of CD248 and IgG78 have been validated in animal models, systematic validation in human tissue samples is lacking. Validation experiments of IgG78 in vitro human cell lines or organoids can be added, and associations between CD248 expression levels and clinical indicators such as kidney function in samples from CKD patients can be systematically analyzed.

Authors' Response: In direct response to your suggestion, we have performed two key sets of experiments and analyses, which have been incorporated into the revised manuscript.

1. Direct therapeutic validation in a human cell model: To address your first point, we tested the therapeutic efficacy of our antibody, IgG78, directly on a human cell line. We used the human fibroblast line HFL1 and quantified collagen deposition via second harmonic generation (SHG) imaging. The results were definitive: IgG78 treatment significantly inhibited collagen production by these human cells, demonstrating that its anti-fibrotic mechanism is conserved and effective in a human context (as shown in Figure 8E).

(See the line 454-455. The first paragraph of the Results part "Targeted CD248 monoclonal antibody IgG78 effectively suppresses myofibroblast activation and kidney fibrogenesis in vitro and in vivo": To directly assess the anti-fibrotic potential of IgG78, we leveraged its cross-reactivity to test effects on both murine and human fibroblasts. IgG78 treatment significantly suppressed collagen production in primary murine fibroblasts and, importantly, in the human fibroblast cell line HFL1, highlighting its therapeutic promise. We found that CD248 inhibition by IgG78 significantly reduced collagen deposition (Figure 8, D and E).)

Figure 8E

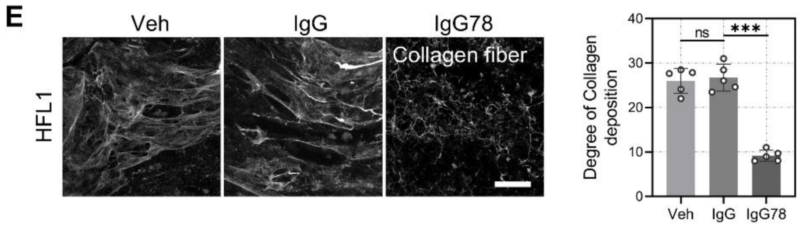

2. Correlation with clinical severity in CKD patients: To address your second point, we performed a new systematic analysis on our cohort of human CKD patient samples. This quantitative analysis revealed a statistically significant positive correlation between the CD248+ interstitial area and the severity of renal dysfunction, as measured by a decrease in patient eGFR (*Cor = 0.3761, p = 0.0047*). This provides direct clinical evidence linking higher CD248 expression to worse patient outcomes (as shown in Figure 2, F and G).

(See the line 244-247. The Results part "CD248+ myofibroblasts represent the terminal fibroblasts subtype responsible for stromal remodeling in CKD patients": <u>CD248 was selectively co-localized with COL1A1 in the non-epithelial stroma. Notably, in healthy control tissues, stromal CD248 expression was barely detectable in the interstitium. Furthermore, CD248 expression correlated significantly with renal collagen deposition (Figure 2F), and negatively with renal function (Figure 2G).</u>)

Figure 2, F and G

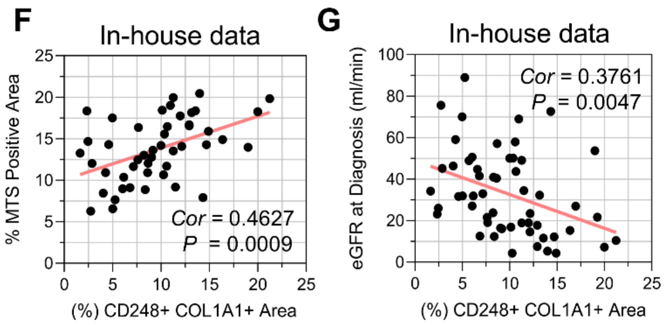

Taken together, these two new lines of evidence, direct therapeutic validation in human cells and a robust clinical correlation in patient tissues, substantially strengthen the translational impact of our study. We are sincerely thankful for your guidance, which has been instrumental in elevating the quality and significance of our work.

[4] Reviewer 3' Comment: In Figure 8, fibrosis-related markers like α-SMA, YAP, and renal function such as the estimated glomerular filtration rate after IgG78 treatment should be characterized.

Authors' Response: In direct response to your feedback, we have performed these new analyses and incorporated the data into the revised manuscript.

1. Inhibition of myofibroblast activation: We performed co-immunofluorescence staining for α-SMA and phosphorylated FAK. As shown in our new Figure 8K, uIRI injury led to a dramatic increase in α-SMA+ p-FAK+ myofibroblasts in the interstitium. Treatment with IgG78 significantly reduced the population of these activated, mechanosignaling-competent cells, providing direct evidence that our antibody blocks myofibroblast activation at the cellular level.

2. Suppression of the downstream mechanotransduction pathway: To confirm that IgG78 disrupts the mechanosensory signaling cascade, we evaluated the subcellular localization of YAP, a critical mechanosensitive transcriptional co-activator. As demonstrated in our new Figure 8L, treatment with IgG78 markedly inhibited this YAP nuclear translocation but not IgG, indicating that the antibody effectively blunts the downstream pro-fibrotic signaling initiated by mechanical stress.
(See the line 464-470. The first paragraph of the Results part "Targeted CD248 monoclonal antibody IgG78 effectively suppresses myofibroblast activation and kidney fibrogenesis in vitro and in vivo": We next explored the effects of IgG78 on mechanotransduction-mediated fibroblast activation in vivo. Indeed, immunofluorescence staining revealed that treatment targeting CD248 suppressed stromal activation, as evidenced by decreased levels of α-SMA and FAK-pY397 (Figure 8K and Supplementary Figure 10B). Moreover, IgG78 administration also led to reduced CD248 expression and nuclear translocation of YAP within CD248+ myofibroblasts (Figure 8L and Supplementary Figure 10C).)

Figure 8, K and L

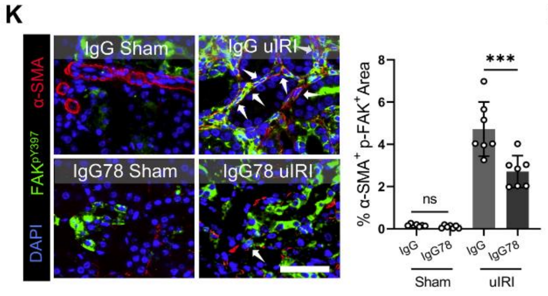

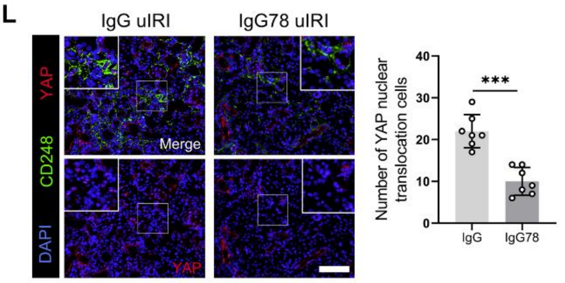

3. Improvement in renal function: Finally, to assess the overall impact on organ function, we evaluated serum creatinine (Scr) and blood urea nitrogen (BUN) levels. The data, now included in the revised Figure 8J, show that IgG78 treatment led to a significant improvement in renal function compared to the IgG control group.

(See the line 463-464. The first paragraph of the Results part "Targeted CD248 monoclonal antibody IgG78 effectively suppresses myofibroblast activation and kidney fibrogenesis in vitro and in vivo": We subsequently aimed to investigate the therapeutic potential of IgG78 and assessed its effects on preclinical models of kidney fibrosis. Following induction of uIRI injury in mice (Figure 8F), our results demonstrated that IgG78 treatment could reduce collagen deposition and FN expression in the renal interstitium (Figure 8, G to I and Supplementary Figure 10A), and concurrently lowered serum levels of Scr and BUN, key indicators of renal function (Figure 8J).)

Figure 8J

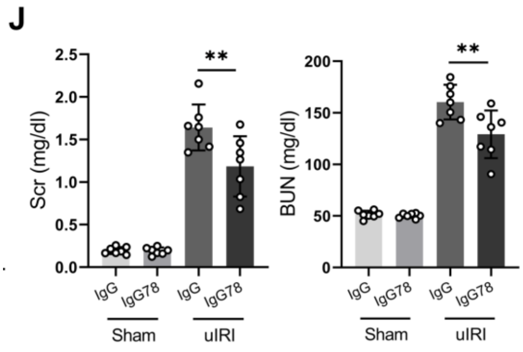

[5] Reviewer 3' Comment: A more intuitive schematic of the molecular mechanism of CD248 or the whole story would be better.

Authors' Response: We agree that a summary schematic would greatly help clarify the molecular

mechanism of CD248. Accordingly, we have now added the corresponding figure and its legend (Figure 7R) to the revised manuscript.

*(See the line 971-975. The legend part of Figure 7R: __(**R**) Schematic illustrating the proposed positive feedback loop driving fibrogenesis. Upregulation of CD248 on fibroblasts promotes IQGAP1/ARF6-YAP mechanosignaling, leading to ECM deposition and fibroblast activation. The resulting matrix stiffening, in turn, further enhances CD248-mediated signaling, creating a self-perpetuating fibrotic loop.__*

Figure 7R

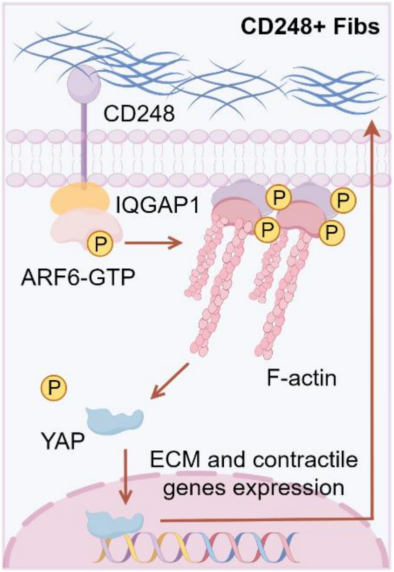

And some other minor issues:

[6] Reviewer 3' Comment: Figure 1A and Supplemental Figure 9A do not seem to be mentioned in the text.

Authors' Response: We sincerely apologize for this oversight and thank you for your meticulous review. In the revised manuscript, we have now added the appropriate citations for both Figure 1A and Supplemental Figure 9A in the relevant sections. Moreover, prompted by your comment, we have performed a comprehensive check of the entire manuscript to ensure that all figures, tables, and supplementary materials are correctly and consistently referenced. We appreciate your help in

improving the clarity and accuracy of our work.

[7] Reviewer 3' Comment: In Figure 2E, when EPCAM first appeared, what did it stand for.

We thank you for this excellent question and for pointing out the lack of clarity in our original figure legend.

Authors' Response: You are correct that we failed to properly define EPCAM upon its first use. "EPCAM" stands for epithelial cell adhesion molecule, a well-established marker for tubular epithelial cells. To address this and improve clarity, we have revised the figure legend to be more precise.

(See the line 806-807. The Figure legend part (Figure.2E): Representative immunofluorescent staining of specified proteins in fibrotic kidney. EPCAM, a marker for tubular epithelial cells, and COL1A1, a marker for renal interstitium area. Scale bar, 100 μm.)

[8] Reviewer 3' Comment: The zoom image in the picture also needs a scale bar.

Authors' Response: In response to your suggestion, we have carefully reviewed all figures and have now added the appropriate scale bars to every magnified panel throughout the manuscript. We appreciate your help in enhancing the clarity and scientific rigor of our figures.

[9] Reviewer 3' Comment: It is suggested to put the more intuitive staining data in the main figures, and put its statistical analysis data in the supplemental figures.

Authors' Response: We completely agree that prioritizing the most intuitive visual evidence in the main figures, while moving the corresponding statistical analyses to the supplementary materials, will significantly enhance the narrative flow and readability of our manuscript.

Previous Figure 5 **Present Figure 5**

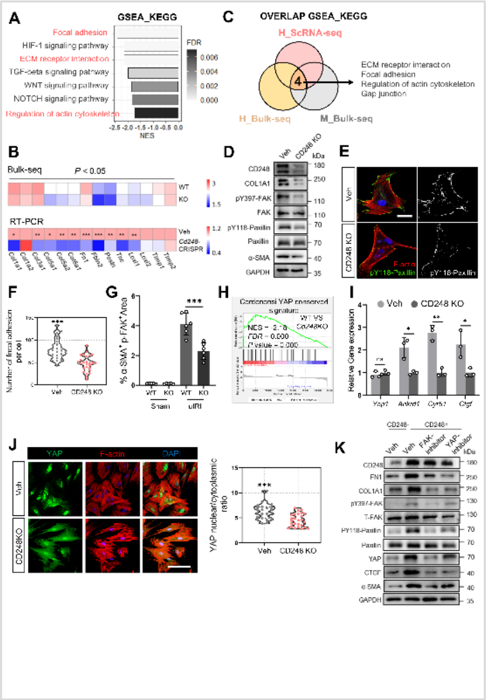

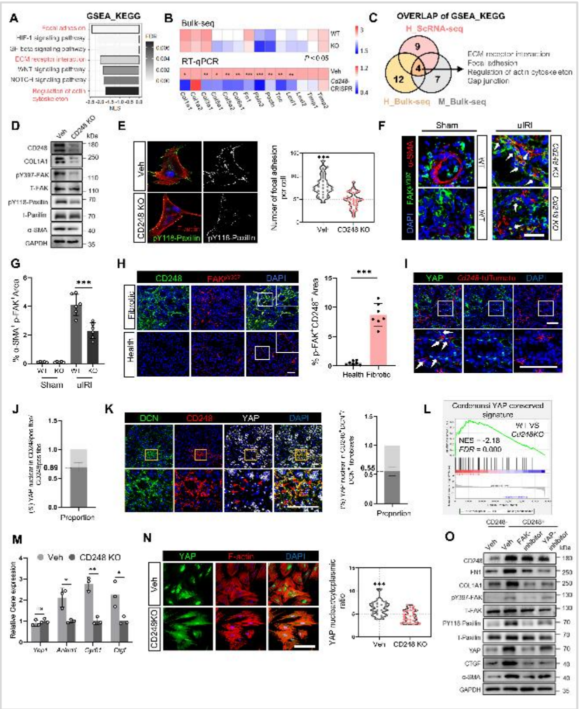

Previous Figure 7

Present Figure 7

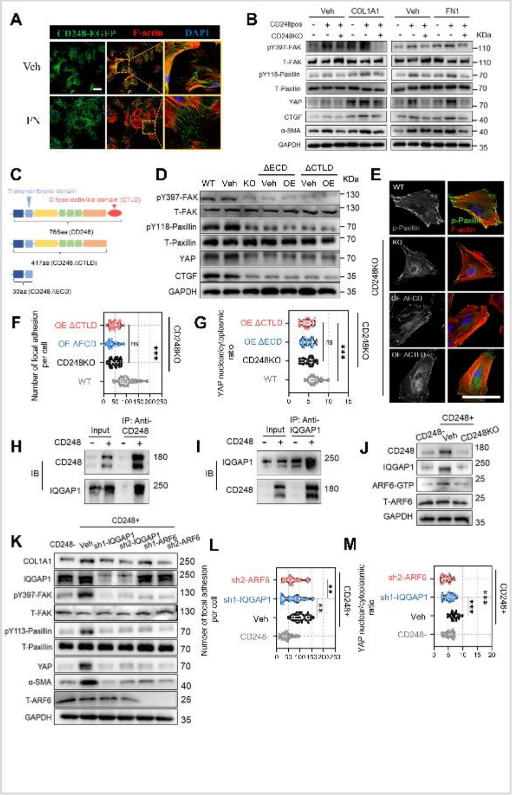

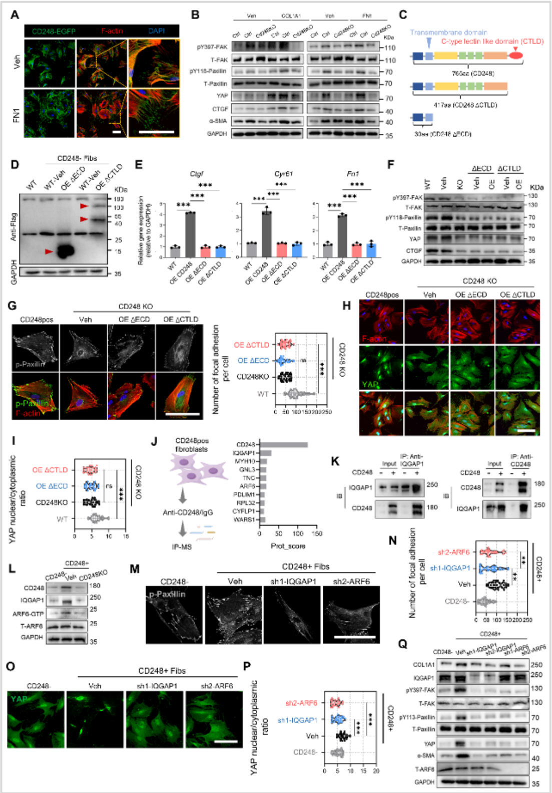

[10] Reviewer 3' Comment: Should the figure 1I caption refer to gene cluster 2?

Authors' Response: The original label was ambiguous. We have revised it to clarify that the figure refers to a gene cluster from the trajectory analysis, not a cell cluster. The title in Figure 1I has been updated as follows:

Original: Cluster 2 Top KEGG Enrichment

Revised: Gene Cluster 2 Top KEGG Enrichment

Figure 1P (Previous Figure 7I)

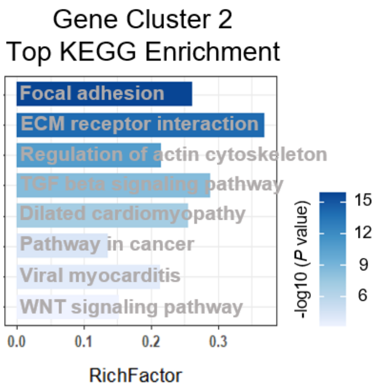

This change has been made in the revised manuscript to ensure clarity.

[11] Reviewer 3' Comment: The label of FAK, T-FAK; Paxillin, T-Paxillin in the Western blot should be unified.

Authors' Response: The labeling for total proteins in our Western blot figures was inconsistent. We have now revised all relevant figures to uniformly use "T-FAK" for Total FAK and "T-Paxillin" for Total Paxillin to ensure consistency.

Reviewer 3' Comment: Together, this manuscript identified CD248 as a pivotal player in ECM-myofibroblast interactions and the therapeutic validation of IgG78 are of high scientific and clinical relevance.

This paper can be accepted with minor revisions. Addressing the outlined suggestions will further enhance the manuscript's impact and translational potential.

Finally, we would like to express our sincere gratitude for your thorough and insightful review. Your constructive comments, particularly regarding the need for biophysical and translational evidence, were instrumental in guiding our revisions. We are confident that your suggestions have not only addressed the key questions but have also significantly elevated the overall quality, mechanistic depth, and impact of our manuscript.

Responses to Reviewers’ Comments for Manuscript NCOMMS-24-83524A-Z

Point-by-point reply

Reviewer #3

The authors have revised the manuscript and addressed most of the previous concerns. The overall clarity and presentation of the study have improved. The addition of the schematic model and reorganization of several figures have strengthened the narrative of the manuscript.

However, several issues still require clarification to further improve the rigor and completeness of the work.

Reviewer question:

[1] Reviewer 3’ Comment: The proposed positive feedback loop involving matrix stiffening and CD248 activation is conceptually compelling. The authors may consider clarifying whether direct experimental evidence supports stiffness-induced CD248 upregulation, or whether this remains a model-based interpretation.

Authors’ Response: We agree that distinguishing between stiffness-induced expression versus functional activation is crucial.

In our ongoing studies using parallel disease model, we have further investigated the relationship between physical stiffness and CD248 expression. Regrettably, our data indicate that matrix stiffness does not directly regulate CD248 expression levels.

Given that this negative finding is beyond the scope of the current manuscript and does not affect the core conclusions regarding the role of CD248 in mechanotransduction, we have decided not to include these additional data in the revised text.

[2] Reviewer 3' Comment: The therapeutic mechanism of IgG78 could be briefly discussed in more detail, particularly whether the antibody primarily blocks ECM binding or modulates receptor signaling/clustering.

Authors' Response: We thank the reviewer for this suggestion. We have now briefly expanded the Discussion to address the potential therapeutic mechanism of IgG78, speculating that it likely functions by blocking the interaction between CD248 and the ECM, potentially targeting the CTLD domain.

(See the line 572-576. The final paragraph of the Discussion part: In this study, we demonstrated that IgG78 represents a promising candidate for therapeutic interventions targeting ECM stiffening and renal fibrosis. Although biological systems possess redundancy, the profound anti-fibrotic effects observed upon either genetic ablation or antibody-mediated blockade of CD248 suggest its role is both dominant and non-redundant in the context of the fibrotic kidney. Mechanistically, we speculate that IgG78 functions by physically blocking the interaction between CD248 and the disordered ECM, potentially targeting the CTLD domain. However, the precise structural mechanism, whether it primarily involves inhibition of ligand binding or receptor clustering, remains to be elucidated in future studies.)

[3] Reviewer 3' Comment: The causal relationship between CD248 and IQGAP1-ARF6 signaling would benefit from future genetic epistasis validation, which could be acknowledged as a limitation.

Authors' Response: We agree that genetic epistasis experiments would provide the most definitive proof of causality for this signaling axis. As suggested, we have added a sentence to the Discussion section acknowledging this limitation and pointing towards future genetic studies to solidify the hierarchy between CD248 and IQGAP1-ARF6 signaling.

(See the line 558-561. The final paragraph of the Discussion part: Nevertheless, direct evidence clarifying ARF6 as a mediator of fibroblast activation during fibrosis progression is still lacking.

Our data revealed that CD248 recruits the active GTP-bound form of ARF6 via the scaffold protein IQGAP1, thereby promoting downstream FAK/Paxillin-YAP signaling, myofibroblast activation, and stromal remodeling. However, we acknowledge that while our data support this biochemical interaction, establishing a definitive causal hierarchy between CD248 and the IQGAP1-ARF6 axis would benefit from future validation using genetic epistasis models.)

[4] Reviewer 3' Comment: Figure S8 E needs to include fluorescence intensity data. Currently, the differences appear to be insignificant.

Authors' Response: We thank the reviewer for this careful observation. As requested, we have performed quantitative analysis of the pY118-paxillin fluorescence intensity (specifically, the average positive staining area per cell). The results confirm that the differences between groups are indeed statistically significant.

We have updated Figure S8E to include this quantification graph and revised the figure legend accordingly.

Figure S8E

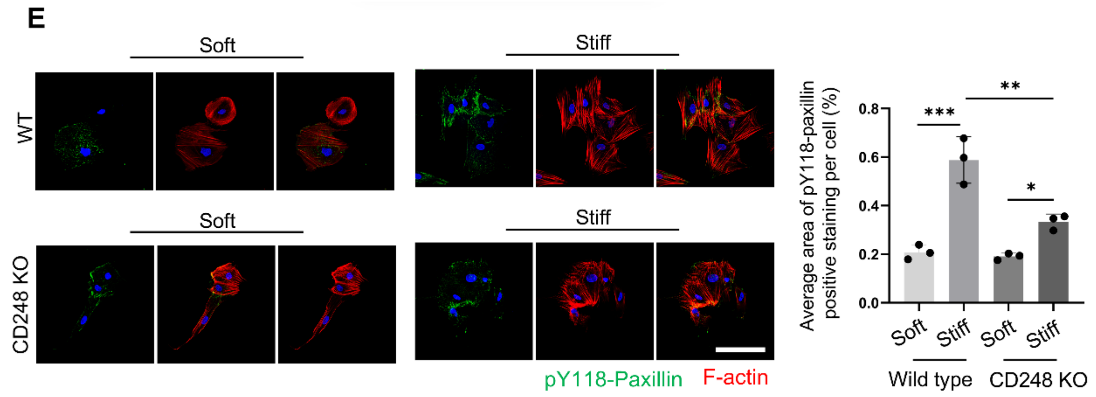

***(E)** Immunofluorescence analysis of pY118-paxillin and F-actin among primary renal fibroblasts with or without CD248KO in soft or stiff substrates. Scale bar, 100 μm. The right panel shows the quantification of the average area of pY118-paxillin positive staining per cell (%).*
